# Questionnaires measuring movement behaviours in adults and older adults: Content description and measurement properties. A systematic review

**DOI:** 10.1371/journal.pone.0265100

**Published:** 2022-03-11

**Authors:** Bruno Rodrigues, Jorge Encantado, Eliana Carraça, Eduarda Sousa-Sá, Luís Lopes, Dylan Cliff, Romeu Mendes, Marlene Nunes Silva, Cristina Godinho, Rute Santos

**Affiliations:** 1 Faculty of Sport, University of Porto (Research Centre in Physical Activity, Health and Leisure), Porto, Portugal; 2 Directorate-General of Health, National Physical Activity Promotion Program, Lisbon, Portugal; 3 CIPER, Faculdade de Motricidade Humana, Universidade de Lisboa, Lisbon, Portugal; 4 Faculdade de Educação Física e Desporto, CIDEFES, Universidade Lusófona de Humanidades e Tecnologias, Lisboa, Portugal; 5 Early Start, School of Education, Faculty of the Arts, Social Sciences and Humanities, University of Wollongong (Illawarra Health and Medical Research Institute), Wollongong, NSW, Australia; 6 Laboratory for Integrative and Translational Research in Population Health, Porto, Portugal; 7 EPIUnit-Instituto de Saúde Pública, Universidade do Porto, Porto, Portugal; 8 Northern Region Health Administration, Porto, Portugal; 9 Católica Research Centre for Psychological—Family and Social Wellbeing, Universidade Católica Portuguesa, Lisbon, Portugal; Mugla Sitki Kocman Universitesi, TURKEY

## Abstract

**Background:**

Sleep, sedentary behaviour and physical activity are constituent parts of a 24h period and there are several questionnaires to measure these movement behaviours, the objective was to systematically review the literature on content and measurement properties of self- and proxy-reported questionnaires measuring movement behaviours in adults and older adults.

**Methods:**

The databases PubMed, CINAHL, PsycINFO and SPORTDiscus were systematically searched until April 2021. Articles were included if: the questionnaires were design for adults and older adults; the sample size for validity studies had at least 50 participants; at least, both validity and test-retest reliability results of questionnaire that were developed specifically to measure the amount of sleep, sedentary behaviour or physical activity, or their combination were reported; and articles had to be written in English, Spanish, French, Portuguese, German, Italian or Chinese.

**Findings and conclusions:**

Data extraction, results, studies’ quality, and risk of bias were evaluated using the Consensus-based Standards for the selection of health Measurement INstruments (COSMIN) guidelines. Fifty-five articles were included in this review, describing 60 questionnaires. None of the questionnaires showed adequate criterion validity and adequate reliability, simultaneously; 68.3% showed adequate content validity. The risk of bias for criterion validity and reliability were very low in 72.2% and 23.6% of the studies, respectively. Existing questionnaires have insufficient measurement properties and frequent methodologic limitations, and none was developed considering the 24h movement behaviour paradigm. The lack of valid and reliable questionnaires assessing 24h movement behaviours in an integrated way, precludes accurate monitoring and surveillance systems of 24h movement behaviours.

## 1. Introduction

In light of the recent 24h movement behaviour paradigm [1], sleep, sedentary behaviour (SB) and physical activity (PA) are constituent parts of a 24h period that interact and influence health. This new paradigm has led some countries, as well as the World Health Organization (WHO) to develop 24h movement guidelines [2–4]. With its development and launching in other countries there is a tangible need to accurately assess movement behaviours in an integrated way; and monitoring and surveillance systems will need to be adapted to assess compliance with such guidelines. The accurate assessment of movement behaviours is also essential for research, policy, and practice. Despite the advantages of objective methods to assess movement behaviours, such as accelerometery (e.g., do not depend on participant recall) in large epidemiological studies and clinical settings, self- or proxy-reported questionnaires are often preferred, given their practicality, simplicity, affordability, and low burden for participants (in terms of time consuming and acceptability) [5–7]. Moreover, these are capable of gathering valuable contextual information (e.g., domains, settings, types) of the behaviours, that objective measures are unable to [8]. Nevertheless, assessing 24h movement behaviours is challenging and complex, given that movement behaviours questionnaires are often prone to measurement errors and reporting bias due to misreporting, whether due to social desirability bias or cognitive issues related to recall or comprehension [9].

The usefulness of a self-reported measure is dictated by its qualitative attributes (i.e., content validity) and psychometric properties, such as test–retest reliability and criterion validity. As such, questionnaires must be adequately developed and described, presenting adequate content and measurement properties, because if the development method and the measurement properties are weak or not extensively known, the risk of misclassification, biased and unreliable results is high [10].

The self-reported assessment of movement behaviours has generally been done by assessing each behaviour *per se* and consequently, evidence of the content analysis and measurement properties of the instruments used to assess these behaviours has also been done in isolation. Recently, two systematic reviews [11, 12] on measurement properties of PA questionnaires reported several limitations, particularly related to statistical methods and accelerometery interpretation; and that the methodological quality of the studies could be improved by increasing sample size, enhancing statistical procedures and reporting methods, and choosing better comparison measures for validity studies. Regarding SB, two other systematic reviews [13, 14] reported poor levels of agreement and accuracy with under and overestimation of total time spent in SB. Altogether, these reviews indicate that precise self-report instruments to measure PA and SB are still scarce [15]. Concerning sleep questionnaires, these seem to be primarily used as a diagnostic tool and to be relatively accurate [16]. Despite the reduced accuracy when compared with diaries and objective instruments, questionnaire-based data is considered relevant due to the importance of each person’s self-perception about their sleep [16]. However, it is unclear whether there are questionnaires assessing sleep considering it as part of a 24h period (i.e., as a movement behaviour).

The fact that movement behaviours have traditionally been subjectively assessed individually (each behaviour *per se*) and ignoring the intrinsic and empirical interactions between them [17, 18], may partly be because there is no single questionnaire that assesses 24h movement behaviours in an integrated way. Selecting the best questionnaire for each movement behaviour (or their combination) is difficult, given the high variability in their content and the inadequate measurement properties. This has been documented in previous reviews [11, 12, 14, 16, 19, 20]. However, none of these reviews assessed the questionnaires that measure the combination of these behaviours at the same time. Therefore, reviewing the questionnaires measuring all the movement behaviours, individually or in combination, in adults and older adults, is necessary. In this context, we aimed to systematically review the literature on content and measurement properties of self- and proxy-reported questionnaires measuring the movement behaviours or its combination, in adults and older adults.

## 2. Materials and methods

### 2.1 Information sources and search strategy

A systematic search through the electronic databases PubMed, CINAHL, PsycINFO and SPORTDiscus was conducted in April 2021, from inception until April 2021. Additional studies were identified by manually searching references of the retrieved papers.

The electronic databases were searched for variations of the terms ‘PA’, ‘SB’, ‘sleep’, ‘movement behaviours’, ‘questionnaire’ and ‘measurement properties’. A supporting file shows this in more detail [see S1 File]. The search terms used for ‘measurement properties’ were the ones proposed by COSMIN guidelines [21]. The search terms were adapted for each specific electronic database to ensure the quality of the systematic searching (e.g., in PubMed’s case, MESH terms were used when applicable).

### 2.2 Eligibility criteria

Consensus-based Standards for the selection of health Measurement INstruments (COSMIN) guidelines for systematic reviews of patient-reported outcome measures [21], were adapted to the purpose of this review and followed. The COSMIN guidelines are in concordance with the Cochrane Handbook for systematic reviews of interventions [22] and the Preferred Reporting Items for Systematic Reviews and Meta-Analyses (PRISMA) [23].

To identify and characterize valid and reliable self-reported or proxy-reported questionnaires assessing sleep, sedentary behaviour and physical activity, or their combination, the following inclusion criteria were defined: 1) participants were adults (≥ 18 years) or older adults (≥65 years), living in the community; 2) minimum sample size of 50 participants for validity studies [24]; 3) articles reporting at least, both validity and test-retest reliability results [25] of questionnaire that were developed specifically to measure the amount of sleep, SB or PA, or its combination; 4) articles written in English, Spanish, French, Portuguese, German, Italian or Chinese.

The exclusion criteria were the following: 1) articles that used doubly labelled water as gold standard for validity purposes, given that doubly labelled water assesses total energy expenditure, not only PA energy expenditure and, as such, it has been considered an unreliable criterion measure for PA levels [11, 25]; 2) reporting measurement properties of instruments that aimed solely to predict or detect a given health condition, designed for special populations (e.g., chronic, auto-immune and infectious diseases, sleep disorders, athletes, pregnant women) or focused only on lifetime PA; 3) reporting measurement properties of questionnaires that were not designed to validate an original questionnaire (e.g. reported linguistic validation); 4) articles reporting measurement properties of logs, diaries or interviews of movement behaviours; 5) grey literature (e.g. policy reports; government documents; working papers; conference proceedings; thesis and books or book chapters), reviews, meta-analyses, cost-effectiveness studies and commentaries.

### 2.3 Study selection process

Three authors (BR, JE and EVC) independently screened articles by title, abstract and full text. Results were cross-checked and disagreements were resolved by discussion with a fourth author (RS), until consensus was reached. Reference lists of identified articles were also reviewed to ensure that no relevant articles were overlooked. These processes were conducted using the CADIMA software [26].

### 2.4 Data collection process and data items

A standardized data extraction form was created to record relevant information from the included articles about the questionnaires’ content, validity, reliability, measurement error and responsiveness. A supporting file shows this in more detail [see S2 File].

Given the characteristics of this review, the data extraction on content and measurement properties was based on the COSMIN guidelines [21], the Taxonomy of Self-reported SB Tools (TASST) framework [13] and the Quality Assessment of PA Questionnaire Checklist (QAPAQ) [25]. For measurement properties, the Edinburgh Framework for validity and reliability in PA and SB measurement was also considered [27]. When needed, adaptations have been made to integrate sleep as a movement behaviour. The measurement properties’ definitions used in this study are presented in Table 1.

**Table 1:** Measurement properties definitions.

1. Measurement Property	Definition
1.1. Validity	The degree to which an instrument truly measures the construct(s) that wants to measure, free from all possible sources of error or bias.
1.1.1.1. Convergent validity	The extent of the agreement with another (non-criterion) measure that should assess the same behaviour parameter based on face and content validity.
1.1.1.2. Criterion validity	The extent of the correlation between a measure and another already considered as being a criterion or gold standard.
1.2. Reliability	The extent to which an instrument gives consistent, stable, and repeatable measurement. In other words, it is free from measurement error.
1.2.1. Test-Retest	The extent to which test scores are consistent from one test administration to the next, keeping the same conditions (e.g., researcher, timing, preparation, etc.)
1.2.2. Measurement Error	How close the scores on repeated administrations are, expressed in the unit of the questionnaire (i.e., Limits of Agreement (LOA); Standard Error of Measurement (SEM); Smallest Detectable Change (SDC)).
1.2.3. Reliability Coefficients	The proportion of the total variance in the measurements, which is due to consistent differences between subjects (i.e., Intraclass Correlation Coefficient (ICC) and Confidence Intervals (CI) and Cohen’s Kappa coefficient (ordinal measures)).
1.3. Responsiveness	The ability of an instrument to detect change over time in the construct to be measured. Refers to the validity of a change score.

Based on: COSMIN guidelines [21] and Edinburgh Framework [27].

### 2.5 Study risk of bias assessment

The Risk of Bias checklist developed by COSMIN is exclusively for assessing the methodological quality of single studies included in systematic reviews of questionnaires [21]. Given the characteristics of this review, this checklist was adapted. The checklist herein presented has a 4-point scale (i.e., ‘very low risk, ‘low risk’, ‘medium risk’ or ‘high risk’), and contains items on criterion validity, reliability, measurement error and responsiveness. For each measurement property, different design requirements and statistical methods were rated based on the COSMIN standards. Each measurement property was evaluated separately. The overall rating was determined based on “the worst score counts” method as proposed by COSMIN. The criteria for each item can be found in COSMIN guidelines [21]. For reliability, as previously done [19], we defined an ‘adequate’ time interval between test and retest as follows: > 1 day and ≤ 3 months for questionnaires recalling a usual week/month; > 1 day and ≤ 2 weeks for questionnaires recalling the previous week; > 1 day and ≤ 1 week for questionnaires recalling the previous day; > 1 day and ≤ 1 year for questionnaires recalling the previous year.

The data was collected independently by 3 authors (BR, JE and EVC) and disagreements were resolved by discussion with a fourth author (RS).

### 2.6 Effect measures

#### 2.6.1 Quality of measurement properties

To evaluate the studies’ quality of measurement properties we followed the COSMIN guidelines; as such, all measurement properties were rated against quality criteria for good measurement properties [28]. Each result was rated as ‘adequate’ (+), ‘inadequate’ (–), or ‘doubtful’ (?) when design or method was not well reported (e.g., lack of information regarding sample characteristics, lack of information regarding criterion validity).

A study was considered to have ‘adequate’ criterion validity when results for correlations between the questionnaire and the criterion instrument were ≥ 0.70. The accelerometer was considered a criterion measure because, despite that there is no gold standard to measure all movement behaviours, the accelerometer is the only instrument able to do it with proved accuracy and is widely used as criterion comparison measure in validation studies of movement behaviours’ questionnaires [5].

For convergent validity, statistically significant correlations (*p*<0.05) between the movement behaviour and assessments related to the behaviour in question (e.g., between PA and VO_2max_) of ≥ 0.5 and correlations between the movement behaviour measured by similar self-reported instruments of ≥ 0.7 were considered ‘adequate’.

For reliability, Intraclass Correlation Coefficient (ICC) or weighted Kappa ≥ 0.70 were considered ‘adequate’; the use of Pearson or Spearman correlation coefficients was considered ‘inadequate’, because it does not have into account systematic errors [29]. However, Pearson and Spearman correlations > 0.80 were rated positively, similarly to what has been previously done [11].

Measurement error was considered ‘adequate’ when the smallest detectable changes or limits of agreement (LoA) were inferior to minimal important change, and ‘doubtful’ when minimal important change was not defined.

Responsiveness was considered ‘adequate’ when the result was in accordance with the hypothesis or Area Under the Curve (AUC) ≥ 0.70, and ‘doubtful’ when no hypothesis was defined.

For the overall rating of the quality of the studies, if 75% of the results *per* study were ‘adequate’, the overall rating was considered ‘adequate’.

#### 2.6.2 Content validity

Given the characteristics of our search strategy, we did not perform a comprehensive analysis of content validity, but rather applied a subjective reviewers’ rating to assess the content validity of all included questionnaires, as suggested by COSMIN guidelines [21]. In this analysis, several aspects were evaluated as ‘adequate’ (+) or ‘inadequate’ (-), such as: 1) items relevance for the construct, population, and context of use (i.e., the item had to be directed related to the construct or behaviour evaluated); 2) response options and recall period appropriateness for construct, population and context of use (i.e., closed response options were considered inappropriate because they do not capture the movement continuum; the recall period and context had to be clearly stated); 3) comprehensiveness of the construct, population and context of use (i.e., key aspects, such as duration or intensity related to the construct or behaviour had to be clearly stated); and 4) language appropriateness of the response options and items (i.e., clear and simple language).

To evaluate content validity, if the questionnaire was not integrated in the article, we either contacted the authors requesting for the questionnaire or searched online to find it. If access to the questionnaire was not possible, we rated it with ‘cannot be determined´.

### 2.7 Synthesis methods

We conducted a narrative synthesis of the results and organized it in the respective tables (as presented in the results section below).

## 3. Results

### 3.1 Search results

**Fig 1:**
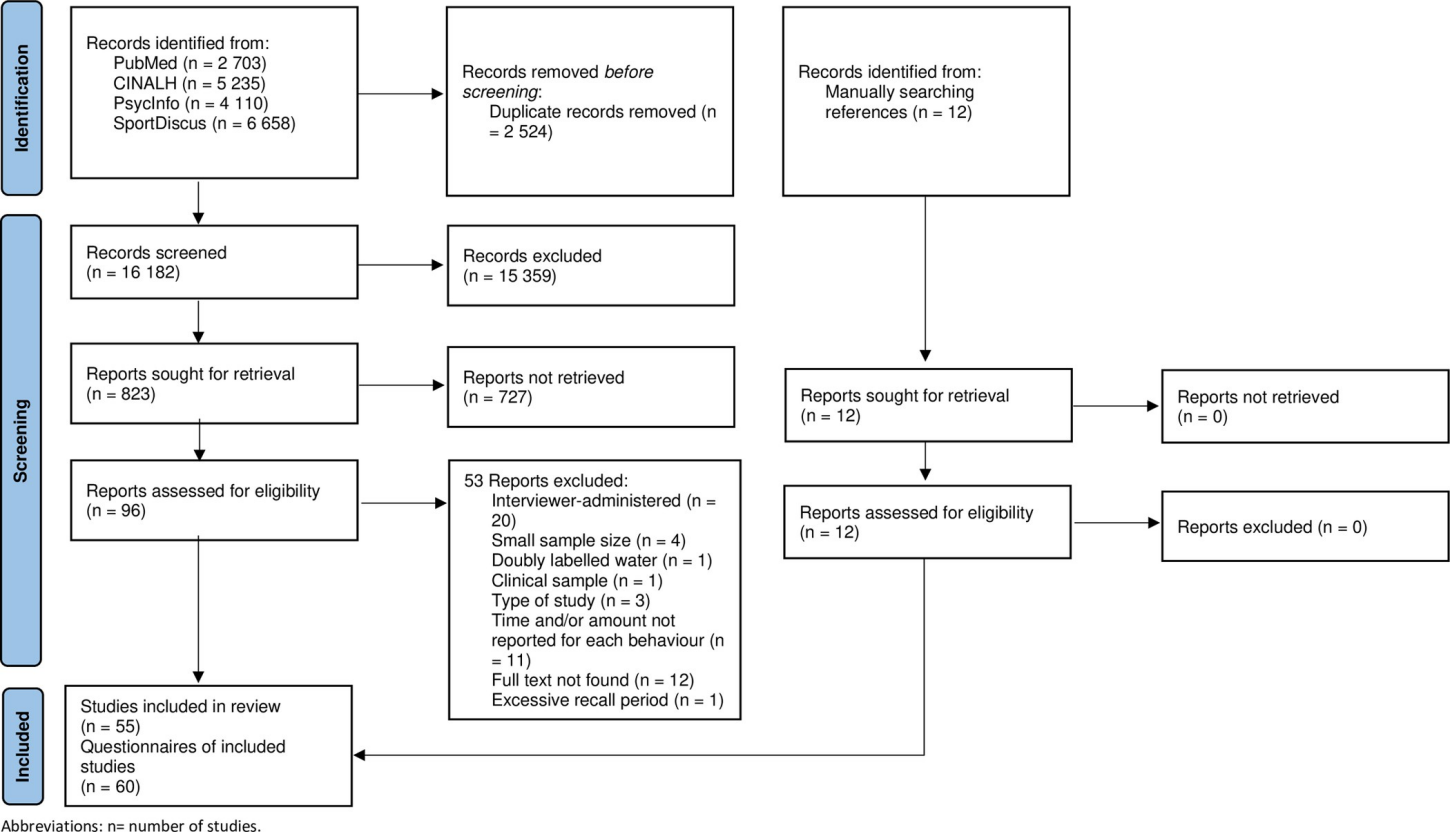
Study selection process flowchart.

The search yield 16,182 articles after removing duplicates. Twelve articles were added after searches in other reviews. Based on titles and abstracts, 108 full texts were selected, and 55 were included, describing 60 questionnaires. The reasons for exclusion of full texts are described in Fig 1.

### 3.2 Results of synthesis

#### 3.2.1 Content description

Twenty-five questionnaires measured PA (17 in adults [30–42], four in older adults [43–46] and four in adults and older adults [47–50]), 12 measured SB (eight in adults [51–58], three in older adults [59, 60] and one in adults and older adults [61]), one measured sleep (in adults and older adults [62]), 12 measured the combination of PA and SB (five in adults [63–67], three in older adults [68–70] and four in adults and older adults [71–74]), one measured the combination of SB and sleep (in adults [75]), and nine measured the combination of PA, SB and sleep (six in adults [67, 76–80], three in adults and older adults [81–83]). There were no proxy-reported questionnaires.

Regarding PA questionnaires [30–50], 68% assessed multi-domain PAs, with leisure-time PA being the most frequent domain (measured in 19 out of 25 PA questionnaires included). The most prevalent response method was the continuous method (68%), focusing on different metrics (e.g., hours/day). The most frequent measurement unit was METs/hour or minute per week or minutes per day (44%). Most of the questionnaires (72%) assessed multiple scores. Recall periods varied from past year (24%), past week (52%), usual week (24%) to currently (12%). None of the questionnaires specified the assessment period (whether a participant is asked regarding a particular type of day, e.g., only weekend days). The number of items included in the PA questionnaires ranged from one to 74.

In the SB questionnaires [51–61], the most prevalent domains were total SB/sitting time (50%) and multi-domain (41.7%). The continuous response method was the most prevalent (66.7%), in hours per day (41.7%) and minutes per day (41.7%). The measurement units depended on the objective of assessment, and the most used score was total SB (91.7%). The most frequent recall periods were past week (33.3%) and usual day (33.3%). Assessment period was specified in 66.7% of the SB questionnaires. The number of items included in the questionnaires ranged from 1 to 20.

There was only one questionnaire assessing sleep duration (Behavioral Risk Factor Surveillance System (BRFSS) Sleep questionnaire) [62]. The response method was continuous, and the measurement unit was hours/day. The recall period was a usual day.

The questionnaires combining PA and SB [63–74], mostly assessed the behaviours through multi-domain (83.3%) with the occupational domain being the most prevalent (11 out of 12 questionnaires). The occupational domain was also used in single domain questionnaires [71, 72]. The most prevalent response method was the continuous method (75%) focusing on different metrics (e.g., hours/week). The most prevalent measurement unit was time (75%) (e.g., hours/week) and several scores were evaluated in all questionnaires, rather than just one score. The most frequent recall periods were usual day/week (66.7%). Assessment period was not specified in 66.7% of the questionnaires. The number of items included in the questionnaires ranged from 3 to 75.

One questionnaire assessed both SB and sleep [75]. This questionnaire had 41 items assessing multi-domain behaviours, the response method was continuous, the measurement unit was hours/day with multi-scores evaluated and the assessment period was specified.

All, except two [79, 81] of the questionnaires measuring a combination of PA, SB, and sleep [67, 76–83] assessed these behaviours through multiple domains. The most prevalent response method was the continuous method (77.8%), focusing on different metrics (e.g., hours/week). The most prevalent measurement units for SB and PA were energy and intensity variables (77.8%) (e.g., METs, kcals) and several scores were evaluated in all questionnaires. For sleep items, the measurement unit was always hours/day. The recall periods focused on the past (55.6%) and in the usual activity (44.4%). Assessment period was not specified in 77.8% of the questionnaires. The number of items included in the questionnaires ranged from 5 to 448. Among these questionnaires, none was designed in terms of content and final scores, to assess all movement behaviours considering the 24h movement behaviour paradigm. The characteristics of the included questionnaires included are presented in Table 2.

**Table 2:** Characteristics of the included movement behaviour questionnaires.

Questionnaire	Age Group, Country	Domains	Response Method	Units of Measurement	Scores	Recall Period/Assessment period	# Items	Parameters
**Physical Activity**								
Nord-Trøndelag Health Study PA Questionnaire (HUNT 1) [29]	Adults, Norway	Leisure	Rating Scale: F: 1–5 (Never-Almost every day); I: 1–3 (without losing breath-near exhaustion); D: 1–4 (<15 min—>60 min)	Weekly physical activity Index (product of F, I and D scales)	F;I; Index	Currently	3	F; D; I
Past Year Total Physical Activity Questionnaire (PYTPAQ) [30]	Adults, Canada	Occupational; Household; Leisure; Transportation	Continuous: hour/day; Days/week	METs hour/week	Total physical activity; Occup physical activity; Household physical activity; Leisure physical activity	Past year	Open table (6 parameters)	F; D; I; M
Physical Activity Assessment Tool (PAAT) [31]	Adults, USA	Total physical activity	Continuous: days/week; min/day	min/week	MPA; VPA; Total physical activity; Active or Inactive	Past week	19	F; D; I
Minnesota Leisure Time Physical Activity Questionnaire (Minnesota LTPA Q) [32]	Adults, USA	Leisure	Continuous: Times/month; hour/Day	METs min/day	Total Leisure time physical activity; Light; MPA; VPA; household chores	Past year	74	D; I
Single Item Physical Activity Measure (SI PA M) [33]	Adults, UK	Total physical activity	Continuous: n° of days	Active days (>30mins)	Number of active days/week	Past week; past month	2 separate questions	F; D
Godin Questionnaire (Godin Q) [32]	Adults, USA	Leisure	Continuous: Times/week	Times/week	Total physical activity; VPA; MPA; LPA; Leisure score	Usual week	4	F; I
CARDIA Physical Activity History (CARDIA) [32]	Adults, USA	Total physical activity	Categorical–yes/no; Continuous: n° months; n° months certain hour/week	hour/month	MPA; VPA; Total physical activity	Past year	3	F; D; I
College Alumnus Questionnaire (College Alumnus Q) [32]	Adults, USA	Leisure; Sport; Transport	Continuous: min/day	METs min/day	EE based on stairs, walking and sports (total index)	Currently, past week; past year	7	F; D; I
Minnesota Heart Health Program Questionnaire (MHHP Q) [32]	Adults, USA	Occupational	?	METs min/day	Work index; leisure index	Currently	6	D; I
Modified Historical Leisure Activity Questionnaire (MHLAQ) [34]	Adults, USA	Leisure; Household and childcare activities; Occupational	Continuous: N° months/year; hour/week	METs hour/week	Total physical activity; MPA; VPA; Leisure physical activity; Household physical activity	Past year	?	F; D; I
Modified version Active Australia Survey 1(MV–AAS1) [35]	Adults, Australia	Leisure	Continuous: hours or minutes; Frequency	METs min/week for categories; min/week for continuous scores; frequency/week	Total physical activity; VPA; MPA; Walking; MVPA; physical activity categories	Past week	?	F; D; I
Modified version Active Australia Survey (MV–AAS2) [36]	Adults, Australia	Leisure	Continuous: hours or minutes; Frequency	min/week and days/week	VPA; MPA; Walking; MVPA; Meet physical activity guidelines	Past week	8	F; D; I
Adapted from Active Australia Survey (Adapt AAS) [37]	Adults, Australia	Leisure; Occupational, Transport; Household	Continuous: hours or minutes; Frequency	min/day	Total physical activity; VPA; MPA; Walking; Meet guidelines	Past week	6	F; D; I
International Physical Activity Questionnaire–Walking Section (IPAQ-WS) [38]	Adults (Australia, Brazil, Finland, Japan, Portugal, Sweden, the Netherlands, UK and USA)	Walking	Continuous: hours or minutes; Frequency	min/week	Walking; Walking + MPA	Past week; Usual week	2	F; D
Short Questionnaire to Assess Health-enhancing physical activity (SQUASH) [39]	Adults, Netherlands	Transport; Leisure; Household; Occupational	Continuous: Days per week; hours and mins per activity/Day; Rating scale for intensity: Slow; Mod; fast or light; MPA; Intense	min/day	Total minutes; Activity score (minutes × intensity)	Usual week	11	F; D; I; M
European Prospective Investigation into Cancer and Nutrition Physical Activity Questionnaire (EPIC PAQ) [40]	Adults, Australia	Occupational; Household; Leisure	Categorical for work: Sedentary occupational–Heavy manual work; Continuous: hour/week	METs hour/week	Total non-occupational physical activity; Recreational physical activity; Household physical activity; VPA (self-rated); VPA (MET assigned); LPA to MPA	Past year, during a usual week in summer and winter	17	F; D; I; M
13-Item Physical Activity Questionnaire (13I-PAQ) [41]	Adults, Czech Republic	Leisure; Occupational; Household	Continuous: hour/day	hour/Week	Total physical activity; Sport physical activity; non-sport LTPA; Occup physical activity; Household	Usual week	13	F; D; M
Questionnaire d’Activité Physique pour les Personnes Âgées (QAPPA) [42]	Older adults, France	Occupational; Household; Leisure; Transportation	Multiple Choice (activities); Continuous: Days/week	METs min/week	Total physical activity; VPA; MPA	Past week	6	F; D; I; M
Incidental and Planned Exercise Questionnaire (IPEQ) [43]	Older adults, Australia	Leisure time, household	Continuous: Times/week; Rating Scale: various	hour/Week	Incidental and planned physical activity	Past week version and Past 3 months version	10 each version	F; D; M
Physical Activity Questionnaire for Elderly Japanese (PAQ-EJ) [44]	Older adults, Japan	Transportation; Exercise/sport; Household; Occupational	Rating Scale: 1–4 (various)	METs hour/week	Total PAQ-EJ; Subtotal Lower intensity categories; Subtotal higher intensity categories	Usual week	14	F; D; M
The Longitudinal Ageing Study Amsterdam Physical Activity Questionnaire (LAPAQ) [45]	Older adults, Nederlands	Walking; transport; household; Leisure; sport	Continuous: Times week; hours and min/past 2 week	METs	METs (> = 6 METs; 3–5.99 METs; 2–2.99); Total physical activity > = 2	Past 2 weeks	18	F; D; M
Nordic Physical Activity Questionnaire (NPAQ-short) [46]	Adults and Older adults, Denmark	Leisure; Transport	Rating scale: 1–5 (< 30 mins—> 5 hours (MVPA) or > 150 min (Vig)); Continuous: hour/week or min/week)	min/week	MVPA; VPA; Compliance with WHO guidelines	Past week	4	D; I
Self-report physical activity questionnaire (SPAQ) [47]	Adults and Older adults, Thailand	Household; Occupational; Leisure; Transport	Continuous: Times/week; Rating scale: 1–6 (0–1 hours—> 9 hour/week)	MET hour/week	Total physical activity; LPA; MPA; household physical activity; occupational physical activity; leisure time recreation; leisure time exercise; transportation	Past week	55	F; D; M
Transport and Physical Activity Questionnaire (TPAQ) [48]	Adults and Older adults, UK	Leisure; Transport	Continuous: number of trips, the total time spent (hours and minutes), the total distance (miles) travelled)	mins/week	Walking for transport; Walking for recreation; Cycling for transport; Cycling for recreation; Moderate Leisure time physical activity, Vigorous Leisure time physical activity, Total physical activity	Past week	16	F; D; M
General Practice Physical Activity Questionnaire (GPPAQ) [49]	Adults and Older adults, UK	Occupational; Leisure	Occup: Categorical about physical activity amount and type. Leisure physical activity: Rating scale 1–4 (None- ≥3 hours). Walking pace: Rating scale 1–4 (Slow-Fast)	hour/week	Activity category (active, moderately active, moderately inactive or inactive)	Past week	7	D
	**Sedentary Behaviour**							
International Physical Activity Questionnaire–Sedentary Behaviour (IPAQ-SB) [50]	Adults, UK, USA, Netherlands	Total sedentary behaviour	Continuous: hours and minutes	hour/Day	Total Sitting time	Past week, on weekdays and weekend days	2	D
Australian Longitudinal Study on Women’s Health—Sedentary Behaviour Questions (ALSWH—SB Q) [51]	Adults, Australia	Transport; Occupational; Watching TV; Using computer at home; Leisure	Continuous: hours and minutes	hours and min/day	Transport; Occupational; Watching TV; Using computer at home; Leisure time	Currently, on weekdays and weekend days	5	D
Self-reported sitting and breaks from sitting in the workplace (SBSW) [52]	Adults, Australia	Occupational	Continuous: hours and/or minutes; Frequency breaks per hour	min/day for sitting; Frequency/hour work breaks	Sitting; breaks from sitting	Past week, on workdays	2	F; D
Workplace Sitting Breaks Questionnaire (SITBRQ) [53]	Adults, Australia	Occupational	Rating scale: n° of breaks; hours in breaks per day	Breaks/hour; min/day	Frequency of breaks; Total duration of breaks	Usual workday	2	F; D
Sedentary Behaviour Questionnaire (SBQ) [54]	Adults, USA	TV; Computer games; sitting listen to music, sitting talking on telephone; office/paperwork; Reading; Play musical instrument; Arts and crafts; sitting driving a car.	Rating Scale: 0–6; (None—> 6 hours)	hour/week; hour/day	TV; Computer games; sitting listen to music, sitting talking on telephone; office/paperwork; Reading; Play musical instrument; Arts and crafts; sitting driving a car. Total hour/week; total hour/weekday; total hour/weekend	Usual weekday; Usual weekend day	18	D; M
SED-GIH [55]	Adults, Sweden	Total sitting time	Rating scale: 1–7 (Never- Virtually all day)	Categorical: hour/day	Total sitting time	Usual day	1	Duration
Workforce Sitting Questionnaire (WSQ) [56]	Adults, Australia	Transport; Occupational, TV; Computer; Leisure	Continuous: min/day	min/Day	At work, workday; Total, all domains, workday; Total, all domains, non-workday; Average total, work and non-workdays	Past week, on workdays, and non-workdays	?	D; M
Japanese-Language Self-reported Measures for Assessing Adults Domain-Specific Sedentary Time (JSRM—SB) [57]	Adults, Japan	Transport, Occupational; TV; Computer use; Other leisure time	Continuous: min/day	min/day	Total Sitting time at workday, non-workday, and whole week	Past week, on workdays and non-workdays	6	D; M
Longitudinal Aging Study Amsterdam questionnaire (LASA) [58]	Older adults, Netherlands	Leisure; Occupational; Transport; Household; TV	Continuous: hours and min/day	hour/day	Total sitting time	Usual week and weekend day	20	D; M
SB question of the Yale Physical Activity Survey (YPAS—SB) [59]	Older adults, USA	Total sedentary behaviour	Continuous: hour/day	hour/day	Total sitting time	Usual day	1	D
Community Health Activities Model Program for Seniors SB questions (CHAMPS–SB) [59]	Older adults, USA	Total sedentary behaviour	Continuous: hour/week	hour/week	Total sitting time	Usual week	9	D; F
Cancer Prevention Study-3 Sedentary Time Survey (CPS-3 sitting time) [60]	Adults and older adults, USA	Total sitting time	Rating scale 1–8 (0-+11 hours)	hour/Day	Total sitting time	Past year, on weekdays and weekend days	4	D; M
**Sleep**								
BRFSS sleep questions (BRFSS Sleep) [61]	Adults and older adults, USA	Duration	Continuous: hour/day	hour/Day	Total sleep time	Usual day	1	D
	**Physical Activity + Sedentary Behaviour**							
Kaiser Physical Activity Survey (KPAS) [62]	Adults, USA	Leisure; Occupational; Household; Transport, TV	Rating scale 1–5 (caregiving section: 1–4 (never -always; Other sections: 1 to 5(none- >20 hour/week). various options)	Activity score; Frequency and duration for the three most frequent sports/exercise	Caregiving; Housework; Housework/caregiving; Sports/exercise; Active living habits; Occupational; 3-point or 4-point summary	Past year	75	F; D
Sedentary, Transportation and Activity Questionnaire (STAQ) [63]	Adults, France	Occupational, Transport, Leisure	Continuous: hour/week or day/week. Categorical (various options)	hour/week or day/week	Total, work, transport, leisure; leisure sedentary behaviour: total, screen time, reading, writing, listening to music, sewing; Transport: Active, passive	Past month on workdays and non-workdays	56	D; F; M
International Physical Activity Questionnaire (IPAQ) [64]	Adults, Australia, Brazil, Canada, Finland, Guatemala, Netherlands, Japan, Portugal, South Africa, Sweden, United States, San Diego, United States, South Carolina, United Kingdom	Long form: Occupational, Transport, Household, Garden, Leisure, Sitting time. Short Form: Total physical activity, Sitting time	Continuous: Frequency, hours and minutes	MET-min/week for physical activity; min/week for Sitting time; Guideline’s compliance (yes or no)	Total physical activity; Total sitting time; Guideline compliance	Past 7 days or usual week on weekdays and weekend days	Short Form 7; Long Form 27	F; D; I; M
Australian Women’s Activity Survey (AWAS) [65]	Adults, Australia	Planned activities, employment, childcare, domestic responsibilities, and transportation	Dichotomic: yes/no; Continuous: hours, days, min)	min/week	Sitting; LPA; MPA; VPA; Total activity	Usual week on the past month	72	F; D; I
Workers’ sitting- and walking-time questionnaire Time Method (WSWQ- t-method) [66]	Adults, Japan	Occupational; Leisure	Continuous: hours and mins	hours and mins	Sitting and walking/standing during working time and non-working time; sitting and walking/standing during non-workday	Usual day on the past month	6	D
The Physical Activity Scale for the Elderly (PASE) [67]	Older adults, UK	Occupational; Household; Leisure; Sports	Rating scale: 1–4 never—often; less than 1h - more than 4h	METs Hour/day	PASE activity score	Past week	23	F; D; I; M
Community Health Activities Model Program for Seniors physical activity self-report questionnaire + transport items (CHAMPS+transport) [68]	Older adults, USA	Leisure; Occupational; Household; Transportation; Watching TV	Continuous:	times/week; METs hour/week	Sedentary time; low-LPA, high-LPA; MVPA; Total physical activity	Usual week on the past month	52	F; D; I; M
Community Healthy Activities Model Program for Seniors (CHAMPS) [69]	Older adults, USA	Leisure; occupational; household; transportation, watching TV	Continuous: Times/week; hour/week	times/week; METs hour/week	Caloric expenditure per week in at least moderate intensity physical activities; frequency per week in at least moderate intensity physical activities; Caloric expenditure per week in all listed physical activities; Frequency per week in all listed physical activities	Usual week on the past month	41	F; D; I; M
Modified Version of the MONICA Optional Study on Physical Activity Questionnaire (Modified MOSPA-Q) [70]	Adults and older adults, Australia	Occupational	Continuous: hours and mins	min/workday	Lifting/Carrying; Standing; Sitting; Walking	Usual workday on the past week	4	D; M
Occupational Sitting and Physical Activity Questionnaire (OSPAQ) [70, 71]	Adults and older adults, Australia	Occupational	Continuous: hours and minutes; Percentage	min/workday	Heavy labour; Standing; Sitting; Walking	Usual workday on the last 7 days	6	D; M
Rapid Assessment Disuse Index (RADI) [72]	Adults and older adults, USA	Total physical activity and sitting time	Rating scale 1–5 (number of hour/day)	Index based on hour/day or number of flights	Moving; number of flights of stairs; Sitting time; RADI scores	Usual day on the past week/month/year	3	D
Global Physical Activity Questionnaire (GPAQ) [73]	Adults and Older adults, Japan	Occupational; transportation; Leisure	Dichotomic: yes/no; Continuous: hours, days, mins	min/week; hour/week	Work Domain: Sedentary, Vigorous intensity, Moderate intensity, Total; Transport: walking and cycling, Total; Leisure Domain: Sedentary, Vigorous intensity, Moderate intensity, Total	Usual day, typical week	19	F; D; I
	**Sedentary Behaviour + Sleep**							
SIT-Q [74]	Adults, Australia	Sleep; Napping; Transportation; Occupational; Household; Leisure	Continuous: hour/day	hour/day	Meals; Transportation; Occupational; Child and elder care; TV; computer use; Leisure; Total sitting time	Past year in usual day, on weekdays and weekend day	41	D
	**Physical Activity + Sedentary Behaviour + Sleep**							
Physical Activity Questionnaire (PAQ) [75]	Adults, Sweden	Occupational; Leisure; Housework; Sitting/TV/reading; Walking/bicycling; Sleep	Rating scale 1–5 (Various); Sleep (continuous: hour/day)	METs-hour/day	Crude Total physical activity; Total activity score; Work/occupation; Home/household work; Active leisure-time (walking/bicycling + exercise); Inactive leisure-time (TV/reading); Sleeping	Past year, on the usual day	5	F; D; I
Athens Physical Activity Questionnaire (APAQ) [76]	Adults, Greece	Occupational physical activity; Recreational physical activity; Home activities; Sleep; sedentary behaviour	Continuous: Times/week; hour/day; min/day	MJ/day	Occupational physical activity; recreational physical activity; home activities	Past week	23	F; D
Sedentary Time and Activity Reporting Questionnaire (STAR-Q) [77]	Adults, USA	Eating; Personal/medical care; Sleep; Occupation; Transportation; Household; Yard work; Caregiving; Exercise; Light leisure; Stair-climbing; and “other” activities	Continuous: hour and min/day; number of times	For energy expenditure: Kcal/Day; For intensities: METs Hour/day	TEE, kcal/day; AEE, kcal/day; AEE, kcal/kg.day; Sleeping; Stair-climbing, flights/day; Active sitting; Overall activity (SB; LPA; MPA; VPA); Exercise, sports, and leisure activity (light, mod, vig); Occupational activity (sitting; sedentary behaviour; Light; Mod)	Past month	448	F; D; I; M
Question 8 of the Paffenbarger Physical Activity Questionnaire (Q 8 PPAQ) [78]	Adults, USA	Total physical activity, sleep and sedentary behaviour	Continuous: hour/day	METs Hour/week; hour/day	Time spent sleeping or reclining, participating in sitting activities, and engaging in light (< 3 METs), MPA (3–6 METs), VPA (> 6 METs)	Usual day, on weekdays and weekend days	5	D; I
EPIC-Norfolk Physical Activity Questionnaire (EPAQ2) [79]	Adults, UK	Leisure; Sport; Occupational; household; Sleep duration	TV: 1–6 (none->4h day); Stair climbing; 1–6 (None->20 times/day); Household physical activity: 1–7 (None->15 hour/week); Occup physical activity and sedentary behaviour- Continuous: hour/week; Stairs at work: 1–6 (none->20 times/day; Kneeling and squatting: Dichotomous (kneeling and squatting > 1 hour; get up>30 times); Leisure physical activity: 1–8 (none- > 6 times/week) and continuous: hours and mins per episode); Sleep- Continuous: hour/day	hour/week; METs hour/week	TV time; Activity at work; Activity at home; Recreational activity; VPA; physical activity index	Past year, on weekdays and weekends days	87	F; D; M
Workers’ sitting- and walking-time questionnaire Percentage Method (WSWQ—p-method) [66]	Adults, Japan	Occupational; Leisure; Sleep duration	Continuous: hours and min/day	Proportion of time (%)	Sitting and walking/standing during working time and non-working time; sitting and walking/standing during non-workday	Usual day on the last month	14	F; D
New Questionnaire on Physical Activity (NQPA) [80]	Adults and older adults, Netherlands	Total physical activity, sedentary behaviour, and sleep	Continuous: hours per day, week, or month	KJ/day	Rest; Occupational; Leisure Time	Past year	28	F; D; I
Web-Based Physical Activity Questionnaire (Active-Q) [81]	Adults and Older adults, Sweden	Leisure; Transportation; Occupational; Sport; Sleep	Transport–Rating scale: 1–5 (>15 min—1–2 hours). Leisure activities–Rating scale: 1–6 (<30 mins—>8 hours. Sport—Rating scale: 1–5 (1–3 times/week—5–7 times/week); Rating scale: 1–5 (<30 mins—2–4 hours). Sleep: Rating scale: 1–6 (<5 hours—≥10 hours)	min/day	sedentary behaviour; LPA; sedentary behaviour + LPA; MPA; VPA; MVPA	Usual activity on the last months	47	F; D; M
Flemish Physical Activity Computerized Questionnaire (FPACQ) [82]	Employed/unemployed adults, Belgium	Occupation; Transportation in leisure time; Watching television or video and playing computer games; Home and garden activities; Sleeping, MPA; VPA in leisure time; Sports participation	Continuous: hours per day or week; times per week or year; Kgs; Multiple choice	Kcal/Week: energy expenditure variables; hour/week: time variables	Time/week spent on sports participation; Energy expenditure/week on sports participation; Average energy expenditure on sports participation; Time/week spent eating; Time/week spent sleeping; Time/week spent watching television or videos or playing computer games; Time/week spent on leisure-time active transportation; Time/week spent on active leisure-time activities; Energy expenditure/week on active leisure time-activities; Average energy expenditure on active leisure-time activities; Time/week spent on occupation and transportation to and from occupation; Energy expenditure/week on occupation and transportation to and from occupation; Average energy expenditure on occupation and transportation to and from occupation; Overall energy expenditure during a usual week; physical activity level	Usual week	19	F; D
	Retired older adults, Belgium	Transportation in leisure time; Watching television or video and playing computer games; Home and garden activities; Sleeping; MPA and VPA in leisure time; sports participation					12	F; D

Abbreviations: n = Sample Number; SD = Standard Deviation; F = Frequency; D = Duration; I = Intensity; M = Mode; NA = Not Applicable; UK = United Kingdom; USA; United States of America; min = Minutes; MPA = Moderate Physical Activity; MVPA = Moderate to Vigorous Physical Activity; VPA = Vigorous physical activity; LPA = Light Physical Activity; TEE = Total Energy Expenditure; Kcal = Kilocalories.

#### 3.2.2 Content validity

Table 3 presents the summary of the content validity results and its details are provided in a supporting file [see S1 Table]. Most of the questionnaires (68.3%) showed ‘adequate’ content validity.

**Table 3:** Summary of results.

Questionnaire	Validity Quality		Reliability Quality	Measurement Error Quality	Content Validity Quality	Risk of bias			
	Criterion	Convergent				Validity		Reliability	Measurement Error
						Criterion	Convergent		
**Physical Activity**									
HUNT 1 [29]	-	-/-	+		+	1	1/1	3	N.R.
PYTPAQ [30]	-	-	-		+	1	1	1	N.R.
PAAT [31]	-	-	-		+	1	1	3	N.R.
Minnesota LTPA Q [32]	-	-/-	+		+	1	1/1	3	N.R.
SI PA M [33]	NA	-/-	-/+		-	NA	2/2	3	N.R.
Godin Q [32]	-	-/+	-		+	1	1/1	4	N.R.
CARDIA [32]	-	-/-	+		+	1	1/1	3	N.R.
College Alumnus Q [32]	-	-/-	-		-	1	1/1	3	N.R.
MHHP Q [32]	-	-/-	+		CD	1	1/1	3	N.R.
MHLAQ [34]	NA	-	+		CD	NA	1	2	N.R.
MV–AAS1 [35]	-	NA	-	?	CD	4	NA	4	2 (continuous scores, not reported)
MV–AAS2 [36]	-	NA	-	?	+	4	NA	4	4
Adapt AAS [37]	-	NA	-	?	+	4	NA	2	2 (continuous scores, not reported)
IPAQ-WS [38]	-	NA	-		+	1	NA	4	N.R.
SQUASH [39]	-	NA	-		+	1	NA	3	N.R.
EPIC PAQ [40]	-	-	-	?	+	4	1	3	1
13I-PAQ [41]	NA	-/-/-	+		+	NA	2/2/2	2	N.R.
QAPPA [42]	NA	-/-/-	-	?	+	NA	4/4/4	4	4
IPEQ [43]	NA	-	+		-	NA	4/4/4/4	2	N.R.
PAQ-EJ [44]	-	NA	-		+	4	NA	3	N.R.
LAPAQ [45]	-	NA	-	?	+	1	NA	3	1
NPAQ-short [46]	-	NA	-		+	1	NA	3	N.R.
SPAQ [47]	-	NA	+		-	1	NA	3	N.R.
TPAQ [48]	-	NA	-	?	-	1	NA	2	2
GPPAQ [49]	CD	NA	-	?	-	4	NA	4	4
**Sedentary Behaviour**									
IPAQ-SB [50]	-/-	NA	-/+		+	1	NA	3	N.R.
ALSWH—SB Q [51]	NA	-	-	?	+	NA	1	1	2
SBSW [52]	-	NA	-		+	1	NA	2	N.R.
SITBRQ [53]	-	NA	-	?	+	NA	NA	3	2
SBQ [54]	-	-	+		-	1	1	2	N.R.
SED-GIH [55]	-	NA	+		-	1	NA	4	N.R.
WSQ [56]	-	NA	-		CD	1	NA	1	N.R.
JSRM—SB [57]	-	NA	-		CD	1	NA	1	N.R.
LASA [58]	-	NA	+		+	1	NA	1	N.R.
YPAS–SB [59]	-	NA	-		+	1	NA	1	N.R.
CHAMPS–SB [59]	-	NA	-		+	1	NA	1	N.R.
CPS-3 ST [60]	-	NA	-		-	1	NA	3	N.R.
**Sleep**									
BRFSS Sleep [61]	CD	NA	CD		+	4	NA	4	N.R.
**Physical Activity + Sedentary Behaviour**									
KPAS [62]	-	-/-	+		-	1	1	1	N.R.
STAQ [63]	-	-	-	?	+	1	1	1	2
IPAQ [64]	-/-/-/-	+/+	+/+/+/+	?	+	1	1	4	2 (continuous scores, not reported)
AWAS [65]	-	NA	-	?	+	1	NA	1	2
WSWQ- t-method [66]	-	NA	-		+	1	NA	1	N.R.
PASE [67]	NA	-/-/-/-	+		-	NA	1/1/1/1	4	N.R.
CHAMPS+transport [68]	-	NA	-		+	1	NA	4	N.R.
CHAMPS [69]	NA	-/-	-		+	NA	1/1	4	N.R.
Modified MOSPA-Q [70]	-	NA	-		+	1	NA	4	N.R.
OSPAQ [70, 71]	-/NA	NA/-	+/-		+	1/NA	NA/1	4/2	N.R./N.R.
RADI [72]	-	NA	-		-	1	NA	3	N.R.
GPAQ [73]	-	-	+		+	1	1	3	N.R.
**Sedentary Behaviour + Sleep**									
SIT-Q [74]	NA	-	-	?	+	NA	1	1	1
**Physical Activity + Sedentary Behaviour + Sleep**									
PAQ [75]	NA	-	-		-	NA	1	3	N.R.
APAQ [76]	+	NA	+	?	+	1	NA	2	2
STAR-Q [77]	NA	-	-		+	NA	1	1	N.R.
Q 8 PPAQ [78]	-	NA	-	?	+	1	NA	1	1
EPAQ2 [79]	-	-	-	?	-	1	1	3	4 (continuous scores, not reported)
WSWQ—p-method [66]	-	NA	+		+	1	NA	1	N.R.
NQPA [80]	NA	-	-	?	CD	NA	1	3	2
Active-Q [81]	-	NA	-		-	1	NA	1	N.R.
FPACQ [82]	-	NA	+	?	-	1	NA	1	2

Abbreviations:— = Inadequate; + = Adequate;? = Doubtful; NA = Not applicable; CD = Cannot be determined; N.R. = Not reported; 1 = Very low risk of bias; 2 = Low risk of bias; 3 = Medium risk of bias; 4 = High risk of bias.

Regarding PA questionnaires, only three were considered ‘inadequate’ (two in adults [33, 34] and one in adults and older adults [43]). Three questionnaires (in adults) [33, 35, 36] were not available, therefore, their content validity could not be determined.

For SB, three questionnaires (two in adults [55, 56] and one in adults and older adults [61]) were considered to have inadequate content validity. One questionnaire [57] was not assessed as its content was not available.

The sleep questionnaire was considered to have ‘adequate’ content validity. For PA and SB, three questionnaires were considered to have ‘inadequate’ content validity (one in adults [63], one in older adults [68] and one in both [73]).

The SB and sleep questionnaire [75] was considered with adequate content validity.

For PA, SB and sleep questionnaires 4 questionnaires were considered ‘inadequate’ (two in adults [76, 80] and two in adults and older adults [82]. One questionnaires [81] (in adults and older adults) was not available, therefore, their content validity could not be determined.

The main reason for the content validity inadequacy was the response options not being appropriate (i.e., closed response, rating scales).

#### 3.2.3 Validity

Table 3 presents the summary of the results for validity, and its details are provided in a supporting file [see S2 Table]. Only the Athens Physical Activity Questionnaire (APAQ) [77] had ‘adequate’ overall quality for criterion validity and the International Physical Activity Questionnaire (IPAQ) [65] had ‘adequate’ overall quality for convergent validity. Overall, 36.7% of the studies did not specify the sample characteristics. The most frequently calculated coefficients were Pearson and Spearman correlations, Kappa’s coefficients, percentages of agreement and intraclass correlation coefficients. Bland and Altman statistics examined measurements of precision in 30% of the questionnaires.

In the PA questionnaires, none of the questionnaires showed overall ‘adequate’ criterion or convergent validity. Criterion validity was assessed with accelerometery in 76% of the questionnaires; however, the accelerometer protocols used (e.g., epoch length, valid day definition) varied substantially between studies. The best results with accelerometery were regarded Self-Report Physical Activity Questionnaire (SPAQ) light, moderate and household PA scores [48], and Transport and Physical Activity Questionnaire (TPAQ) vigorous PA score [49]. Some questionnaires [34, 35, 42–44] only assessed convergent validity and these were performed against other subjective measures or variables related to PA behaviour (e.g., VO_2max_, body fat). The CARDIA Physical Activity History (CARDIA) [33], Minnesota Heart Health Program Questionnaire (MHHP Q) [33], 13-Item Physical Activity Questionnaire (13I-PAQ) [42] and Incidental and Planned Exercise Questionnaire (IPEQ) [44] questionnaires were the ones showing the best convergent validity in some scores.

Regarding SB questionnaires, none showed overall ‘adequate’ criterion or convergent validity. The accelerometer was the criterion measure in 91.7% of the questionnaires. The Australian Longitudinal Study on Women’s Health—Sedentary Behaviour Questions (ALSWH-SB Q) [52] showed the best convergent validity scores; nevertheless, these only took into account computer use (*r* = 0.74) and occupational SB (ICC = 0.77).

Regarding sleep, the BRFSS Sleep questionnaire [62] was evaluated against criterion and convergent measures; however, its validity quality could not be determined given that only Bland and Altman statistics were performed.

For the questionnaires combining PA and SB, none showed overall ‘adequate’ criterion validity. For these questionnaires, the accelerometer was the criterion measure in 75% of the questionnaires. Concerning criterion validity, the Sedentary, Transportation and Activity Questionnaire (STAQ) [64] questionnaire showed the best performance regarding the sitting time at work score (ICC = 0.82), when evaluated against accelerometery. The IPAQ‘s short form, past and usual week versions, were rated with an ‘adequate’ overall convergent validity, when compared to the respective long forms [65].

The SIT-Q [75] was the only questionnaire combining SB and sleep and was evaluated against one convergent measure (e.g., Seven-Day Activity Diary). In this questionnaire, occupational SB was the only score with ‘adequate’ convergent validity (*rho* = 0.75).

Concerning the questionnaires combining all movement behaviours, the APAQ [77] showed ‘adequate’ overall criterion validity against accelerometery for total energy expenditure (*rho* = 0.84). The Sedentary Time and Activity Reporting Questionnaire (STAR-Q) [78] showed the best performance for convergent validity; this was assessed against a 7-day activity diary (energy expenditure *rho* = 0.74; general occupational activity *rho* = 0.71; occupational sitting *rho* = 0.75; and SB *rho* = 0.75).

#### 3.2.4 Reliability and measurement error

Table 3 presents the summary of the results of the reliability and its details are provided in a supporting file [see S3 Table]. ‘Adequate’ overall reliability quality was observed in 37% of the questionnaires: seven PA questionnaires [30, 33, 42, 44, 48], four SB questionnaires [51, 55, 56, 59], eight questionnaires combining PA and SB [57, 63, 65, 68, 74], and three questionnaires combining PA, SB and sleep [67, 77, 83]. Sample characteristics for the reliability results were not specified in 42% of the studies. The time between test and retest ranged between two days to one year. The most often used statistical approaches to assess reliability were Pearson and Spearman correlations, ICCs, Kappa’s coefficients and percentages of agreement.

For measurement error, Bland and Altman plots comparing test and retest were applied in 31.7% of the questionnaires. Measurement error was calculated in 19 (out of 60) questionnaires and all were rated with ‘doubtful’ overall measurement error quality, because minimal important change was not reported (PA: four in adults [36–38, 41], two in older adults [43, 46] and two in both [49, 50]; SB: two in adults [52, 54]; PA and SB: three in adults [64–66]; SB and sleep: one in adults [75]; and PA, SB and sleep: three in adults [77, 79, 80]).

#### 3.2.5 Responsiveness

The details on responsiveness are provided in a supporting file [see S4 Table]. Only one study (Community Healthy Activities Model Program for Seniors; CHAMPS) [69] evaluated responsiveness. The measures had small to moderate effect sizes (0.38 to 0.64), which resulted in an ‘adequate’ overall responsiveness quality, given that the results of the study were in accordance its hypothesis.

### 3.3 Risk of bias

Table 3 presents the summary of the results of risk of bias and its details are provided in a supporting file [see S5 Table]. The overall rating for risk of bias regarding criterion validity was very low for 72.2% of the studies. The main cause for high the risk of bias was the absence of sensitivity and specificity of dichotomous scores. For the overall reliability risk of bias, 23.6% of the studies were rated with a very low risk of bias. For the overall rating of measurement error risk of bias, 21.1% of the studies were classified with very low risk of bias. The main reasons for high the risk of bias for reliability or measurement error were the inappropriate interval between test and retest and the statistical methods used (e.g., correlations instead intra class correlations). For convergent validity, 82.4% of the studies were classified as having an overall very low risk of bias. The only study assessing responsiveness was rated with a medium risk of bias for this measurement property (CHAMPS) [70].

## 4. Discussion

This systematic review identified and described questionnaires assessing sleep, SB and PA or the combination of these movement behaviours, in adults and older adults.

We identified 60 questionnaires, describing content and measurement properties. Of these, 25 questionnaires measured PA, 12 SB, one sleep, 12 the combination of PA and SB, one the combination of SB and sleep, and nine the combination of PA, SB, and sleep. Results showed high heterogeneity in the questionnaires’ content, measurement properties and quality, which precluded a meta-analysis. Indeed, the questionnaires’ content varied substantially in terms of behaviour’s domain assessed, response method, measurement units, scores, recall and assessment periods, as well as, in the number of items and parameters evaluated.

The validity of the included questionnaires was mostly assessed by comparing the questionnaire with accelerometers, and the quality of validity results was frequently ‘inadequate’. This could potentially be due to desirability bias or cognitive issues related to recall or comprehension of the questionnaires [9].

Only one questionnaire (APAQ) [77] measuring the combination of sleep, SB and PA showed ‘adequate’ overall quality for criterion validity. However, the validation results were only for total energy expenditure, which requires careful interpretation, because this outcome poses some limitations; as, the energy expenditure depends on other factors rather than movement behaviours (e.g., resting energy expenditure and thermic effect of food) [6]; accelerometery is not the most appropriate criterion measure to assess energy expenditure [6]; we cannot determine the results for each behaviour; and this is not a time-focused variable. In this sense, to assess a given movement behaviour, the actual time spent in it, seems to be a better output, which is the output generated by accelerometery. Although the limitations of accelerometery are well known, this still seems to be one of the best objective criterion measures to assess time spent in movement behaviours, in free living conditions [84]. Likewise, devices combining heart rate monitoring and accelerometery technologies to assess the intensity and time spent in different movement behaviours [6, 85] might also be adequate options for validation studies.

Regarding the reliability of the included questionnaires, there were different intervals between test and retest and the overall results’ quality was also frequently ‘inadequate’. However, these results are dependent on the number of scores that authors evaluated. For example, the PASE [68] was rated with an overall ‘adequate’ reliability; however, the authors only assessed the reliability for a single score; whereas in more complex questionnaires (i.e., with more scores), such as the WSQ [57], that presented ‘adequate’ reliability result in a general score (i.e., total, all domains ICC = 0.80), the overall quality was considered ‘inadequate’, due to the separated scores for reliability. Furthermore, the statistical procedures used by the different studies were often considered ‘inadequate’, mainly because Pearson or Spearman correlations were used instead of ICCs or Kappas, or because the time interval between test and retest was inappropriate. Indeed, despite Pearson and Spearman correlations do not have into account for systematic errors [29], these have been widely used in validity and reliability studies; however, it is well known that for continuous scores, ICCs are considered more appropriate, while for categorical scores, Kappas are advised [86]. For absolute validity by means and limits of agreement, Bland and Altman plots are recommended [87]; however, these were calculated only in a few of the included studies, either to report on validity or on reliability. Our findings largely contradict the conclusions of the studies included in this review, which considered that the questionnaire under study was valid and reliable, given that these studies used other metrics instead of the COSMIN quality criteria.

IPAQ [65] showed at simultaneously ‘adequate’ reliability and convergent validity, but not for criterion validity. For a questionnaire have an adequate validation, at least ‘adequate’ overall validity and reliability need to be attained, and a criterion measure is better than a convergent one to that purpose [21].

Responsiveness was only tested for CHAMPS [70]. Other reviews have also reported a lack of responsiveness assessment of questionnaires measuring PA [11, 19]. However, assessing questionnaire’s responsiveness is paramount to understand whether they are capable of measuring changes in movement behaviours over time [25].

Many questionnaires showed a high variability in content, together with inadequate measurement properties, which highlights the complexity of assessing the full spectrum of movement behaviours across the 24h period and reinforces the need for better self-reported questionnaires to measure movement behaviours combinations. The emergence of the 24h movement guidelines, due to its specific characteristics, raises the need to adapt or develop *de novo* instruments to assess 24h movement behaviours. The same concern has been raised regarding the new WHO PA and SB guidelines for adults [88].

The lack of questionnaires assessing 24h movement behaviours in an integrated way precludes accurate report of 24h movement behaviour guidelines’ compliance and trends over time [89, 90], increases the risk of misclassification, and of biased and unreliable results [10]. Moreover, whilst new guidelines are developed and public health efforts to increase PA and decrease sedentary time proceed, measurement instruments should be improved; surveillance systems are adapted, and broadly and repeatedly implemented [91]. Indeed, measuring movement behaviours is complex and there is a need for better solutions, mainly to assess all movement behaviours in an integrated fashion. Given the measurement properties and the content of the questionnaires assessing a combination of all movement behaviours herein presented, there seems to be no single questionnaire capable to accurately measure these behaviours, considering the new 24h movement paradigm.

### 4.1 Limitations and strengths

We systematically reviewed existing questionnaires that measure all movement behaviours together or isolated, in adults and older adults. Comparing questionnaires’ measurement properties is complex, given the heterogeneity of the data, including different scores, domains, variety of recall periods, comparison measures and reporting units. For example, the studies using accelerometery data to assess questionnaires’ validity applied different epoch lengths, different definitions of (non)wear time and different placement sites. These aspects make comparisons between studies very difficult. Although the use of COSMIN guidelines should be considered a strength of this review, the COSMIN cut points to evaluate the quality of measurement properties may somewhat lead to loss of information, due to the mechanistic way of analysing data. Also, to the best of our knowledge, this review contains the largest sample of data/questionnaires assessing movement behaviours.

## 5. Conclusions

We systematically reviewed existing questionnaires that measure sleep, SB or PA, or their combination, in adults and older adults. There are several questionnaires with different characteristics and outputs for all movement behaviours. The included questionnaires presented frequent methodologic limitations, that resulted in inadequate validity and reliability scores. Existing questionnaires have insufficient measurement properties, and none was developed considering the 24h movement behaviour paradigm. The lack of valid and reliable questionnaires assessing 24h movement behaviours in an integrated way, precludes accurate monitoring and surveillance systems of 24h movement behaviours.

## Supporting information

S1 Checklist PRISMA 2020 checklist. (DOCX)

S1 File Search strategy. (DOCX)

S2 File Extracted information. (DOCX)

S1 Table Content validity table. (DOCX)

S2 Table Validity results. (DOCX)

S3 Table Reliability results. (DOCX)

S4 Table Responsiveness. (DOCX)

S5 Table Risk of bias. (DOCX)
