# Supplementary material for: Questionnaires measuring movement behaviours in adults and older adults: Content description and measurement properties. A systematic review
Source: PLoS One. 2022 Mar 11;17(3):e0265100. doi: 10.1371/journal.pone.0265100 (PMC8916622; doi:10.1371/journal.pone.0265100)
Supplement: S1 File — (DOCX) [file pone.0265100.s002.docx]

**Supporting file 1 – Search Strategy**

**PubMed**

(exercise[Mesh] OR exercise [Title/Abstract] OR "physical activity" [Title/Abstract] OR "physical activities"[Title/Abstract] OR sedentary behavior"[Mesh] OR "sedentary behavior [Title/Abstract] OR "sedentary behaviors" [Title/Abstract] OR "sedentary behaviour" [Title/Abstract] OR "sedentary behaviours" [Title/Abstract] OR "sitting position" [Mesh] OR "screen time" [Mesh] OR sedentar* [Title/Abstract] OR sleep [Mesh] OR sleep [Title/Abstract] OR "movement behavio*" [Title/Abstract] OR "movement behaviou*" [Title/Abstract] OR inactiv* [Title/Abstract])

AND

(surveys OR survey OR surveillance OR questionnaire OR questionnaires OR "proxy-reported" OR "proxy-report" OR "proxy-reports" OR "proxy report" OR "proxy reported" OR instrument OR instruments OR measur* OR scale OR scales OR assess* OR tool OR tools OR Interview* OR "self-report" OR "self-reported" OR "self-reports" OR "self report" OR "self reported" [Title/Abstract])

AND

(instrumentation[sh] OR methods[sh] OR “Validation Studies”[pt] OR “Comparative Study”[pt] OR “psychometrics”[MeSH] OR psychometr*[tiab] OR clinimetr*[tw] OR clinometr*[tw] OR “outcome assessment (health care)”[MeSH] OR “outcome assessment”[tiab] OR “outcome measure*”[tw] OR “observer variation”[MeSH] OR “observer variation”[tiab] OR “Health Status Indicators”[Mesh] OR “reproducibility of results”[MeSH] OR reproducib*[tiab] OR “discriminant analysis”[MeSH] OR reliab*[tiab] OR unreliab*[tiab] OR valid*[tiab] OR “coefficient of variation”[tiab] OR coefficient[tiab] OR homogeneity[tiab] OR homogeneous[tiab] OR “internal consistency”[tiab] OR (cronbach*[tiab] AND (alpha[tiab] OR alphas[tiab])) OR (item[tiab] AND (correlation*[tiab] OR selection*[tiab] OR reduction*[tiab])) OR agreement[tw] OR precision[tw] OR imprecision[tw] OR “precise values”[tw] OR test-retest[tiab] OR (test[tiab] AND retest[tiab]) OR (reliab*[tiab] AND (test[tiab] OR retest[tiab])) OR stability[tiab] OR interrater[tiab] OR inter-rater[tiab] OR intrarater[tiab] OR intra-rater[tiab] OR intertester[tiab] OR inter-tester[tiab] OR intratester[tiab] OR intra-tester[tiab] OR interobserver[tiab] OR inter-observer[tiab] OR intraobserver[tiab] OR intra-observer[tiab] OR intertechnician[tiab] OR inter-technician[tiab] OR intratechnician[tiab] OR intra-technician[tiab] OR interexaminer[tiab] OR inter-examiner[tiab] OR intraexaminer[tiab] OR intra-examiner[tiab] OR interassay[tiab] OR inter-assay[tiab] OR intraassay[tiab] OR intra-assay[tiab] OR interindividual[tiab] OR inter-individual[tiab] OR intraindividual[tiab] OR intra-individual[tiab] OR interparticipant[tiab] OR inter-participant[tiab] OR intraparticipant[tiab] OR intra-participant[tiab] OR kappa[tiab] OR kappa’s[tiab] OR kappas[tiab] OR repeatab*[tw] OR ((replicab*[tw] OR repeated[tw]) AND (measure[tw] OR measures[tw] OR findings[tw] OR result[tw] OR results[tw] OR test[tw] OR tests[tw])) OR generaliza*[tiab] OR generalisa*[tiab] OR concordance[tiab] OR (intraclass[tiab] AND correlation*[tiab]) OR discriminative[tiab] OR “known group”[tiab] OR “factor analysis”[tiab] OR “factor analyses”[tiab] OR “factor structure”[tiab] OR “factor structures”[tiab] OR dimension*[tiab] OR subscale*[tiab] OR (multitrait[tiab] AND scaling[tiab] AND (analysis[tiab] OR analyses[tiab])) OR “item discriminant”[tiab] OR “interscale correlation*”[tiab] OR error[tiab] OR errors[tiab] OR “individual variability”[tiab] OR “interval variability”[tiab] OR “rate variability”[tiab] OR (variability[tiab] AND (analysis[tiab] OR values[tiab])) OR (uncertainty[tiab] AND (measurement[tiab] OR measuring[tiab])) OR “standard error of measurement”[tiab] OR sensitiv*[tiab] OR responsive*[tiab] OR (limit[tiab] AND detection[tiab]) OR “minimal detectable concentration”[tiab] OR interpretab*[tiab] OR ((minimal[tiab] OR minimally[tiab] OR clinical[tiab] OR clinically[tiab]) AND (important[tiab] OR significant[tiab] OR detectable[tiab]) AND (change[tiab] OR difference[tiab])) OR (small*[tiab] AND (real[tiab] OR detectable[tiab]) AND (change[tiab] OR difference[tiab])) OR “meaningful change”[tiab] OR “ceiling effect”[tiab] OR “floor effect”[tiab] OR “Item response model”[tiab] OR IRT[tiab] OR Rasch[tiab] OR “Differential item functioning”[tiab] OR DIF[tiab] OR “computer adaptive testing”[tiab] OR “item bank”[tiab] OR “cross-cultural equivalence”[tiab])

**PsycInfo**

(exercise OR "physical activity" OR "physical activities" OR "sedentary behavior OR "sedentary behaviors" OR "sedentary behaviour" OR "sedentary behaviours" OR "sitting position" OR "screen time" OR sedentar* OR sleep OR "movement behavio*" OR "movement behaviou*" OR inactiv* OR bedtime OR bedtime* OR "time in bed")

AND

(surveys OR survey OR surveillance OR questionnaire OR questionnaires OR "proxy-reported" OR "proxy-report" OR "proxy-reports" OR "proxy report" OR "proxy reported" OR instrument OR instruments OR measur* OR scale OR scales OR assess* OR tool OR tools OR Interview* OR "self-report" OR "self-reported" OR "self-reports" OR "self report" OR "self reported" [Title/Abstract])

AND

(“psychometrics” OR psychometr* OR clinimetr* OR clinometr* OR “outcome assessment” OR “outcome measure*” OR “observer variation” OR “observer variation” OR “Health Status Indicators” OR “reproducibility of results” OR reproducib* OR “discriminant analysis” OR reliab*OR unreliab* OR valid* OR “coefficient of variation” OR coefficient OR homogeneity OR homogeneous OR “internal consistency”OR (cronbach* AND (alpha OR alphas)) OR (item AND (correlation* OR selection* OR reduction*)) OR agreement OR precision OR imprecision OR “precise values” OR test-retest OR (test AND retest) OR (reliab* AND (test OR retest)) OR stability OR interrater OR inter-rater OR intrarater OR intra-rater OR intertester OR inter-tester OR intratester[tiab] OR intra-tester OR interobserver OR inter-observer OR intraobserver OR intra-observer OR intertechnician OR inter-technician OR intratechnician OR intra-technician OR interexaminer OR inter-examiner OR intraexaminer OR intra-examiner OR interassay OR inter-assay OR intraassay OR intra-assay OR interindividual OR inter-individual OR intraindividual OR intra-individual OR interparticipant OR inter-participant OR intraparticipant OR intra-participant OR kappa OR kappa’s OR kappas OR repeatab* OR ((replicab* OR repeated) AND (measure OR measures OR findings OR result OR results OR test OR tests)) OR generaliza*OR generalisa* OR concordance OR (intraclass AND correlation*) OR discriminative OR “known group” OR “factor analysis” OR “factor analyses” OR “factor structure” OR “factor structures” OR dimension*OR subscale* OR (multitrait AND scaling AND (analysis OR analyses)) OR “item discriminant” OR “interscale correlation*” OR error OR errors OR “individual variability” OR “interval variability” OR “rate variability” OR (variability AND (analysis OR values)) OR (uncertainty AND (measurement OR measuring)) OR “standard error of measurement” OR sensitiv* OR responsive* OR (limit AND detection) OR “minimal detectable concentration” OR interpretab* OR ((minimal OR minimally OR clinical OR clinically) AND (important OR significant OR detectable) AND (change OR difference)) OR (small* AND (real OR detectable) AND (change OR difference)) OR “meaningful change” OR “ceiling effect” OR “floor effect” OR “Item response model” OR IRT OR Rasch OR “Differential item functioning” OR DIF OR “computer adaptive testing” OR “item bank” OR “cross-cultural equivalence”)

**CINHAL**

(exercise OR "physical activity" OR "physical activities" OR "sedentary behavior OR "sedentary behaviors" OR "sedentary behaviour" OR "sedentary behaviours" OR "sitting position" OR "screen time" OR sedentar* OR sleep OR "movement behavio*" OR "movement behaviou*" [Title/Abstract] OR inactiv* OR bedtime OR bedtime* OR "time in bed")

AND

(surveys OR survey OR surveillance OR questionnaire OR questionnaires OR "proxy-reported" OR "proxy-report" OR "proxy-reports" OR "proxy report" OR "proxy reported" OR instrument OR instruments OR measur* OR scale OR scales OR assess* OR tool OR tools OR Interview* OR "self-report" OR "self-reported" OR "self-reports" OR "self report" OR "self reported" [Title/Abstract])

AND

(“psychometrics” OR psychometr* OR clinimetr* OR clinometr* OR “outcome assessment” OR “outcome measure*” OR “observer variation” OR “observer variation” OR “Health Status Indicators” OR “reproducibility of results” OR reproducib* OR “discriminant analysis” OR reliab*OR unreliab* OR valid* OR “coefficient of variation” OR coefficient OR homogeneity OR homogeneous OR “internal consistency”OR (cronbach* AND (alpha OR alphas)) OR (item AND (correlation* OR selection* OR reduction*)) OR agreement OR precision OR imprecision OR “precise values” OR test-retest OR (test AND retest) OR (reliab* AND (test OR retest)) OR stability OR interrater OR inter-rater OR intrarater OR intra-rater OR intertester OR inter-tester OR intratester[tiab] OR intra-tester OR interobserver OR inter-observer OR intraobserver OR intra-observer OR intertechnician OR inter-technician OR intratechnician OR intra-technician OR interexaminer OR inter-examiner OR intraexaminer OR intra-examiner OR interassay OR inter-assay OR intraassay OR intra-assay OR interindividual OR inter-individual OR intraindividual OR intra-individual OR interparticipant OR inter-participant OR intraparticipant OR intra-participant OR kappa OR kappa’s OR kappas OR repeatab* OR ((replicab* OR repeated) AND (measure OR measures OR findings OR result OR results OR test OR tests)) OR generaliza*OR generalisa* OR concordance OR (intraclass AND correlation*) OR discriminative OR “known group” OR “factor analysis” OR “factor analyses” OR “factor structure” OR “factor structures” OR dimension*OR subscale* OR (multitrait AND scaling AND (analysis OR analyses)) OR “item discriminant” OR “interscale correlation*” OR error OR errors OR “individual variability” OR “interval variability” OR “rate variability” OR (variability AND (analysis OR values)) OR (uncertainty AND (measurement OR measuring)) OR “standard error of measurement” OR sensitiv* OR responsive* OR (limit AND detection) OR “minimal detectable concentration” OR interpretab* OR ((minimal OR minimally OR clinical OR clinically) AND (important OR significant OR detectable) AND (change OR difference)) OR (small* AND (real OR detectable) AND (change OR difference)) OR “meaningful change” OR “ceiling effect” OR “floor effect” OR “Item response model” OR IRT OR Rasch OR “Differential item functioning” OR DIF OR “computer adaptive testing” OR “item bank” OR “cross-cultural equivalence”)

**SportDiscus**

(exercise OR "physical activity" OR "physical activities" OR "sedentary behavior OR "sedentary behaviors" OR "sedentary behaviour" OR "sedentary behaviours" OR "sitting position" OR "screen time" OR sedentar* OR sleep OR "movement behavio*" OR "movement behaviou*" [Title/Abstract] OR inactiv* OR bedtime OR bedtime* OR "time in bed")

AND

(surveys OR survey OR surveillance OR questionnaire OR questionnaires OR "proxy-reported" OR "proxy-report" OR "proxy-reports" OR "proxy report" OR "proxy reported" OR instrument OR instruments OR measur* OR scale OR scales OR assess* OR tool OR tools OR Interview* OR "self-report" OR "self-reported" OR "self-reports" OR "self report" OR "self reported" [Title/Abstract])

AND

(“psychometrics” OR psychometr* OR clinimetr* OR clinometr* OR “outcome assessment” OR “outcome measure*” OR “observer variation” OR “observer variation” OR “Health Status Indicators” OR “reproducibility of results” OR reproducib* OR “discriminant analysis” OR reliab*OR unreliab* OR valid* OR “coefficient of variation” OR coefficient OR homogeneity OR homogeneous OR “internal consistency”OR (cronbach* AND (alpha OR alphas)) OR (item AND (correlation* OR selection* OR reduction*)) OR agreement OR precision OR imprecision OR “precise values” OR test-retest OR (test AND retest) OR (reliab* AND (test OR retest)) OR stability OR interrater OR inter-rater OR intrarater OR intra-rater OR intertester OR inter-tester OR intratester[tiab] OR intra-tester OR interobserver OR inter-observer OR intraobserver OR intra-observer OR intertechnician OR inter-technician OR intratechnician OR intra-technician OR interexaminer OR inter-examiner OR intraexaminer OR intra-examiner OR interassay OR inter-assay OR intraassay OR intra-assay OR interindividual OR inter-individual OR intraindividual OR intra-individual OR interparticipant OR inter-participant OR intraparticipant OR intra-participant OR kappa OR kappa’s OR kappas OR repeatab* OR ((replicab* OR repeated) AND (measure OR measures OR findings OR result OR results OR test OR tests)) OR generaliza*OR generalisa* OR concordance OR (intraclass AND correlation*) OR discriminative OR “known group” OR “factor analysis” OR “factor analyses” OR “factor structure” OR “factor structures” OR dimension*OR subscale* OR (multitrait AND scaling AND (analysis OR analyses)) OR “item discriminant” OR “interscale correlation*” OR error OR errors OR “individual variability” OR “interval variability” OR “rate variability” OR (variability AND (analysis OR values)) OR (uncertainty AND (measurement OR measuring)) OR “standard error of measurement” OR sensitiv* OR responsive* OR (limit AND detection) OR “minimal detectable concentration” OR interpretab* OR ((minimal OR minimally OR clinical OR clinically) AND (important OR significant OR detectable) AND (change OR difference)) OR (small* AND (real OR detectable) AND (change OR difference)) OR “meaningful change” OR “ceiling effect” OR “floor effect” OR “Item response model” OR IRT OR Rasch OR “Differential item functioning” OR DIF OR “computer adaptive testing” OR “item bank” OR “cross-cultural equivalence”)
