# Supplementary material for: Questionnaires measuring movement behaviours in adults and older adults: Content description and measurement properties. A systematic review
Source: PLoS One. 2022 Mar 11;17(3):e0265100. doi: 10.1371/journal.pone.0265100 (PMC8916622; doi:10.1371/journal.pone.0265100)
Supplement: S2 File — (DOCX) [file pone.0265100.s003.docx]

**Supporting file 2 – Extracted information**

For the content description, we extracted information on the name of the questionnaire:

1 - Age group and country: If the questionnaire was validated in adults, older adults, or both.

2 - Domains: Which domains the questionnaire refers to (e.g., leisure, occupational, transportation, etc.). When there was no distinction between domains, we reported total sleep/ sedentary behaviour/physical activity.

1. - Response method: What kind of response the questionnaire allows for (e.g., rating scale or continuous response).
2. - Measurement units: Which unit the researchers must use to evaluate the movement behaviours (e.g., METs, minutes/day; hours/week, etc.).
3. - Scores: What are the final outputs from the questionnaire (e.g., total sleep/ sedentary behaviour/physical activity; sleep duration; occupational physical activity/ sedentary behaviour, activity level index, etc.).
4. - Recall period/Assessment period: To which period the questionnaire refers to (e.g., typical/last day/week, on week and weekend or whole day/week without distinction for the types of days, etc.);
5. - Number of items: How many items compose the questionnaire.
6. - Parameters: Frequency, duration, intensity and mode.

For the measurement properties, we extracted information about:

1. Sample size and demographic characteristics (e.g., gender and age).
2. Validity: The degree to which an instrument truly measures the construct(s) that wants to measure, free from all possible sources of error or bias.
   1. Type of validity: Criterion (e.g., the extent of the correlation between a measure and another already considered as being a criterion or gold standard) or convergent (e.g., the extent of the agreement with another (non-criterion) measure that should assess the same behaviour parameter based on face and content validity).
   2. Comparison measure: Which instrument was used to validation (e.g., accelerometer, another questionnaire, etc.).
   3. Results: Validity results per variable (e.g., pearson/spearman correlations, Bland Altman, etc.).
   4. – Quality of study: Explained below.
3. Reliability:
   1. Time between test and retest.
   2. Results: Pearson and Spearman correlations, intraclass correlations, kappas, measurement error (e.g., limits of agreement, etc.).
4. Responsiveness:
   1. Type of responsiveness.
   2. Results: Effect tests, etc.
