# Supplementary material for: Questionnaires measuring movement behaviours in adults and older adults: Content description and measurement properties. A systematic review
Source: PLoS One. 2022 Mar 11;17(3):e0265100. doi: 10.1371/journal.pone.0265100 (PMC8916622; doi:10.1371/journal.pone.0265100)
Supplement: S2 Table — (DOCX) [file pone.0265100.s005.docx]

**Supporting table 2 – Validity results**

| **Questionnaire** | Sample | Validity | | | Quality of validity results | Overall Quality |
| --- | --- | --- | --- | --- | --- | --- |
|  | n; % Women; Age mean ± SD or Age range (years) | Type of Validity | Comparison Measure | Results |  |  |
| **Physical Activity** | | | | | | |
| Nord-Trøndelag Health Study PA Questionnaire (HUNT 1)^30^ | 108 men; 20 - 39 yrs | Criterion | Accelerometer (ActiReg), 7 days on hip | Frequency *rho* = 0.03 | - | - |
|  |  |  |  | Intensity *rho* = 0.06 | - |  |
|  |  |  |  | Duration *rho* = 0.12 | - |  |
|  |  |  |  | Index *rho* = 0.07 | - |  |
|  |  |  |  | 1-3 METs *rho* = -0.06 | - |  |
|  |  |  |  | 3-6 METs 6+ *rho* = 0.32* | - |  |
|  |  | Convergent | IPAQ | Frequency: |  | - |
|  |  |  |  | IPAQ Total VPA *rho* = 0.44** | - |  |
|  |  |  |  | IPAQ Total MPA *rho* = 0.31** | - |  |
|  |  |  |  | IPAQ Walking *rho* = 0.26** | - |  |
|  |  |  |  | IPAQ METs *rho* = 0.28* | - |  |
|  |  |  |  | Intensity: |  |  |
|  |  |  |  | IPAQ Total VPA *rho* = 0.49** | - |  |
|  |  |  |  | IPAQ Total MPA *rho* = 0.18 | - |  |
|  |  |  |  | IPAQ Walking *rho* = 0.18 | - |  |
|  |  |  |  | IPAQ METs *rho* = 0.32** | - |  |
|  |  |  |  | Duration: |  |  |
|  |  |  |  | IPAQ Total VPA *rho* = 0.41** | - |  |
|  |  |  |  | IPAQ Total MPA *rho* = 0.21* | - |  |
|  |  |  |  | IPAQ Walking *rho* = 0.06 | - |  |
|  |  |  |  | IPAQ METs *rho* = 0.20 | - |  |
|  |  |  |  | Index: |  |  |
|  |  |  |  | IPAQ Total VPA *rho* = 0.55** | - |  |
|  |  |  |  | IPAQ Total MPA *rho* = 0.30* | - |  |
|  |  |  |  | IPAQ Walking *rho* = 0.18 | - |  |
|  |  |  |  | IPAQ METs *rho* = 0.32** | - |  |
|  |  |  | Vo2max | Frequency *rho* = 0.43* | - | - |
|  |  |  |  | Intensity *rho* = 0.40* | - |  |
|  |  |  |  | Duration *rho* = 0.31* | - |  |
|  |  |  |  | Index *rho* = 0.48* | - |  |
| Past Year Total Physical Activity Questionnaire (PYTPAQ)^31^ | 154; 51% women, 48.7 ± 7.7 yrs | Criterion | Accelerometer (Actigraph), 7 days, minute-by-minute observations of whole-body motion, on waist. | Total population *rho* = 0.26*; | - | - |
|  |  |  |  | <50 yr *rho* = 0.43**; >50 yr = 0.05 | - |  |
|  |  | Convergent | 7-day physical activity log | Total population *rho* = 0.41***; | - | - |
|  |  |  |  | <50 yr *rho* = 0.53**; >50 yr = 0.26* | - |  |
| Physical Activity Assessment Tool (PAAT)^32^ | 63; N.R.; N.R. | Criterion | Accelerometer (Actigraph, MTI) on waist | MVPA *rho* = 0.39** | - | - |
|  |  |  |  | MPA *rho* = 0.39** | - |  |
|  |  |  |  | VPA *rho* = 0.38** | - |  |
|  |  |  |  | Activity level agreement = 69.8%; *kappa* = 0.34 | - |  |
|  |  |  |  | Sensitivity and Specificity = 71.1% and 66.7% |  |  |
|  |  |  |  | Positive Predictive Value = 84.2% |  |  |
|  |  |  |  | Negative Predictive Value = 48.0% |  |  |
|  |  | Convergent | IPAQ | MVPA *r* = 0.59*** | - | - |
|  |  |  |  | Activity level agreement = 66.7%; *kappa*= 0.21 | - |  |
| Minnesota Leisure Time Physical Activity Questionnaire (Minnesota LTPA Q)^33^ | 73; N.R.; N.R. | Criterion | Accelerometer (Caltrac), 2 days on hip | Total Leisure time physical activity *rho* = 0.18 | - | - |
|  |  |  |  | LPA *rho* = 0.07 | - |  |
|  |  |  |  | MPA *rho* = 0.22 | - |  |
|  |  |  |  | VPA *rho* = 0.16 | - |  |
|  |  |  |  | Household chores *rho* = 0.03 | - |  |
|  |  | Convergent | 4-week history | Total Leisure time physical activity *rho* = 0.74 | - | - |
|  |  |  |  | LPA *rho* = 0.13 | - |  |
|  |  |  |  | MPA *rho* = 0.25 | - |  |
|  |  |  |  | VPA *rho* = 0.52 | - |  |
|  |  |  |  | Household chores *rho* = 0.55 | - |  |
|  |  |  | Vo2max | Total Leisure time physical activity *rho* = 0.43 | - | - |
|  |  |  |  | LPA *rho* = 0.11 | - |  |
|  |  |  |  | MPA *rho* = 0.15 | - |  |
|  |  |  |  | VPA *rho* = 0.39 | - |  |
|  |  |  |  | Household chores *rho* = 0.18 | - |  |
| Single Item Physical Activity Measure (SI PA M)^34^ | 522; N.R.; N.R. | Convergent | Global Physical Activity Questionnaire | Physical activity week (past week question) *rho* = 0.53 | - | - |
|  |  |  | Active People Survey | Physical activity month (past month question) *rho* = 0.48 | - | - |
| Godin Questionnaire (Godin Q)^33^ | 73; N.R.; N.R. | Criterion | Accelerometer (Caltrac), 2 days on hip | Leisure score *rho* = 0.32 | - | - |
|  |  | Convergent | 4-week history | Leisure score *rho* = 0.36 | - | - |
|  | 64; N.R.; N.R. |  | Vo2max | Leisure score *rho* = 0.56 | - | - |
| CARDIA Physical Activity History (CARDIA)^4^ | 73; N.R.; N.R. | Criterion | Accelerometer (Caltrac), 2 days on hip | MPA *rho* = 0.11 | - | - |
|  |  |  |  | VPA *rho* = 0.31 | - |  |
|  |  | Convergent | 4-week history | MPA *rho* = 0.08 | - | - |
|  |  |  |  | VPA *rho* = 0.54 | - |  |
|  | 64; N.R.; N.R. |  | Vo2max | MPA *rho* = 0.08 | - | - |
|  |  |  |  | VPA *rho* = 0.63 | + |  |
| College Alumnus Questionnaire (College Alumnus Q)^33^ | 73; N.R.; N.R. | Criterion | Accelerometer (Caltrac), 2 days on hip | Total index *rho* = 0.30 | - | - |
|  |  |  |  | Stairs *rho* = -0.01 | - |  |
|  |  |  |  | Blocks *rho* = 0.16 | - |  |
|  |  | Convergent | 4-week history | Total index *rho* = 0.31 | - | - |
|  |  |  |  | Stairs *rho* = 0.25 | - |  |
|  |  |  |  | Blocks *rho* = 0.06 | - |  |
|  | 64; N.R.; N.R. |  | Vo2máx | Total index *rho* = 0.52 | - | - |
|  |  |  |  | Stairs *rho* = 0.02 | - |  |
|  |  |  |  | Blocks *rho* = -0.01 | - |  |
| Minnesota Heart Health Program Questionnaire (MHHP Q)^33^ | 73; N.R.; N.R. | Criterion | Accelerometer (Caltrac), 2 days on hip | Work index *rho* = 0.04 | - | - |
|  |  |  |  | Leisure index *rho* = 0.28 | - |  |
|  |  | Convergent | 4-week history | Work index *rho* = -0.09 | - | - |
|  |  |  |  | Leisure index *rho* = 0.39 | - |  |
|  |  |  | Vo2max | Work index *rho* = 0.00 | - | - |
|  |  |  |  | Leisure index *rho* = 0.56 | + |  |
| Modified Historical Leisure Activity Questionnaire (MHLAQ)^35^ | 131; 100 % women; 50.4 ± 7.6 yrs | Convergent | Physical activity log | <50 yr: |  | - |
|  |  |  |  | Total physical activity *r* = 0.31* | - |  |
|  |  |  |  | MPA *r* = 0.16 | - |  |
|  |  |  |  | VPA *r* = 0.58** | - |  |
|  |  |  |  | >50 yr: |  |  |
|  |  |  |  | Total physical activity *r* = 0.19 | - |  |
|  |  |  |  | MPA *r* = 0.12 | - |  |
|  |  |  |  | VPA *r* = 0.47** | - |  |
| Modified version Active Australia Survey 1(MV – AAS1)^36^ | 159; 100% women; 54-59yrs | Criterion | Pedometer | walking *rho*=0.29*** | - | - |
|  |  |  |  | Total physical activity *rho*=0.43*** | - |  |
| Modified version Active Australia Survey (MV – AAS2)^37^ | 63; 63% women; 49.5 ± 12.5 yrs | Criterion | Accelerometer (MTI Model 7164), on hip | Mins/week MPA *rho*= 0.50 (95% CI: 0.28 to 0.66) | - | - |
|  |  |  |  | Mins/week VPA *rho*= 0.60 (95% CI: 0.41 to 0.74) | - |  |
|  |  |  |  | Mins/week MVPA *rho*= 0.61 (95% CI: 0.43 to 0.75) | - |  |
|  |  |  |  | Days/week MPA *kappa* = 0.35 (95% CI: 0.10 to 0.50) | - |  |
|  |  |  |  | Days/week VPA *kappa*= 0.61 (95% CI: 0.29 to 0.87) | - |  |
|  |  |  |  | Days/week MVPA *kappa*= 0.45 (95% CI: 0.34 to 0.66) | - |  |
|  |  |  |  | Bland-Altman: *LoA*= 22.60 ± 69.94 + 1.01 x average Australian Active Survey & accelerometer). |  | |
| Adapted from Active Australia Survey (Adapt AAS)^38^ | 122; 53% women; 38 ± 15 yrs | Criterion | Accelerometer (MTI Model 7164), on hip | Mins/day men MPA *rho*=0.40** | - | - |
|  |  |  |  | Mins/day men VPA *rho*= 0.19 | - |  |
|  |  |  |  | Mins/day men MVPA *rho*= 0.29* | - |  |
|  |  |  |  | Mins/day women MPA *rho*= 0.19 | - |  |
|  |  |  |  | Mins/day women VPA *rho*= 0.10 | - |  |
|  |  |  |  | Mins/day women MVPA *rho*= 0.25* | - |  |
| International Physical Activity Questionnaire – Walking Section (IPAQ-WS)^39^ | 247; 55% women; 23-57 yrs | Criterion | Accelerometer (Actigraph), 5 days | Past week walking *rho* = 0.39 | - | - |
|  |  |  |  | Past week walk+MPA *rho*= 0.24 | - |  |
|  |  |  |  | Usual week walk+MPA *rho*= 0.04 | - |  |
|  |  |  |  | Usual week walking *rho* = 0.26 | - |  |
| Short Questionnaire  to Assess Health-enhancing physical activity (SQUASH)^40^ | 50; 28% women; 44 ± 6 yrs | Criterion | Accelerometer, (CSA Inc. model AM7164-2.2), 1 min epoch, 2 weeks, on waist | Activity levels *rho* = 0.45* (95% CI: 0.17 to 0.66) | - | - |
|  |  |  |  | Tertiles of the activity score *rho* = 46%; weighed *kappa* was 0.30 | - |  |
| European Prospective Investigation into Cancer and Nutrition Physical Activity Questionnaire (EPIC PAQ)^41^ | 182; 45% women; 50-65 yrs | Criterion | Accelerometer (Actigraph), 7 days | Total non-occup physical activity *rho* = 0.21** (95% CI: 0.07 to 0.35) | - | - |
|  |  |  |  | VPA (self-rated) *rho* = 0.18* (95% CI: 0.04 to 0.32 | - |  |
|  |  |  |  | VPA (MET-assigned) *rho* = 0.23** (95% CI: 0.09 to 0.37) | - |  |
|  |  |  |  | LPA to MPA *rho* = 0.19** (95% CI: 0.05 to 0.33) | - |  |
|  |  |  |  | Bland Altman: Mean difference: 66.4 ± 67.9; 95% *LoA*: -66.7 to 199.6 |  |  |
|  |  | Convergent | Friedenreich LTPAQ | Total non-occup physical activity *rho* = 0.26*** (0.11 to 0.39) | - | - |
|  |  |  |  | VPA *rho* = 0.40*** (95% CI: 0.27 to 0.52) | - |  |
|  |  |  |  | LPA to MPA *rho* = 0.26*** (95% CI: 0.12 to 0.39) | - |  |
|  |  |  |  | Household physical activity *rho* = 0.46*** (95% CI: 0.34 to 0.57) | - |  |
|  |  |  |  | Recreational physical activity *rho* = 0.21* (95% CI: 0.07 to 0.34) | - |  |
| 13-Item Physical Activity Questionnaire (13I-PAQ)^42^ | 54, N.R. | Convergent |  | Women |  | - |
|  |  |  | Body Fat | Total physical activity *r* = - 0.73* | + |  |
|  |  |  |  | Sport *r* = - 0.55* | + |  |
|  |  |  |  | LTPA *r* = 0.06 | - |  |
|  |  |  |  | Occupational and household physical activity *r*= - 0.53* | + |  |
|  |  |  | VO2Max | Total physical activity *r* = 0.63* | + |  |
|  |  |  |  | Sport *r* = 0.67* | + |  |
|  |  |  |  | LTPA *r* = - 0.14 | - |  |
|  |  |  |  | Occupational and household physical activity *r*= 0.27 | - |  |
|  |  |  | Flexibility | Total physical activity *r* = 0.28 | - |  |
|  |  |  |  | Sport *r* = 0.18* | - |  |
|  |  |  |  | LTPA *r* = 0.25 | - |  |
|  |  |  |  | Occupational and household physical activity = 0.02 | - |  |
|  |  |  |  | Men |  |  |
|  |  |  | Body Fat | Total physical activity *r* = - 0.46* | - |  |
|  |  |  |  | Sport *r* = -0.43* | - |  |
|  |  |  |  | LTPA *r* = 0.10 | - |  |
|  |  |  |  | Occupational and household physical activity = -0.16 | - |  |
|  |  |  | VO2Max | Total physical activity *r* = 0.57* | + |  |
|  |  |  |  | Sport *r* = 0.67* | - |  |
|  |  |  |  | LTPA *r* = 0.09 | - |  |
|  |  |  |  | Occupational and household physical activity *r* = 0.04 | - |  |
|  |  |  | Flexibility |  |  |  |
|  |  |  |  | Total physical activity *r* = 0.39* | - |  |
|  |  |  |  | Sport *r* = 0.39* | - |  |
|  |  |  |  | LTPA *r* = 0.19 | - |  |
|  |  |  |  | Occupational and household physical activity = 0.06 | - |  |
|  |  |  |  |  |  |  |
| Questionnaire d’Activité Physique pour les Personnes Âgées (QAPPA)^43^ | 393; 64.1% women; 70.1 ± 8 yrs | Convergent |  | Moderate (METM): |  | - |
|  |  |  | Questionnaire (exercise behaviour [yes/no] in the last 2 months) | Significant difference between exercisers and non-exercisers (Wilcoxon rank sum test) |  |  |
|  |  |  | Satisfaction with body functioning | *rho* = 0.16** | - |  |
|  |  |  | Decline in physical function | *rho* = -0.21** | - |  |
|  |  |  | Age | *rho* = -0.13**. | - |  |
|  |  |  |  | Vigorous (METV): |  |  |
|  |  |  | Questionnaire (exercise behaviour [yes/no] in the last 2 months) | Significant difference between exercisers and non-exercisers (Wilcoxon rank sum test) |  |  |
|  |  |  | Satisfaction with body functioning | *rho* = 0.39** | - |  |
|  |  |  | Decline in physical function | *rho* = -0.32** | - |  |
|  |  |  | Age | *rho* = -0.30** | - |  |
|  |  |  |  | Moderate-to-vigorous (METT): |  |  |
|  |  |  | Questionnaire (exercise behaviour [yes/no] in the last 2 months) | Significant difference between exercisers and non-exercisers (Wilcoxon rank sum test) |  |  |
|  |  |  | Satisfaction with body functioning | *rho* = 0.45** | - |  |
|  |  |  | Decline in physical function | *rho* = -0.40** | - |  |
|  |  |  | Age | *rho* = -0.29**. | - |  |
|  |  |  | Classification (active/inactive): | Significant difference between exercisers and non-exercisers (Chi-squared test) |  |  |
| Incidental and Planned Exercise Questionnaire (IPEQ)^44^ | IPEQ- 3 months n = 230; 54% women; N.R. | Convergent | IPEQ-3 months vs IPEQ-Week (n = 50) | Total (hours per week) *rho* = 0.67; | - | - |
|  | IPEQ-Week n = 270; 57% women; N.R. | Convergent |  | Total (hours of planned and incidental physical activity per week) *rho* = 0.73 | + |  |
|  |  |  | WHODAS | Planned physical activity (both versions); Incidental physical activity (IPEQ-Week): Significant difference between score >20 |  |  |
|  |  |  | Time up-and-go | Planned physical activity (both versions): Significant difference between time>10 seconds |  |  |
|  |  |  | Age | Planned physical activity (both versions): Significant difference between age >75 |  |  |
| Physical Activity Questionnaire for Elderly Japanese (PAQ-EJ)^45^ | 147; 58.5% women; 65-85 yrs | Criterion | Accelerometer, on waist, 1 month, 4 seconds | Total PAQ-EJ rank-ordered *r* (month averaged daily step count) = 0.41 | - | - |
|  |  |  |  | Subtotal of lower intensity activity categories (objective month-averaged record for the daily duration of physical activity <3 METs) *r* = 0.28 | - |  |
|  |  |  |  | Subtotal of higher intensity activity categories (objective measurements for the duration of activity ≥3 METs) *r* = 0.53 | - |  |
| The Longitudinal Ageing Study Amsterdam Physical Activity Questionnaire (LAPAQ)^46^ | 88; 52% women; 65–88 yrs | Criterion | Accelerometer (Sensewear® Pro Armband Accelerometer BodyMedia, Inc), on arm | Total physical activity *r* = 0.25 (95% CI: 0.07 to 0.44) | - | - |
|  |  |  |  | >= 6 METs *r* = 0.01 (95% CI: -0.07 to -0.25) | - |  |
|  |  |  |  | 3-5.99 METs *r* = 0.27 (95% CI: 0.07 to 0.25) | - |  |
|  |  |  |  | 2-2.99 METs *r* = 0.05 (95% CI: -0.16 to -0.24) | - |  |
|  |  |  |  | The Bland–Altman plot shows much spread around the regression line and the range between the limits of agreement is wide, indicating measurement error in the LAPAQ |  |  |
|  |  |  |  | ROC curve: AUC = 0.73 (95% CI 0.59 to 0.86) | + |  |
| Nordic Physical Activity Questionnaire (NPAQ-short)^47^ | 92; N.R.; N.R. | Criterion | Accelerometer (Actiheart), 60 seconds epoch, on chest | MVPA open questions weighted *kappa* = 0.17 (95% CI: 0.07 to 0.30) | - | - |
|  |  |  |  | MVPA close questions weighted *kappa* = 0.20 (95% CI: 0.07 to 0.36) | - |  |
|  |  |  |  | VPA open questions weighted *kappa* = 0.21 (95% CI: 0.08 to 0.37) | - |  |
|  |  |  |  | VPA close questions weighted *kappa* = 0.20 (95% CI: 0.06 to 0.34) | - |  |
|  |  |  |  | Compliance with WHO guidelines *Kappa* (open questions) = 0.42 (95% CI: 0.19 to 0.65); Specificity = 50%; Sensitivity = 90% | - |  |
|  |  |  |  | Compliance with WHO guidelines *Kappa* (close questions) = 0.34 (95% CI: 0.12 to 0.56); Specificity = 55%; Sensitivity = 81% | - |  |
|  |  |  |  | Compliance with WHO guidelines *Kappa* (both questions’ type) = 0.37 (95% CI: 0.14 to 0.61); Specificity = 40%; Sensitivity = 98% | - |  |
|  |  |  |  | Bland-Altman: MVPA mean difference: − 111 min (*LoA*: − 680 to 457) |  |  |
|  |  |  |  | Bland-Altman: VPA min difference: 26 min (*LoA*: -173 to -224) |  |  |
| Self-report physical activity questionnaire (SPAQ)^48^ | 150; 73.3% women; 60- over 80 yrs | Criterion | Accelerometer (Actigraph), on hip | Activity counts/week: |  | - |
|  |  |  |  | Total physical activity *r* = 0.31** | - |  |
|  |  |  |  | LPA *r* = 0.24** | - |  |
|  |  |  |  | MPA *r* = 0.31** | - |  |
|  |  |  |  | Household physical activity *r* = 0.19* | - |  |
|  |  |  |  | Occupational physical activity *r* = 0.19* | - |  |
|  |  |  |  | Leisure time recreation *r* = 0.18** | - |  |
|  |  |  |  | Leisure time exercise *r* = 0.24** | - |  |
|  |  |  |  | Transportation physical activity *r* = 0.20* | - |  |
|  |  |  |  | Total MET hour/week: |  |  |
|  |  |  |  | LPA *r* = 0.90** | + |  |
|  |  |  |  | MPA *r* = 0.87** | + |  |
|  |  |  |  | Household physical activity *r* = 0.83** | + |  |
|  |  |  |  | Occupational physical activity *r* = 0.31** | - |  |
|  |  |  |  | Leisure time recreation *r* = 0.52** | - |  |
|  |  |  |  | Leisure time exercise *r* = 0.69** | - |  |
|  |  |  |  | Transportation physical activity *r* = 0.56** | - |  |
| Transport and Physical Activity Questionnaire (TPAQ)^49^ | 54; 64.8% female, <30 - ≥65 yrs | Criterion | Accelerometer (Actigraph GT3X), 10 seconds epoch, 7 days on hip | MPA *rho* = 0.24 | - | - |
|  |  |  |  | VPA *rho* = 0.72*** | + |  |
|  |  |  |  | MVPA *rho* = 0.27* | - |  |
|  |  |  |  | MPA including cycling *rho* = 0.10 | - |  |
|  |  |  |  | MVPA including cycling *rho* = 0.09 | - |  |
|  |  |  |  | Bland-Altman: A mean overestimation of self-reported MVPA of 87.6 min/week (p =0.02) was observed and the 95% limits of agreement were wide (2447.1 to +622.3 min/week) |  |  |
| General Practice Physical Activity Questionnaire (GPPAQ)^50^ | 298; 54% women; 60-74 yrs | Criterion | Accelerometer (Actigraph GT3X+), 5 seconds epochs | Agreement of achieving guidelines = 24% |  | |
|  |  |  |  | Categorical Achievement: |  |  |
|  |  |  |  | GPPAQ Inactive = 53% |  |  |
|  |  |  |  | GPPAQ Moderately inactive = 12 % |  |  |
|  |  |  |  | GPPAQ Moderately active = 19% |  |  |
|  |  |  |  | GPPAQ Active = 16% |  |  |
|  |  |  |  | Sensitivity of GPPAQ to identify “Active” individuals = 19% |  |  |
|  |  |  |  | Specificity of GPPAQ to identify “Not active” individuals = 85% |  |  |
|  |  |  |  | For GPPAQ-WALK: |  |  |
|  |  |  |  | Categorical Achievement: |  |  |
|  |  |  |  | GPPAQ Inactive = 43% |  |  |
|  |  |  |  | GPPAQ Moderately inactive = 10 % |  |  |
|  |  |  |  | GPPAQ Moderately active = 15% |  |  |
|  |  |  |  | GPPAQ Active = 32% |  |  |
|  |  |  |  | Sensitivity of GPPAQ to identify “Active” individuals = 39% |  |  |
|  |  |  |  | Specificity of GPPAQ to identify “Not active” individuals = 70% |  |  |
| **Sedentary Behaviour** | | | | | | |
| International Physical Activity Questionnaire - Sedentary Behavior (IPAQ-SB)^51^ | 199; 57% women; N.R. | Criterion | Accelerometers (CSA model 7164) at least 5 days on of which 1 weekend day, 1-minute intervals | Short Form *rho* = 0.34 | - | - |
|  | 200; 57% women; N.R. |  |  | Long Form *rho* = 0.33 | - | - |
|  |  |  |  | Agreement between tertiles of sitting and physical inactivity categories *kappa* = 0.014 | - | - |
| Australian Longitudinal Study on Women’s Health – Sedentary Behavior Questions (ALSWH - SB Q)^52^ | 157; 100% Female; N.R. |  | 7-d behaviour log | Weekday: |  | - |
|  |  |  |  | Transport *ICC* = 0.38 (95% CI: 0.24 to 0.51) | - |  |
|  |  |  |  | Occup *ICC* = 0.69 (95% CI: 0.59 to 0.76) | - |  |
|  |  |  |  | TV *ICC* = 0.60 (95% CI: 0.49 to 0.69) | - |  |
|  |  |  |  | Computer *r* = 0.74 | + |  |
|  |  |  |  | Other leisure *ICC* = 0.27 (95% CI: 0.12 to 0.41) | - |  |
|  |  |  |  | Weekend day: |  |  |
|  |  |  |  | Transport *ICC* = 0.13 (95% CI: -0.03 to 0.28) | - |  |
|  |  |  |  | Occup *ICC* = 0.21 (95% CI: 0.05 to 0.35) | - |  |
|  |  |  |  | TV *r* = 0.53 | - |  |
|  |  |  |  | Computer *r* = 0.64 | - |  |
|  |  |  |  | Other leisure *ICC* = 0.37 (95% CI: 0.22 to 0.50) | - |  |
|  | 96; 100% men; N.R. |  | 7-d behaviour log | Weekend day: |  |  |
|  |  |  |  | Transport *ICC* = 0.63 (95% CI: 0.49 to 0.73) | - |  |
|  |  |  |  | Occup *ICC* = 0.77 (95% CI: 0.68 to 0.84) | + |  |
|  |  |  |  | TV *ICC* = 0.53 (95% CI: 0.37 to 0.66) | - |  |
|  |  |  |  | Computer *ICC* = 0.55 (95% CI: 0.40 to 0.68) | - |  |
|  |  |  |  | Other leisure *r* = 0.21 | - |  |
|  |  |  |  | Weekend day: |  |  |
|  |  |  |  | Transport *ICC* = 0.12 (95% CI: 0.08 to 0.31) | - |  |
|  |  |  |  | Occup *ICC* = 0.37 (95% CI: 0.18 to 0.53) | - |  |
|  |  |  |  | TV *ICC* = 0.32 (95% CI: 0.13 to 0.49) | - |  |
|  |  |  |  | Computer *r* = 0.61 | - |  |
|  |  |  |  | Other leisure *r* = 0.19 | - |  |
| Self-reported sitting and breaks from sitting in the workplace (SBSW)^53^ | 59; 54% female; 32.1 ± 9.9 yrs | Criterion | Accelerometer (Actigraph GT3X), on hip | Sitting *rho* = 0.39** (95% CI: 0.15 to 0.68) | - | - |
|  |  |  |  | breaks from sitting *rho* = 0.30* (95% CI: 0.15 to 0.69) | - |  |
|  |  |  | Inclinometer (activPAL3) on thigh | Sitting *rho* = 0.24 (95% CI: -1.0 to 0.47) | - |  |
|  |  |  |  | breaks from sitting *rho* = 0.39** (95% CI: 0.25 to 0.74) | - |  |
| Workplace Sitting Breaks Questionnaire (SITBRQ)^54^ | 143; 62.6 %women; ~18<60 yrs | Criterion | Accelerometer (Actigraph GT1M), 7 days on hip | Frequency of breaks *rho* = 0.24 (95% CI: 0.07 to 0.40) | - | - |
|  |  |  |  | Total duration of breaks = 0.05 (95% CI: −0.12 to 0.22) | - |  |
| Sedentary Behavior Questionnaire (SBQ)^55^ | 654; 45.9% Women; N.R. | Criterion | Accelerometer (Actigraph, Model WAM 7164); on hip, <100 counts per min | Female: Accelerometer mins with counts < 100 |  | - |
|  |  | Convergent | IPAQ | TV *r* = 0.12* | - |  |
|  |  |  |  | Computer games *r* = 0.04 | - |  |
|  |  |  |  | Sit listen to music *r* = 0.01 | - |  |
|  |  |  |  | Sit talk on telephone *r* = 0.04 | - |  |
|  |  |  |  | Office/paperwork *r* = 0.17** | - |  |
|  |  |  |  | Reading *r* = 0.01 | - |  |
|  |  |  |  | Playing musical instrument *r* = 0.26*** | - |  |
|  |  |  |  | Arts and crafts *r* = 0.06 | - |  |
|  |  |  |  | Sitting driving in car *r* = -0.04 | - |  |
|  |  |  |  | Weekday (hours/day) *r* = 0.06 | - |  |
|  |  |  |  | Weekend (hours/day) *r* = 0.18** | - |  |
|  |  |  |  | Total hours/week *r* = 0.10* | - |  |
|  |  | Criterion | Accelerometer (Actigraph, Model WAM 7164); on hip, <100 counts per min | Female: Accelerometer total activity mins/day |  | - |
|  |  |  |  | TV *r* = –0.08 | - |  |
|  |  |  |  | Computer games *r* = –0.05 | - |  |
|  |  |  |  | Sit listen to music *r* = –0.10 | - |  |
|  |  |  |  | Sit talk on telephone *r* = –0.02 | - |  |
|  |  |  |  | Office/paperwork *r* = –0.04 | - |  |
|  |  |  |  | Reading *r* = 0.01 | - |  |
|  |  |  |  | Playing musical instrument *r* = –0.05 | - |  |
|  |  |  |  | Arts and crafts *r* = –.08 | - |  |
|  |  |  |  | Sitting driving in car *r* = 0.09 | - |  |
|  |  |  |  | Weekday (hours/day) *r* = –0.07 | - |  |
|  |  |  |  | Weekend (hours/day) *r* = –0.08 | - |  |
|  |  |  |  | Total hours/week *r* = –0.08 | - |  |
|  |  | Convergent | IPAQ | Female: IPAQ ST hours/day |  | - |
|  |  |  |  | TV *r* = 0.26*** | - |  |
|  |  |  |  | Computer games *r* = 0.05 | - |  |
|  |  |  |  | Sit listen to music *r* = 0.02 | - |  |
|  |  |  |  | Sit talk on telephone *r* = 0.12* | - |  |
|  |  |  |  | Office/paperwork *r* = 0.33*** | - |  |
|  |  |  |  | Reading *r* = 0.11* | - |  |
|  |  |  |  | Playing musical instrument *r* = –0.11 | - |  |
|  |  |  |  | Arts and crafts *r* = 0.07 | - |  |
|  |  |  |  | Sitting driving in car *r* = 0.07 | - |  |
|  |  |  |  | Weekday (hours/day) *r* = .21*** | - |  |
|  |  |  |  | Weekend (hours/day) *r* = 0.36*** | - |  |
|  |  |  |  | Total hours/week *r* = 0.28*** | - |  |
|  |  | Criterion | Accelerometer (Actigraph, Model WAM 7164); on hip, <100 counts per min | Male: Accelerometer mins with counts < 100 |  | - |
|  |  |  |  | TV *r* = –0.001 | - |  |
|  |  |  |  | Computer games *r* = 0.01 | - |  |
|  |  |  |  | Sit listen to music *r* = 0.01 | - |  |
|  |  |  |  | Sit talk on telephone *r* = –0.08 | - |  |
|  |  |  |  | Office/paperwork *r* = 0.003 | - |  |
|  |  |  |  | Reading *r* = 0.01 | - |  |
|  |  |  |  | Playing musical instrument *r* = 0.04 | - |  |
|  |  |  |  | Arts and crafts *r* = –0.04 | - |  |
|  |  |  |  | Sitting driving in car *r* = 0.03 | - |  |
|  |  |  |  | Weekday (hours/day) *r* = –0.02 | - |  |
|  |  |  |  | Weekend (hours/day) *r* = –0.005 | - |  |
|  |  |  |  | Weekend (hours/day) *r* = –0.005 | - |  |
|  |  | Criterion | Accelerometer (Actigraph, Model WAM 7164); on hip, <100 counts per min | Male: Accelerometer total activity mins/day |  | - |
|  |  |  |  | TV *r* = 0.02 | - |  |
|  |  |  |  | Computer games *r* = 0.003 | - |  |
|  |  |  |  | Sit listen to music *r* = –0.02 | - |  |
|  |  |  |  | Sit talk on telephone *r* = –0.01 | - |  |
|  |  |  |  | Office/paperwork *r* = –0.004 | - |  |
|  |  |  |  | Reading *r* = –0.06 | - |  |
|  |  |  |  | Playing musical instrument *r* = 0.02 | - |  |
|  |  |  |  | Arts and crafts *r* = –0.003 | - |  |
|  |  |  |  | Sitting driving in car *r* = –0.04 | - |  |
|  |  |  |  | Weekday (hours/day) *r* =–0.03 | - |  |
|  |  |  |  | Weekend (hours/day) *r* = –0.005 | - |  |
|  |  |  |  | Total hours/week *r* = –0.03 | - |  |
|  |  | Convergent | IPAQ | Male: IPAQ total ST - including transport hours/day |  | - |
|  |  |  |  | TV *r* = 0.20*** | - |  |
|  |  |  |  | Computer games *r* = 0.06 | - |  |
|  |  |  |  | Sit listen to music *r* = 0.11* | - |  |
|  |  |  |  | Sit talk on telephone *r* = 0.17*** | - |  |
|  |  |  |  | Office/paperwork *r* = 0.31*** | - |  |
|  |  |  |  | Reading *r* = 0.02 | - |  |
|  |  |  |  | Playing musical instrument *r* = 0.00 | - |  |
|  |  |  |  | Arts and crafts *r* = 0.04 | - |  |
|  |  |  |  | Sitting driving in car = 0.19*** | - |  |
|  |  |  |  | Weekday (hours/day) *r* =0.24*** | - |  |
|  |  |  |  | Weekend (hours/day) *r* = 0.38*** | - |  |
|  |  |  |  | Total hours/week *r* = 0.31*** | - |  |
| SED-GIH^56^ | 284; 66,20 % women; 42.9 ± 8.9 yrs | Criterion | Inclinometer (activPAL3 micro), on leg, 7 days | SED-GIH answer categories and continuous activPAL-SIT for all days *rho* = 0.31 (95% CI= 0.20 to 0.41). | - | - |
|  |  |  |  | Agreement between the SED-GIH answer categories and categorized activPAL-SIT weighted *Kappa* = 0.12 (95% CI = 0.05 to 0.18) | - |  |
|  |  |  |  | 37.0% of the participants total estimated their sitting correctly, 40.8% underestimated their sitting and 22.2% overestimated their sitting. The 7–9 and 10–12 h spent sitting groups presented the highest numbers of correct estimations with 47.1 and 64.3%, respectively. participants who chose the answer ≤3 h in the SED-GIH question all underestimated their sitting time as compared to the categorical values of activPAL-SIT. Furthermore, nearly all participants who chose ≥13 h overestimated their sitting time compared to activPAL-SIT. |  |  |
| Workforce Sitting Questionnaire (WSQ)^57^ | 95; 63.2% women; 42.9 ± 8.9 yrs | Criterion | Accelerometer (Actigraph GT1M), on hip, activity counts were recorded in 10 seconds intervals  and aggregated into 1 min epochs | At work, workday *r* = 0.45 | - | - |
|  |  |  |  | Total, all domains, workday *r* = 0.34* | - |  |
|  |  |  |  | Total, all domains, non-workday *r* = 0.23* | - |  |
|  |  |  |  | Average total, work, and non-workday *r* = 0.40* | - |  |
|  |  |  |  | Bland Altman in average total sitting time between the WSQ and accelerometers: Mean difference = 44.55 mins/day, p<0.05); *LoA* = -295.31 to 384.41 min/day. |  |  |
|  |  |  |  | Bland Altman in sitting time at work on a workday when the WSQ was compared with accelerometers: mean difference 1.58 min/workday; p>0.05); *LoA* = -227.86 to 231.02 min/day |  |  |
| Japanese-Language Self-reported Measures for Assessing Adults Domain-Specific Sedentary Time (JSRM - SB)^58^ | 392; 60.2% women; 50.1 ± 7 yrs | Criterion | Accelerometer, (Active  style Pro), 7 days, 1-min epoch | Workday *rho* = 0.57** | - | - |
|  |  |  |  | Non-Workday *rho* = 0.23** | - |  |
|  |  |  |  | Whole week *rho* = 0.49** | - |  |
|  |  |  |  | Bland Altman for whole week: Mean difference = -13.4 min/day; *LoA* = -1.96 SD: -361.9 to 1.96 SG: 335.2 min/day |  |  |
| Longitudinal Aging Study Amsterdam questionnaire (LASA)^59^ | 83; 49.4% women; 64 - 92 yrs | Criterion | Accelerometer (Actigraph Model GT3X), on waist, 8 days | Total ST (10 items) *rho* = 0.35* | - | - |
|  |  |  |  | Total ST (6 items) *rho* = 0.46* | - |  |
|  |  |  |  | Bland Altman = Total sedentary time: Mean difference = 2.1 hours, with *LoA* of −7.40 to 3.25 hours |  |  |
| SB question of the Yale Physical Activity Survey (YPAS - SB)^60^ | 58; 79% female; 66 – 88 yr | Criterion | Accelerometer (Actigraph GT1M) on hip, 10 days, 1-minute epoch | Sitting time agreement YPAS and accelerometer = 8.6% |  | - |
|  |  |  |  | Sitting time k = −0.0003 (−0.0025 to 0.0019), (P trend < 0.01) | - |  |
| Community Health Activities Model Program for Seniors SB questions (CHAMPS - SB)^60^ | 58; 79% women; 66 – 88 yrs | Criterion | Accelerometer (Actigraph GT1M) on hip, 10 days, 1-minute epoch | Sitting time Lin’s concordance correlation coefficient = 0.005 (−0.010 to 0.020) | - | - |
|  |  |  |  | Sitting time *r* = 0.14; p-trend = 0.53 | - |  |
|  |  |  |  | Bland Altman: SB mean difference 5.21 hours/day *LoA* = 2.2 to 8.3 lower than accelerometer-derived |  | |
| Cancer Prevention Study-3 Sedentary Time Survey (CPS-3 sitting time)^61^ | 713; 59% women; 31 - 72 yrs | Criterion | Accelerometer (Actigraph GT3x), on hip, 7 days | Total ST *r* = 0.41 (95% CI: 0.35 to 0.47) | - | - |
| **Sleep** | | | | | | |
| BRFSS sleep questions (BRFSS Sleep)^62^ | 300; 68% women; 18 - 96 yrs | Criterion | Camntech Pro-Diary device with integrated activity monitor designed to collect patient reported outcome (subjective data) and actigraphy (objective data) simultaneously, 2 weeks, 1 minute epoch, on wrist | Bland Altman: the lack of agreement was 0.05 ± 1.2 (95% CI −2.1 to 2.2; considering the SD, 95% of people could report their total sleep time subjectively around 2 hours different from what would be measured objectively |  |  |
|  |  | Convergent | proposed question that the authors theorized: “How many  hours of sleep do you need to feel rested?” | Bland Altman: the lack of agreement is −0.41 ± 1.6 (95% CI to 3.5 to 2.7). The lack of agreement is overall around 25 min, and 95% of the population may report a sleep opportunity deficit of as much as 3.5 hours. |  |  |
| **Physical Activity + Sedentary Behaviour** | | | | | | |
| Kaiser Physical Activity Survey (KPAS)^63^ | 50; 100% women; 20-60 yrs | Criterion | Accelerometer (Caltrac), on Hip, 7 days | Caregiving score *rho* = 0.17 | - | - |
|  |  |  |  | Housework score *rho* = -0.03 | - |  |
|  |  |  |  | Housework/caregiving score *rho* = -0.01 | - |  |
|  |  |  |  | Sports/exercise *rho* = 0.57** | - |  |
|  |  |  |  | Active living habits *rho* = 0.34* | - |  |
|  |  |  |  | Occupation *rho* = 0.16 | - |  |
|  |  |  |  | 3-point summary *rho* = 0.53** | - |  |
|  |  |  |  | 4-point summary *rho* = 0.49** | - |  |
|  |  |  | Physical activity record | Caregiving score *rho* = 0.56** | - | - |
|  |  |  |  | Housework score *rho* = -0.64** | - |  |
|  |  |  |  | Housework/caregiving score *rho* = 0.67** | - |  |
|  |  |  |  | Sports/exercise *rho* = 0.73** | + |  |
|  |  |  |  | Active living habits *rho* = 0.22 | - |  |
|  |  |  |  | Occupation *rho* = 0.35* | - |  |
|  |  |  |  | 3-point summary *rho* = 0.42** | - |  |
|  |  |  |  | 4-point summary *rho* = 0.35* | - |  |
| Sedentary, Transportation and Activity Questionnaire (STAQ)^64^ | 96; 53.1% women; 40.5 ± 14.3 yrs |  | Accelerometer (Actigraph GT3X), on hip, 7 days, 100  counts per minutes | Sitting Time: |  | - |
|  |  |  |  | Total *ICC* = 0.44 (95% CI: 0.25 - 0.60) | - |  |
|  |  |  |  | Bland-Altman: With an important underestimation (−17 h/week) and wide *LoA* (−43.8 to 34.6 h/week) |  |  |
|  |  |  |  | Work *ICC* = 0.82 (95% CI: 0.73 to 0.88) | + |  |
|  |  |  |  | Transport *ICC* = 0.53 (95% CI: 0.35 to 0.67) | - |  |
|  |  |  |  | Leisure time *ICC* = 0.33 (95% CI: 0.12 to 0.51) | - |  |
|  |  |  |  | Specific sedentary behaviours: |  |  |
|  |  |  |  | Total *ICC* = 0.18 (95% CI: −0.04 to 0.38) | - |  |
|  |  |  |  | TV/DVD *ICC* = 0.34 (95% CI: 0.14 to 0.52) | - |  |
|  |  |  |  | Computer/tablet/video games *ICC* = 0.45 (95% CI: 0.26 to 0.60) | - |  |
|  |  |  | Contextualized logbook | Active transport: |  | - |
|  |  |  |  | Walking *ICC* = 0.38 (95% CI: 0.19 to 0.54) | - |  |
|  |  |  |  | Walking + cycling *ICC* = 0.38 (95% CI: 0.18 to 0.54) | - |  |
|  |  |  |  | Passive transport: |  |  |
|  |  |  |  | All transportation type *ICC* = 0.40 (95% CI: 0.21 to 0.56) | - |  |
|  |  |  |  | Car *ICC* = 0.66 (95% CI: 0.52 to 0.76) | - |  |
| International Physical Activity Questionnaire (IPAQ)^65^ | 26-151; N.R.; 18 - 65 yrs | Criterion | Accelerometer (CSA model 7164), 7 days, 1-min interval | Short form past week: |  | - |
|  |  |  |  | Physical activity Total *rho*= From 0.02 to 0 .47, depending on the country | - |  |
|  |  |  |  | ST *rho* = 0.12 - 0.49, depending on the country | - |  |
|  |  |  |  | Guideline’s compliance ACC = 0.46 to 0.93, depending on the country | + |  |
|  | 26-127; N.R.; 18 - 65 yrs | Criterion | Accelerometer (CSA model 7164), 7 days, 1-min interval | Short form usual week: |  | - |
|  |  |  |  | Physical activity total *rho*=- 0.12 to 0.32, depending on the country | - |  |
|  |  |  |  | ST *rho* = 0.07 to 0.26, depending on the country | - |  |
|  |  |  |  | Guideline’s compliance ACC= 0.50 to 0.75, depending on the country | + |  |
|  | 26 - 151; N.R.; 18-65 yrs | Criterion | Accelerometer (CSA model 7164), 7 days, 1-min interval | Long form past week: |  | - |
|  |  |  |  | Physical activity total *rho*= 0.05 to 0.52, depending on the country | - |  |
|  |  |  |  | ST *rho* = 0.25 to 0.51, depending on the country | - |  |
|  |  |  |  | Guideline’s compliance ACC = 0.31 to 1.0, depending on the country | + |  |
|  |  |  |  | Long form usual week: |  | - |
|  | 26 – 127; N.R.; 18-65 | Criterion | Accelerometer (CSA model 7164), 7 days, 1-min interval | Physical activity total *rho* = -0.27 to 0.36, depending on the country | - |  |
|  |  |  |  | Sitting *rho* = 0.14 to 0.22, depending on the country | - |  |
|  |  |  |  | Guideline’s compliance ACC = 0.35 to 0.72, depending on the country | + |  |
|  | 26 – 151; N.R.; 18-65 yrs | Convergent | Long form past week: | Short form past week |  | + |
|  |  |  |  | Total physical activity *rho* = 0.78 to 0.85, depending on the country | + |  |
|  |  |  |  | ST *rho* = 0.89 to 0.91, depending on the country | + |  |
|  |  |  |  | Guideline’s compliance ACC = 0.93 to 0.97, depending on the country | + |  |
|  | 26 - 127; N.R.; 18-65 yrs | Convergent | Long form usual week | Short form usual week |  | + |
|  |  |  |  | Total physical activity *rho* = 0.49 to 0.78, depending on the country | + |  |
|  |  |  |  | Sitting *rho* = 0.60 to 0.96, depending on the country | + |  |
|  |  |  |  | Guideline’s compliance ACC = 0.83 to 0.91, depending on the country | + |  |
| Australian Women’s Activity Survey (AWAS)^66^ | 75; 100% women; 32 ± 5 yrs | Criterion | Accelerometer (Actigraph) on waist, 7 days | Sitting Time *r* = 0.32* | - | - |
|  |  |  |  | LPA *r* = 0.12 | - |  |
|  |  |  |  | MPA *r* = 0.11 | - |  |
|  |  |  |  | VPA *r* = 0.07 | - |  |
|  |  |  |  | Total Activity *r* = 0.13 | - |  |
| Workers’ sitting- and walking-time questionnaire Time Method (WSWQ- t-method)^67^ | 64; 40% women; men mean age: 46.3 ± 8.0 yrs; women mean age: 35.8 ± 7.5 yrs | Criterion | Accelerometer (ActivPAL), on Leg, 7 days, 15 seconds intervals | Workday: |  |  |
|  |  |  |  | During working time: |  | - |
|  |  |  |  | Sitting *rho* = 0.52* | - |  |
|  |  |  |  | Walking/Standing *rho* = 0.56* | - |  |
|  |  |  |  | Sitting *kappa* = 0.39* | - |  |
|  |  |  |  | Walking/Standing *kappa* = 0.38* | - |  |
|  |  |  |  | During non-working time: |  |  |
|  |  |  |  | Sitting *rho* =0.55* | - |  |
|  |  |  |  | Walking/Standing *rho* = 0.58* | - |  |
|  |  |  |  | Sitting *kappa* = 0.43* | - |  |
|  |  |  |  | Walking/Standing *kappa* = 0.35* | - |  |
|  |  |  |  | Non-workday: |  |  |
|  |  |  |  | Sitting *rho* = 0.25 | - |  |
|  |  |  |  | Walking/Standing *rho* = 0.30* | - |  |
|  |  |  |  | Sitting *kappa* = 0.13 | - |  |
|  |  |  |  | Walking/Standing *kappa* = 0.16* | - |  |
|  |  |  |  | Bland Altman: mean differences at work = −6.7 mins/day (P = 0.68); Mean differences at non workday =−114.7 mins/day (P < 0.01); At work *LoA*=-254 to 241 mins/day; Non workday *LoA* = -588 to 358 mins/day |  |  |
| The Physical Activity Scale for the Elderly (PASE)^68^ | 222; N.R.; N.R. | Convergent | Perceived health | PASE activity score *r* = -0.25** | - | - |
|  |  |  | Sick Impact Profile | PASE activity score *r* = -0.42** | - |  |
|  |  |  | Grip Strength | PASE activity score *r* = 0.37** | - |  |
|  |  |  | Balance | PASE activity score *r* = 0.33** | - |  |
| Community Health Activities Model Program for Seniors physical activity self-report questionnaire + transport items (CHAMPS+transport)^69^ | 870; 50.7% women; over 80 yrs | Criterion | Accelerometer (Actigraph model 7164 and 71256) Waist, 7 days, epoch 1 minute | Sedentary duration *rho* = 0.12** | - | - |
|  |  |  |  | Low-LPA duration *rho* = 0.06 | - |  |
|  |  |  |  | High-LPA duration *rho* = 0.27** | - |  |
|  |  |  |  | MVPA duration *rho* = 0.37** | - |  |
|  |  |  |  | Total physical activity duration *rho* = 0.38** | - |  |
|  |  |  |  | MVPA caloric expenditure/ wk *rho* = 0.38** | - |  |
|  |  |  |  | Total physical activity caloric expenditure/ wk *rho* = 0.39** | - |  |
|  |  |  |  | Bland Altman: The CHAMPS indicated fewer minutes/week of sedentary (Mean difference = −2841.6 min/wk; 95% CI = −4476.7 to −1206.5 min/wk) and low-light activity (Mean difference = −472.7 min/wk; 95th CI = −1937.2 to 991.9 min/wk) and more minutes of high-light (Mean difference = 395.5 min/wk; 95% CI = −346.2 to 1137.2 min/wk), moderate-to-vigorous (Mean difference = 222.4 min/wk; 95% CI = −402.9 to 847.5 min/wk), and total activity (Mean difference = 617.8 min/wk; 95% CI = −504.1 to 1739.7min/wk) activity relative to accelerometery. |  |  |
| Community Healthy Activities Model Program for Seniors (CHAMPS)^70^ | 249; 63.9% women; 65 -90 yrs | Known-groups validity | Comparison of those initially inactive,a somewhat active,b and active | Caloric expenditure per week in at least moderate intensity physical activities F-test = F2,246 = 5 20.85** |  |  |
|  |  |  |  | Frequency per week in at least moderate intensity physical activities F-test = F2,246 = 38.93** |  |  |
|  |  |  |  | Caloric expenditure per week in all listed physical activities F-test = F2,246 = 17.80** |  |  |
|  |  |  |  | Frequency per week in all listed physical activities F-test = F2,246 = 29.26** |  |  |
|  |  | Convergent | 6-Min Walk | Caloric expenditure per week in at least moderate intensity  physical activities *r* = 0.27** | - | - |
|  |  |  |  | Frequency per week in at least moderate intensity physical activities *r* = 0.21** | - |  |
|  |  |  |  | Caloric expenditure per week in all listed physical activities *r* = 0.22** | - |  |
|  |  |  |  | Frequency per week in all listed physical activities *r* = 0.10 | - |  |
|  |  |  | Self-Reported Physical Functioning | Caloric expenditure per week in at least moderate intensity physical activities *r* = 0.30** | - | - |
|  |  |  |  | Frequency per week in at least moderate intensity physical activities *r* = 0.30** | - |  |
|  |  |  |  | Caloric expenditure per week in all listed physical activities *r* = 0.27** | - |  |
|  |  |  |  | Frequency per week in all listed physical activities *r* = 0.23** | - |  |
| Modified Version of the MONICA Optional Study on Physical Activity Questionnaire (Modified MOSPA-Q)^71^ | 70; N.R.; N.R. | Criterion |  | Walking *rho* = 0.27** | - | - |
|  |  |  |  | Lifting/Carrying NA (low prevalence) | - |  |
|  |  |  |  | Standing *rho* = 0.49* | - |  |
|  |  |  |  | Sitting *rho* = 0.52* | - |  |
| Occupational Sitting and Physical Activity Questionnaire (OSPAQ)^71,72^ | 76; N.R.; N.R. | Criterion | Accelerometer (ActiGraph GT1M), on Hip, 7 days, 10 seconds intervals and aggregated into 1-min epochs | Walking *rho* = 0.29** | - | - |
|  |  |  |  | Heavy Work NA (low prevalence) | - |  |
|  |  |  |  | Standing *rho* = 0.49* | - |  |
|  |  |  |  | Sitting *rho* = 0.65* | - |  |
|  |  |  |  | Bland-Altman: participants overestimated their occupational sitting time at high values and underestimated their occupational sitting at low values on the OSPAQ VS accelerometers. Differences were generally small (250- to 450-min). The mean difference between OSPAQ sitting time and accelerometer sedentary time at work = 22 min, 95% CI = 3 to 41 min). |  |  |
|  | 127; 82.7% women; 44.11 ± 11.16 yrs | Convergent | IPAQ | Walking *rho* = 0.43* (*LoA* -16.05 to 17.05%; Bland-Altman bias 0.50 % (SD8.44 %)) | - | - |
|  |  |  |  | Heavy Work N.R. | - |  |
|  |  |  |  | Standing *rho* = 0.56* (*LoA* -15.94 to 10.19%; Bland-Altman bias 2.87% (SD6.66 %)) | - |  |
|  |  |  |  | Sitting *rho* = 0.62* (*LoA* -20.37 to 25.16%; Bland-Altman bias 2.39 % (SD11.61%)) | - |  |
| Rapid Assessment Disuse Index (RADI)^73^ | 151; N.R.; N.R. | Criterion | Accelerometer (ActiGraph GT3X), on hip, 7 days, 60 min epoch | Cumulative RADI score: |  | - |
|  |  |  |  | ST *rho* = 0.402*** | - |  |
|  |  |  |  | Sedentary breaks *rho* = -0.425** | - |  |
|  |  |  |  | LPA *rho* = -0.406** | - |  |
|  |  |  |  | MVPA *rho* = -0.063 |  |  |
|  |  |  |  | Distinction between high sedentary and low sedentary individuals: AUC = 0.72 | + |  |
|  |  |  |  | RADI score of 27 resulted in a sensitivity of 0.60 and specificity of 0.74; a score of 26 resulted in sensitivity 0.79 and specificity 0.63, and when the RADI score was 25, the sensitivity remained constant (0.79) and specificity decreased (0.59). | - |  |
| Global Physical Activity Questionnaire (GPAQ)^74^ | 148; N.R.; N.R. | Convergent | IPAQ | Total vigorous *rho* = 0.68** | - | - |
|  |  |  |  | Total moderate *rho* = 0.68** | - |  |
|  |  |  |  | Total physical activity *rho* = 0.29** | - |  |
|  |  |  |  | Sedentary time *rho* = 0.68** | - |  |
|  |  |  |  | inactive *Kappa* = 0.52 (89.2%) | - |  |
|  |  | Criterion | Pedometer | Total physical activity time *rho* = 0.23** | - | - |
|  |  |  |  | Total sedentary time *rho* = 0.00 | - |  |
| **Sedentary Behaviour + Sleep** | | | | | | |
| SIT-Q^75^ | 69; N.R.; N.R. | Convergent | Seven-Day Activity Diary | Meals *rho* = 0.29* | - | - |
|  |  |  |  | Transport *rho* = 0.34** | - |  |
|  |  |  |  | Occup *rho* = 0.75** | + |  |
|  |  |  |  | Child and elder care *rho* = 0.46** | - |  |
|  |  |  |  | Leisure Time *rho* = 0.26* | - |  |
|  |  |  |  | Total sitting time *rho* = 0.52** | - |  |
| **Physical Activity + Sedentary Behaviour + Sleep** | | | | | | |
| Physical Activity Questionnaire (PAQ)^48^ | 111 men; N.R. | Convergent | Self-administered structured 7-day physical activity diary | Crude Total physical activity *rho* = 0.22* | - | - |
|  |  |  |  | Total activity score *rho* = 0.53* | - |  |
|  |  |  |  | work/occupation *rho* = 0.38* | - |  |
|  |  |  |  | home/household *rho* = 0.58* | - |  |
|  |  |  |  | activity leisure-time *rho* = 0.37* | - |  |
|  |  |  |  | inactive leisure (TV/reading) *rho*=0.49* | - |  |
|  |  |  |  | sleep *rho*=0.57* | - |  |
| Athens Physical Activity Questionnaire (APAQ)^77^ | 79; 53% Women; 28 ± 6 year | Criterion | Accelerometer (RT3 Tri-axial Research Tracker) | Total energy expenditure *rho* = 0.84 (0.44 and 0.63 for both genders, women and men, respectively (all P < 0.01)). | + | + |
|  |  |  |  | Bland-Altman plotting showed a good agreement between APAQ and RT3 - differences within the 95% *LoA*. Furthermore, the lack of significant slope (*r* < 0.14, P > 0.385) indicated no proportional bias in energy expenditure derived from the APAQ. |  |  |
| Sedentary Time and Activity Reporting Questionnaire (STAR-Q)^78^ | 99; N.R.; N.R. | Convergent | 7-day activity diary | Total energy expenditure, kcal/day *rho* = 0.74** | + | - |
|  |  |  |  | AEE, kcal/day *rho* = 0.61** | - |  |
|  |  |  |  | AEE, kcal/kg.day *rho* = 0.45** | - |  |
|  |  |  |  | Sleeping *rho*= 0.62** | - |  |
|  |  |  |  | Stair-climbing, flights/day *rho* = 0.41** | - |  |
|  |  |  |  | Active sitting *rho* = 0.30* | - |  |
|  |  |  |  | Overall Activity: |  |  |
|  |  |  |  | SB *rho* = 0.40** | - |  |
|  |  |  |  | Light intensity *rho* = 0.29 | - |  |
|  |  |  |  | Mod intensity *rho* = 0.57** | - |  |
|  |  |  |  | Vig intensity *rho* = 0.68** | - |  |
|  |  |  |  | Exercise, sports, and  leisure activity |  |  |
|  |  |  |  | General = 0.47** | - |  |
|  |  |  |  | Light intensity *rho* = 0.21* | - |  |
|  |  |  |  | Mod intensity *rho* = 0.35** | - |  |
|  |  |  |  | Vig intensity *rho* = 0.49** | - |  |
|  |  |  |  | Occup activity: |  |  |
|  |  |  |  | General *rho* = 0.71** | + |  |
|  |  |  |  | Sitting *rho* = 0.75** | + |  |
|  |  |  |  | SB *rho* = 0.76** | + |  |
|  |  |  |  | Light intensity *rho* = 0.24* | - |  |
|  |  |  |  | Mod intensity *rho* = 0.30* | - |  |
| Question 8 of the Paffenbarger Physical Activity Questionnaire (Q 8 PPAQ)^79^ | 419; 50.8% women; men age: 43.8 ± 15.8 yrs; Women age: 44.3 ± 16.5 yrs | Criterion | Accelerometer, on hip, 25-second epochs, 4 consecutive days 2 days during the week and 2 days over the weekend | Light intensity *rho* = 0.02 (95% CI: –0.09 - 0.11) | - | - |
|  |  |  |  | Moderate intensity *rho* = 0.15** (95% CI: 0.11 to 0.30) | - |  |
|  |  |  |  | Vigorous intensity *rho* = 0.20** (95% CI: –0.01 to 0.19) | - |  |
|  |  |  |  | Sedentary Time *rho* = 0.20** (95% CI: 0.14 to 0.33) | - |  |
|  |  |  |  | METs light intensity = 0.26 (95% CI: 0.20 to 0.40) | - |  |
|  |  |  |  | METs moderate intensity = 0.17** (95% CI: 0.13 - 0.33) | - |  |
|  |  |  |  | METs vigorous intensity = –0.01 (95% CI: –0.13 to 0.07) | - |  |
|  |  |  |  | METs SB = –.24** (95% CI: –0.39 to 0.20) | - |  |
| EPIC-Norfolk Physical Activity Questionnaire (EPAQ2)^80^ | 173; 51.45% women; men age 58.8 ± 7.9 yrs; women age 55.4 ± 6.7 yrs | Criterion | Heart rate monitoring (kJ/h) | TV *rho* = -0.7 | - | - |
|  |  |  |  | Activity at home *rho* = -0.04 | - |  |
|  |  |  |  | Activity at work *rho* = 0.17* | - |  |
|  |  |  |  | Recreational activity *rho* = 0.13 | - |  |
|  |  |  |  | VPA *rho* = 0.01 | - |  |
|  |  |  |  | Physical activity index *rho* = 0.28** | - |  |
|  |  | Convergent | VO2max | TV *rho* = -019* | - | - |
|  |  |  |  | Activity at home *rho* = -0.09 | - |  |
|  |  |  |  | Activity at work *rho* = 0.01 | - |  |
|  |  |  |  | Recreational activity *rho* = 0.16* | - |  |
|  |  |  |  | VPA *rho* = 0.16* | - |  |
|  |  |  |  | Physical activity index *rho* = 0.15* | - |  |
| Workers’ sitting- and walking-time questionnaire Percentage Method (WSWQ - p-method)^67^ | 64; 40% female; men age: 46.3 ± 8.0 yrs; women age: 35.8 ± 7.5 yrs | Criterion | Accelerometer (ActivPAL), on Leg, 7-days, 15-s intervals | Workday: |  | - |
|  |  |  |  | During working time: |  |  |
|  |  |  |  | Sitting *rho* = 0.59* | - |  |
|  |  |  |  | Walking/Standing *rho* = 0.56* | - |  |
|  |  |  |  | Sitting K = 0.44* | - |  |
|  |  |  |  | Walking/Standing k = 0.35* | - |  |
|  |  |  |  | During non-working time: |  |  |
|  |  |  |  | Sitting *rho* = 0.57* | - |  |
|  |  |  |  | Walking/Standing *rho* = 0.61* | - |  |
|  |  |  |  | Sitting K = 0.36* | - |  |
|  |  |  |  | Walking/Standing k = 0.40* | - |  |
|  |  |  |  | Non-workday: |  |  |
|  |  |  |  | Sitting *rho* = 0.42* | - |  |
|  |  |  |  | Walking/Standing *rho* = 0.45* | - |  |
|  |  |  |  | Sitting K = 0.23* | - |  |
|  |  |  |  | Walking/Standing k = 0.21* | - |  |
|  |  |  |  | Bland Altman: mean differences = 34.5 min/day (P = 0.03); Mean differences at non workday =−55.7 min/day  (P = 0.02); At work *LoA*=-200 - 269 min/day; Non workday *LoA* =-392 - 281 min/day |  |  |
| New Questionnaire on Physical Activity (NQPA)^81^ | 111; 46.85% women; <50yrs = 56.76%; >=50 yrs = 43.24% | Convergent | Physical activity Diary | Men: |  | - |
|  |  |  |  | 1st questionnaire *r* = 0.66 (95% CI: 0.49 to 0.78) | - |  |
|  |  |  |  | Women: |  |  |
|  |  |  |  | 1st questionnaire *r* = 0.43 (0.18–0.63) | - |  |
|  |  |  |  | Bland-Altman: The limits of 2 standard deviations below and above the mean difference were −3368 and 3243 kJ. This means that for 95% of the subjects the outcome of the second questionnaire fell within an interval of 3368 kJ below and 3243 kJ above the outcome of the first. |  |  |
| Web-Based Physical Activity Questionnaire (Active-Q)^82^ | 148 Men; 33 - 86 yrs | Criterion | Accelerometer (GENEA), on wrist, 6 days, 1-minute epoch | SB *r* = 0.19 (95% CI: 0.04 to 0.34) | - | - |
|  |  |  |  | LPA *r* = 0.15 (95% CI: 0.00 to 0.31) | - |  |
|  |  |  |  | SB+LPA = 0.35 (95% CI: 0.19 to 0.51) | - |  |
|  |  |  |  | MPA *r* = 0.27 (95% CI: 0.12 to 0.42) | - |  |
|  |  |  |  | VPA *r* = 0.54 (95% CI: 0.42 to 0.67) | - |  |
|  |  |  |  | MVPA *r* = 0.35 (95% CI: 0.21 to 0.48) | - |  |
|  |  |  |  | Bland Altman: SB: mean difference = -178, 95%; *LoA*= -606 – 250 mins/day |  |  |
|  |  |  |  | Bland Altman: LPA: mean difference=-178, 95% *LoA*=-606 to 250 mins/day |  |  |
|  |  |  |  | Bland Altman: SB+LPA: mean difference=-329, 95% *LoA*=-329 to 146 mins/day |  |  |
|  |  |  |  | Bland Altman: MPA: mean difference=76, 95% *LoA*=-157 to 309 mins/day |  |  |
|  |  |  |  | Bland Altman: VPA: mean difference=15, 95% *LoA*=-33 to 64 mins/day |  |  |
|  |  |  |  | Bland Altman: MVPA: mean difference=91, 95% *LoA*=-147 to 329 mins/day |  |  |
| Flemish Physical Activity Computerized Questionnaire (FPACQ)^83^ | 62; 32 men: 39.28 ± 11.72 yrs; 30 women: 39.13 ± 11.96 yrs | Criterion | Accelerometer (RT3 Triaxial Research Tracker, StayHealthy, Inc., Monrovia, CA), on hip, all 1-min epochs over 7 days | Employed/Unemployed Men |  | - |
|  |  |  |  | Time/week spent on sports participation *r* = 0.77*** | + |  |
|  |  |  |  | Energy expenditure/week on sports participation *r* = 0.47** | - |  |
|  |  |  |  | Average energy expenditure on sports participation *r* = 0.54** | - |  |
|  |  |  |  | Time/week spent eating *r* = 0.53** | - |  |
|  |  |  |  | Time/week spent sleeping *r* = 0.69*** | - |  |
|  |  |  |  | Time/week spent watching television or videos or playing computer games *r* = 0.69*** | - |  |
|  |  |  |  | Time/week spent on leisure-time active transportation *r* = 0.55** |  |  |
|  |  |  |  | Time/week spent on active leisure-time activities *r* = 0.35 | - |  |
|  |  |  |  | Energy expenditure/week on active leisure time-activities *r* = 0.37* | - |  |
|  |  |  |  | Average energy expenditure on active leisure-time activities *r* = 0.51** | - |  |
|  |  |  |  | Time/week spent on occupation and transportation to and from occupation *r* = 0.78*** | + |  |
|  |  |  |  | Energy expenditure/week on occupation and transportation  to and from occupation *r* = 0.85*** | + |  |
|  |  |  |  | Average energy expenditure on occupation and transportation to and from occupation *r* = 0.84*** | + |  |
|  |  |  |  | Overall energy expenditure during a usual week *r* = 0.80*** | + |  |
|  |  |  |  | Physical activity level (MET) *r* = 0.56** | - |  |
|  |  |  |  | Employed/Unemployed Women |  |  |
|  |  |  |  | Time/week spent on sports participation *r* = 0.63*** | - |  |
|  |  |  |  | Energy expenditure/week on sports participation *r* = 0.67*** | - |  |
|  |  |  |  | Average energy expenditure on sports participation *r* = 0.27 | - |  |
|  |  |  |  | Time/week spent eating *r* = 0.56** | - |  |
|  |  |  |  | Time/week spent sleeping *r* = 0.60*** | - |  |
|  |  |  |  | Time/week spent watching television or videos or playing computer games *r* = 0.83*** | + |  |
|  |  |  |  | Time/week spent on leisure-time active transportation *r* = 0.49** | - |  |
|  |  |  |  | Time/week spent on active leisure-time activities *r* = 0.75*** | + |  |
|  |  |  |  | Energy expenditure/week on active leisure time-activities *r* = 0.67*** | - |  |
|  |  |  |  | Average energy expenditure on active leisure-time activities *r* = 0.84*** | + |  |
|  |  |  |  | Time/week spent on occupation and transportation to and from occupation *r* = 0.88*** | + |  |
|  |  |  |  | Energy expenditure/week on occupation and transportation  to and from occupation *r* = 0.83*** | + |  |
|  |  |  |  | Average energy expenditure on occupation and transportation to and from occupation *r* = 0.78*** | + |  |
|  |  |  |  | Overall energy expenditure during a usual week *r* = 0.65*** | - |  |
|  |  |  |  | Physical activity level (MET) *r* = 0.44* | - |  |
|  | 49; 30 men: 64.47 ± 5.48 yrs; 19 women: 65.37 ± 4.98 yrs |  |  | Retired Men |  |  |
|  |  |  |  | Time/week spent on sports participation *r* = 0.66*** | - |  |
|  |  |  |  | Energy expenditure/week on sports participation *r* = 0.37 | - |  |
|  |  |  |  | Average energy expenditure on sports participation *r* = 0.25 | - |  |
|  |  |  |  | Time/week spent eating *r* = 0.33 | - |  |
|  |  |  |  | Time/week spent sleeping *r* = 0.57** | - |  |
|  |  |  |  | Time/week spent watching television or videos or playing computer games *r* = 0.78*** | + |  |
|  |  |  |  | Time/week spent on leisure-time active transportation *r* = 0.55** | - |  |
|  |  |  |  | Time/week spent on active leisure-time activities *r* = 0.33 | - |  |
|  |  |  |  | Energy expenditure/week on active leisure time-activities *r* = 0.39* | - |  |
|  |  |  |  | Average energy expenditure on occupation and transportation to and from occupation *r* = 0.41* | - |  |
|  |  |  |  | Overall energy expenditure during a usual week *r* = 0.55** | - |  |
|  |  |  |  | Physical activity level (MET) *r* = 0.39* | - |  |
|  |  |  |  | Retired Women |  |  |
|  |  |  |  | Time/week spent on sports participation *r* = 0.38 | - |  |
|  |  |  |  | Energy expenditure/week on sports participation *r* = 0.51* | - |  |
|  |  |  |  | Average energy expenditure on sports participation *r* = 0.23 | - |  |
|  |  |  |  | Time/week spent eating *r* = 0.15 | - |  |
|  |  |  |  | Time/week spent sleeping *r* = 0.51* | - |  |
|  |  |  |  | Time/week spent watching television or videos or playing computer games *r* = 0.80*** | + |  |
|  |  |  |  | Time/week spent on leisure-time active transportation *r* = 0.52* | - |  |
|  |  |  |  | Time/week spent on active leisure-time activities *r* = 0.28 | - |  |
|  |  |  |  | Energy expenditure/week on active leisure time-activities *r* = 0.44 | - |  |
|  |  |  |  | Average energy expenditure on occupation and transportation to and from occupation *r* = 0.64** | - |  |
|  |  |  |  | Overall energy expenditure during a usual week *r* = 0.85*** | + |  |
|  |  |  |  | Physical activity level (MET) *r* = 0.50* | - |  |

Abbreviations: n= Sample Number; SD= Standard Deviation; yrs= years; CI=Confidence interval; VPA= Vigorous Physical Activity; MPA= Moderate Physical Activity; MVPA= Moderate-Vigorous Physical Activity; MET= Metabolic Equivalent Task; ACC= average correct classification; * p≤0.05; ** p≤0.001;***p≤0.0001; + = Adequate; - = Inadequate;

**References**

30. Kurtze N, Rangul V, Hustvedt B, Flanders WD. Reliability and validity of self-reported physical activity in the Nord-Trøndelag Health Study -- HUNT 1. Scandinavian Journal of Public Health. 2008;36(1):52-61. doi:10.1177/1403494807085373

31. Friedenreich CM, Courneya KS, Neilson HK, et al. Reliability and validity of the Past Year Total Physical Activity Questionnaire. American Journal of Epidemiology. 2006;163(10):959-970. doi:aje/kwj112

32. Meriwether RA, McMahon PM, Islam N, Steinmann WC. Physical Activity Assessment: Validation of a Clinical Assessment Tool. American Journal of Preventive Medicine. 2006;31(6):484-491. doi:10.1016/j.amepre.2006.08.021

33. Jacobs DR, Ainsworth BE, Hartman TJ, Leon AS. A simultaneous evaluation of 10 commonly used physical activity questionnaires. / Evaluation simultanee de 10 questionnaires couramment utilises sur les activites physiques. Medicine & Science in Sports & Exercise. 1993;25(1):81-91.

34. Milton K, Bull FC, Bauman A. Reliability and validity testing of a single-item physical activity measure. British Journal of Sports Medicine. 2011;45(3):203-208.

35. Chasean-Taber L, Erickson JB, Nasca PC, Chasan-Taber S, Freedson PS. Validity and reproducibility of a physical activity questionnaire in women. / Validite et reproductibilite d ' un questionnaire sur l ' activite physique chez des femmes. Medicine & Science in Sports & Exercise. 2002;34(6):987-992.

36. Brown WJ, Burton NW, Marshall AL, Miller YD. Reliability and validity of a modified self-administered version of the Active Australia physical activity survey in a sample of mid-age women. Australian & New Zealand Journal of Public Health. 2008;32(6):535-541.

37. Fjeldsoe BS, Winkler EAH, Marshall AL, Eakin EG, Reeves MM. Active adults recall their physical activity differently to less active adults: test-retest reliability and validity of a physical activity survey. Health Promotion Journal of Australia. 2013;24(1):26-31. doi:10.1071/HE12912

38. Timperio A, Salmon J, Crawford D. Validity and reliability of a physical activity recall instrument among overweight and non-overweight men and women. Journal of Science & Medicine in Sport. 2003;6(4):477-491.

39. van der Ploeg HP, Tudor-Locke C, Marshall AL, et al. Reliability and validity of the international physical activity questionnaire for assessing walking. Res Q Exerc Sport. Mar 2010;81(1):97-101. doi:10.1080/02701367.2010.10599632

40. Wendel-Vos GW, Schuit AJ, Saris WH, Kromhout D. Reproducibility and relative validity of the short questionnaire to assess health-enhancing physical activity. Journal of clinical epidemiology. 2003;56(12):1163-1169.

41. Cust AE, Smith BJ, Chau J, et al. Validity and repeatability of the EPIC Physical Activity Questionnaire: A validation study using accelerometers as an objective measure. The international journal of behavioral nutrition and physical activity. 2008;5

42. Nikolaidis PT, Säcklova M. Validity against health-related fitness and reliability of physical activity questionnaire in young female and male adults. Journal of Physical Education & Sport. 2011;11(3):342-348.

43. de Souto Barreto P. Construct and convergent validity and repeatability of the Questionnaire d’Activité Physique pour les Personnes Âgées (QAPPA), a physical activity questionnaire for the elderly. Public Health. 2013;127(9):844-853. doi:10.1016/j.puhe.2012.10.018

44. Delbaere K. Evaluation of the incidental and planned activity questionnaire for older people. British Journal of Sports Medicine. 2010;44(14):1029-1034.

45. Yasunaga A, Park H, Watanabe E, et al. Development and evaluation of the physical activity questionnaire for elderly Japanese: The Nakanojo study. Journal of Aging and Physical Activity. 2007;15(4):398-411. doi:10.1123/japa.15.4.398

46. Siebeling L, Wiebers S, Beem L, Puhan MA, Ter Riet G. Validity and reproducibility of a physical activity questionnaire for older adults: questionnaire versus accelerometer for assessing physical activity in older adults. Clinical epidemiology. 2012;4:171.

47. Danquah IH, Petersen CB, Skov SS, Tolstrup JS. Validation of the NPAQ-short - a brief questionnaire to monitor physical activity and compliance with the WHO recommendations. BMC Public Health. 2018;18(1):N.PAG-N.PAG. doi:10.1186/s12889-018-5538-y

48. Visuthipanich V, Sirapo-ngam Y, Malathum P, Kijboonchoo K, Vorapongsathorn T, Winters-Stone K. Physical activity questionnaire development and testing among elderly community-dwelling Thais. Thai Journal of Nursing Research. 2009;13(4):249-267.

49. Adams EJ, Goad M, Sahlqvist S, Bull FC, Cooper AR, Ogilvie D. Reliability and validity of the Transport and Physical Activity Questionnaire (TPAQ) for assessing physical activity behaviour. PLoS ONE. 2014;9(9)

50. Ahmad S, Harris T, Limb E, et al. Evaluation of reliability and validity of the General Practice Physical Activity Questionnaire (GPPAQ) in 60–74 year old primary care patients. BMC family practice. 2015;16(1):113.

51. Rosenberg DE, Bull FC, Marshall AL, Sallis JF, Bauman AE. Assessment of sedentary behavior with the International Physical Activity Questionnaire. Journal of Physical Activity & Health. 2008;5(Suppl1):S30-S44.

52. Marshall AL, Miller YD, Burton NW, Brown WJ. Measuring total and domain-specific sitting: a study of reliability and validity. Med Sci Sports Exerc. Jun 2010;42(6):1094-102. doi:10.1249/MSS.0b013e3181c5ec18

53. Sudholz B, Ridgers ND, Mussap A, Bennie J, Timperio A, Salmon J. Reliability and validity of self-reported sitting and breaks from sitting in the workplace. J Sci Med Sport. Jul 2018;21(7):697-701. doi:10.1016/j.jsams.2017.10.030

54. Pedisic Z, Bennie JA, Timperio AF, et al. Workplace Sitting Breaks Questionnaire (SITBRQ): an assessment of concurrent validity and test-retest reliability. BMC Public Health. Dec 5 2014;14:1249. doi:10.1186/1471-2458-14-1249

55. Rosenberg DE, Norman GJ, Wagner N, Patrick K, Calfas KJ, Sallis JF. Reliability and validity of the Sedentary Behavior Questionnaire (SBQ) for adults. Journal of Physical Activity & Health. 2010;7(6):697-705. doi:10.1123/jpah.7.6.697

56. Larsson K, Kallings LV, Ekblom Ö, Blom V, Andersson E, Ekblom MM. Criterion validity and test-retest reliability of SED-GIH, a single item question for assessment of daily sitting time. BMC public health. 2019;19(1):17.

57. Chau JY, Van Der Ploeg HP, Dunn S, Kurko J, Bauman AE. A tool for measuring workers' sitting time by domain: the Workforce Sitting Questionnaire. British journal of sports medicine. 2011;45(15):1216-1222.

58. Ishii K, Shibata A, Kurita S, et al. Validity and reliability of Japanese-language self-reported measures for assessing adults domain-specific sedentary time. Journal of epidemiology. 2017:JE20170002.

59. Visser M, Koster A. Development of a questionnaire to assess sedentary time in older persons–a comparative study using accelerometry. BMC geriatrics. 2013;13(1):80.

60. Gennuso KP, Matthews CE, Colbert LH. Reliability and validity of 2 self-report measures to assess sedentary behavior in older adults. Journal of Physical Activity & Health. 2015;12(5):727-732. doi:10.1123/jpah.2013-0546

61. Rees-Punia E, Matthews CE, Evans EM, et al. Demographic-specific validity of the cancer prevention study-3 sedentary time survey. Medicine and science in sports and exercise. 2019;51(1):41.

62. Jungquist CR, Mund J, Aquilina AT, et al. Validation of the Behavioral Risk Factor Surveillance System Sleep Questions. J Clin Sleep Med. Mar 2016;12(3):301-10. doi:10.5664/jcsm.5570

63. Ainsworth BE, Sternfeld B, Richardson MT, Jackson K. Evaluation of the Kaiser Physical Activity Survey in women. Medicine & Science in Sports & Exercise. 2000;32(7):1327-1334.

64. Mensah K, Maire A, Oppert J-M, et al. Assessment of sedentary behaviors and transport-related activities by questionnaire: a validation study. BMC Public Health. 2016;16(1):1-9. doi:10.1186/s12889-016-3412-3

65. Craig CL, Marshall AL, Sjöström M, et al. International physical activity questionnaire: 12-country reliability and validity. Med Sci Sports Exerc. Aug 2003;35(8):1381-95. doi:10.1249/01.mss.0000078924.61453.fb

66. Fjeldsoe BS, Marshall AL, Miller YD. Measurement properties of the Australian Women's Activity Survey. Medicine & Science in Sports & Exercise. 2009;41(5):1020-1033. doi:10.1249/MSS.0b013e31819461c2

67. Matsuo T, Sasai H, So R, Ohkawara K. Percentage-method improves properties of workers’ sitting-and walking-time questionnaire. Journal of epidemiology. 2016;26(8):405-412.

68. Washburn RA, Smith KW, Jette AM, Janney CA. The Physical Activity Scale for the Elderly (PASE): Development and evaluation. Journal of Clinical Epidemiology. 1993;46(2):153-162. doi:10.1016/0895-4356(93)90053-4

69. Hekler EB, Buman MP, Haskell WL, et al. Reliability and validity of CHAMPS self-reported sedentary-to-vigorous intensity physical activity in older adults. Journal of Physical Activity and Health. 2012;9(2):225-236.

70. Stewart AL, Mills KM, King AC, Haskell WL, Gillis D, Ritter PL. CHAMPS physical activity questionnaire for older adults: outcomes for interventions. / CHAMPS: Questionnaire sur les activites physiques des personnes agees: resultats pour de futures interventions. Medicine & Science in Sports & Exercise. 2001;33(7):1126-1141.

71. Chau JY, Van Der Ploeg HP, Dunn S, Kurko J, Bauman AE. Validity of the Occupational Sitting and Physical Activity Questionnaire. Medicine & Science in Sports & Exercise. 2012;44(1):118-125.

72. Pedersen SJ, Kitic CM, Bird M-L, Mainsbridge CP, Cooley PD. Is self-reporting workplace activity worthwhile? Validity and reliability of occupational sitting and physical activity questionnaire in desk-based workers. BMC Public Health. 2016;16(1):836-836. doi:10.1186/s12889-016-3537-4

73. Shuval K, Harold WK, III, Bernstein I, et al. Sedentary behaviour and physical inactivity assessment in primary care: the Rapid Assessment Disuse Index (RADI) study. British Journal of Sports Medicine. 2014;48(3):250-255.

74. Bull FC, Maslin TS, Armstrong T. Global Physical Activity Questionnaire (GPAQ): Nine country reliability and validity study. Journal of Physical Activity & Health. 2009;6(6):790-804.

75. Lynch BM, Friedenreich CM, Khandwala F, Liu A, Nicholas J, Csizmadi I. Development and testing of a past year measure of sedentary behavior: the SIT-Q. BMC Public Health. Sep 1 2014;14:899. doi:10.1186/1471-2458-14-899

76. Norman A, Bellocco R, Bergström A, Wolk A. Validity and reproducibility of self-reported total physical activity--differences by relative weight. Int J Obes Relat Metab Disord. May 2001;25(5):682-8. doi:10.1038/sj.ijo.0801597

77. Kavouras SA, Maraki MI, Kollia M, Gioxari A, Jansen LT, Sidossis LS. Development, reliability and validity of a physical activity questionnaire for estimating energy expenditure in Greek adults. Science & Sports. 2016;31(3):e47-e53.

78. Csizmadi I, Neilson HK, Kopciuk KA, et al. The Sedentary Time and Activity Reporting Questionnaire (STAR-Q): reliability and validity against doubly labeled water and 7-day activity diaries. American journal of epidemiology. 2014;180(4):424-435.

79. Simpson K, Parker B, Capizzi J, et al. Validity and Reliability of Question 8 of the Paffenbarger Physical Activity Questionnaire Among Healthy Adults. Journal of Physical Activity & Health. 2015;12(1):116-123.

80. Wareham NJ, Jakes RW, Rennie KL, Mitchell J, Hennings S, Day NE. Validity and repeatability of the EPIC-Norfolk physical activity questionnaire. International journal of epidemiology. 2002;31(1):168-174.

81. Pols MA, Peeters PH, Ocke MC, et al. Relative validity and repeatability of a new questionnaire on physical activity. Preventive Medicine. 1997;26(1):37-43.

82. Bonn SE, Bergman P, Lagerros YT, Sjölander A, Bälter K. A validation study of the web-based physical activity questionnaire active-Q against the GENEA accelerometer. JMIR research protocols. 2015;4(3):e86.

83. Matton L, Wijndaele K, Duvigneaud N, et al. Reliability and Validity of the Flemish Physical Activity Computerized Questionnaire in Adults. Research Quarterly for Exercise & Sport. 2007;78(4):293-306.
