# Supplementary material for: Questionnaires measuring movement behaviours in adults and older adults: Content description and measurement properties. A systematic review
Source: PLoS One. 2022 Mar 11;17(3):e0265100. doi: 10.1371/journal.pone.0265100 (PMC8916622; doi:10.1371/journal.pone.0265100)
Supplement: S3 Table — (DOCX) [file pone.0265100.s006.docx]

**Supporting table 3 - Reliability results**

| **Questionnaire** | Sample | | Reliability | Quality of reliability results | Overall Quality |
| --- | --- | --- | --- | --- | --- |
|  | n; % Women; Age mean ± SD or Age range (years) | Time between test and retest | Results |  |  |
| **Physical Activity** | | | | | |
| Nord-Trøndelag Health Study PA Questionnaire (HUNT 1)^30^ | 102; N.R.; N.R. | 1 week | Frequency *rho* = 0.87** | + | + |
|  |  |  | Intensity: *rho* = 0.87** | + |  |
|  |  |  | Duration: *rho* = 0.78** | - |  |
|  |  |  | Frequency Weighted *kappa* = 0.80 | + |  |
|  |  |  | Intensity Weighted *k11appa* = 0.82 | + |  |
|  |  |  | Duration Weighted kappa = 0.69 | - |  |
| Past Year Total Physical Activity Questionnaire (PYTPAQ)^31^ | 154; 51% women; 48.7 ± 7.7 | 9 weeks | Total physical activity *ICC* = 0.66 (95% CI: 0.56 to 0.74) | - | - |
|  |  |  | VPA *ICC* = 0.72 (95% CI: 0.56 to 0.83) | + |  |
|  |  |  | LPA /MPA *ICC* = 0.55 (95% CI: 0.40 to 0.67) | - |  |
|  |  |  | Occup *ICC* = 0.58 (95% CI: 0.47 to 0.68) | - |  |
| Physical Activity Assessment Tool (PAAT)^32^ | 68; N.R.; N.R. | 1 week | Total physical activity *r* = 0.62*** | - | - |
|  |  |  | MPA *r* = 0.49*** | - |  |
|  |  |  | VPA *r* = 0.77`*** | + |  |
| Minnesota Leisure Time Physical Activity Questionnaire (Minnesota LTPA Q)^33^ | 78; 64.1% women; 20-59 years | 1 month | Total *r* = 0.92 | + | + |
|  |  |  | LPA *r* = 0.73 | - |  |
|  |  |  | MPA *r* = 0.80 | + |  |
|  |  |  | Vig *r* = 0.95 | + |  |
|  |  |  | Household chores *r* = 0.88 | + |  |
| Single Item Physical Activity Measure (SI PA M)^34^ | 240; 50% women; 18-64 years | 2 to 5 days | Physical activity wk *r* = 0.72 | - | - |
|  |  |  | Physical activity month *r* = 0.86 | + |  |
|  |  |  | Agreement categorical variables: Weighted *kappa* = 0.82 (95% CI: 0.69 to 0.82) | + |  |
| Godin Questionnaire (Godin Q)^33^ | 77; N.R.; N.R. | 1 month | Leisure score *r* = 0.62 | - | - |
|  |  |  | LPA *r* = 0.24 | - |  |
|  |  |  | MPA *r* = 0.36 | - |  |
|  |  |  | VPA *r* = 0.84 | - |  |
| CARDIA Physical Activity History (CARDIA)^33^ | 78; 64.1% women; 20-59 years | 1 month | Total physical activity *r* = 0.88 | + | + |
|  |  |  | MPA *r* = 0.66 | - |  |
|  |  |  | Vig *r* = 0.91 | + |  |
| College Alumnus Questionnaire (College Alumnus Q)^33^ | 58; N.R.; N.R. | 1 month | Total index *r* = 0.72 | - | - |
|  |  |  | Stairs *r* = -0.78 | - |  |
|  |  |  | Blocks *r* = 0.63 | - |  |
|  |  |  | Sports *r* = 0.75 | - |  |
| Minnesota Heart Health Program Questionnaire (MHHP Q)^33^ | 77; N.R.; N.R. | 1 month | Work index *r* = 0.91 | + | + |
|  |  |  | Leisure index *r* = 0.86 | + |  |
| Modified Historical Leisure Activity Questionnaire (MHLAQ)^35^ | 131; N.R.; 50.4 ± 7.6 years | 1 yr | Total physical activity *ICC* = 0.82** | + | + |
|  |  |  | MPA *ICC* = 0.79 | + |  |
|  |  |  | VPA *ICC* = 0.86 | + |  |
|  |  |  | Leisure physical activity *ICC* = 0.80 | + |  |
|  |  |  | Household physical activity *ICC* = 0.73 | + |  |
| Modified version Active Australia Survey 1(MV – AAS1)^36^ | 159 women; 54-59 years | Median: 13 days | No physical activity category % agreement = 61% | ? | ? |
|  |  |  | Low PA category % agreement = 56% |  |  |
|  |  |  | MPA category % agreement = 40% |  |  |
|  |  |  | High physical activity category % agreement = 65% |  |  |
|  |  |  | Meet guidelines category % agreement = 76% |  |  |
|  |  |  | Frequency walking *rho* = 0.58 | - | - |
|  |  |  | Frequency MPA *rho* = 0.56 | - |  |
|  |  |  | Frequency VPA *rho* = 0.60 | - |  |
|  |  |  | Frequency Total physical activity *rho* = 0.58 | - |  |
|  |  |  | Mins/week walking *rho* = 0.58 | - |  |
|  |  |  | Mins/week MPA *rho* = 0.56 | - |  |
|  |  |  | Mins/week VPA *rho* = 0.61 | - |  |
|  |  |  | Mins/week Total physical activity *rho* = 0.64 | - |  |
| Modified version Active Australia Survey (MV – AAS2)^37^ | 63; 63% women; 49.5 ± 12.5 years | N.R. | Mins/week MPA *rho* = 0.40 (95% CI: 0.16 to 0.59) | - | - |
|  |  |  | Mins/week VPA *rho* = 0.64 (95% CI: 0.47 to 0.77) | - |  |
|  |  |  | Mins/week MVPA *rho* = 0.80 (95% CI: 0.68 to 0.87) | + |  |
|  |  |  | Mins/week walking *rho* = 0.76 (95% CI: 0.63 to 0.85) | - |  |
|  |  |  | Days/week MPA *kappa* = 0.63 (95% CI: 0.36 to 0.76) | - |  |
|  |  |  | Days/week VPA *kappa* = 0.43 (95% CI: 0.34 to 0.73) | - |  |
|  |  |  | Days/week MVPA *kappa* = 0.83 (95% CI: 0.61 to 0.93) | + |  |
|  |  |  | Days/week walking *kappa* = 0.64 (95% CI: 0.45 to 0.81) | - |  |
|  |  |  | Measurement error: Bland-Altman: LoA = -8.46 ± 110.96 + 0.54 x average of AAS administrations. | ? | ? |
| Adapted from Active Australia Survey (Adapt AAS)^38^ | 118; 53% women; 38 ± 15 years | 3 days | Mins/week men MPA *ICC* = 0.45 (95% CI: 0.21 to 0.64) | - | - |
|  |  |  | Mins/week men VPA *ICC* = 0.85 (95% CI: 0.74 to 0.91) | + |  |
|  |  |  | Mins/week men walk *ICC* = 0.22 (95% CI: -0.05 to 0.46) | - |  |
|  |  |  | Mins/week men Total physical activity *ICC* = 0.65 (95% CI: 0.33 to 0.84) | - |  |
|  |  |  | Days/week men MPA *ICC* = 0.71 (95% CI: 0.54 to 0.82) | + |  |
|  |  |  | Days/week men VPA *ICC* = 0.38 (95% CI: 0.13 to 0.59) | - |  |
|  |  |  | Days/week men walking *ICC* = 0.67 (95% CI: 0.48 to 0.78) | - |  |
|  |  |  | Mins/week women MPA *ICC* = 0.52 (95% CI: 0.32 to 0.68) | - |  |
|  |  |  | Mins/week women VPA *ICC* = 0.65 (95% CI: 0.48 to 0.77) | - |  |
|  |  |  | Mins/week women walk *ICC* = 0.75 (95% CI: 0.62 to 0.84) | + |  |
|  |  |  | Mins/week women Total physical activity *ICC* = 0.80 (95% CI: 0.69 to 0.87) | + |  |
|  |  |  | Days/week women MPA *ICC* = 0.53 (95% CI: 0.33 to 0.68) | - |  |
|  |  |  | Days/week women VPA *ICC* = 0.89 (95% CI: 0.82 to 0.93) | + |  |
|  |  |  | Days/week women walking *ICC* = 0.86 (95% CI: 0.77 to 0.91) | + |  |
|  |  |  | Meet guidelines men *kappa* = 0.64*** (agreement = 94.1%) | - |  |
|  |  |  | Meet guidelines women *kappa* = 0.55*** (agreement = 95.2%) | - |  |
| International Physical Activity Questionnaire – Walking Section (IPAQ-WS)^39^ | 1766; 55% women; 23-57 years | 3 days | IPAQ S7S *rho* = 0.77 | - | - |
|  |  |  | IPAQ SUS *rho* = 0.91 | + |  |
| Short Questionnaire  to Assess Health-enhancing physical activity (SQUASH)^40^ | 50; 28% women; 44 ± 6 years | 2 weeks | Total activity score *rho* = 0.58 | - | - |
|  |  |  | Within the intensity categories (2 to <4 MET) *rho* = 0.58 | - |  |
|  |  |  | Within the intensity categories (4 to <6.5 MET) *rho* = 0.54 | - |  |
|  |  |  | Within the intensity categories (>+ 6.5 MET) *rho* = 0.92 | + |  |
| European Prospective Investigation into Cancer and Nutrition Physical Activity Questionnaire (EPIC PAQ)^41^ | 182; 45% women; 50-65 years | 10 months | Total non-occup physical activity *rho* = 0.65*** (95% CI: 0.55 to 0.72) | - | - |
|  |  |  | VPA (self-rated) *rho* = 0.63*** (95% CI: 0.54 to 0.72) | - |  |
|  |  |  | VPA (MET-assigned) *rho* = 0.71*** (95% CI: 0.63 to 0.78) | - |  |
|  |  |  | LPA to MPA *rho* = 0.67*** (95% CI: 0.58 to 0.74) | - |  |
|  |  |  | Household physical activity *rho* = 0.73*** (95% CI: 0.66 to 0.79) | - |  |
|  |  |  | Recreational physical activity *rho* = 0.58*** (95% CI: 0.48 to 0.67) | - |  |
|  |  |  | Total physical activity index weighted *kappa* = 0.62*** (95% CI: 0.53 to 0.71) | - |  |
|  |  |  | Cambridge physical activity index weighted *kappa* = 0.66*** (95% CI: 0.58 to 0.74) | - |  |
|  |  |  | Measurement error: Although the mean difference in  MET-hours/week of non-occupational activity was small,  a Bland-Altman plot of these data (not presented) showed wide 95% LoA (-116.2 to 115.0). | ? | ? |
| 13-Item Physical Activity Questionnaire (13I-PAQ)^42^ | 54; N.R.; N.R. | 2 to 3 weeks | **Women** |  | + |
|  |  |  | Total physical activity *ICC* = 0.74 | + |  |
|  |  |  | Sport *ICC* = 0.83 | + |  |
|  |  |  | LTPA *ICC* = 0.81 | + |  |
|  |  |  | OHPA *ICC* = 0.63 | - |  |
|  |  |  | **Men** |  |  |
|  |  |  | Total physical activity *ICC* = 0.78 | + |  |
|  |  |  | Sport *ICC* = 0.92 | + |  |
|  |  |  | LTPA *ICC* = 0.79 | + |  |
|  |  |  | OHPA *ICC* = 0.59 | - |  |
| Questionnaire d’Activité Physique pour les Personnes Âgées (QAPPA)^43^ | 225; 62.7% women; 69.5 ± 7.3 years | 1 year | Moderate (METM) = *ICC* = 0.46 | - | - |
|  |  |  | Vigorous (METV) *ICC* = 0.63 | - |  |
|  |  |  | Moderate-to-vigorous (METT) *ICC* = 0.64 | - |  |
|  |  |  | "‘physically active vs inactive’ = 79.6% of individuals received the same classification at both test and retest (*kappa* = 0.44) | - |  |
|  |  |  | Measurement error: Bland Altman: There was no sizeable trend, but some variability was observed in both directions. | ? | ? |
| Incidental and Planned Exercise Questionnaire (IPEQ)^44^ | "Past Week Version" = 30 | 1 week | "Past Week Version" *ICC* = 0.77 | + | + |
|  | "Past 3 months Version" = 50 |  | "Past 3 months Version" *ICC* = 0.84 | + |  |
| Physical Activity Questionnaire for Elderly Japanese (PAQ-EJ)^45^ | 147; 58.50% women; 65-85 years | 1 month | Total Score *r* = 0.70 | - | - |
|  |  |  | Subtotal of lower intensity activity categories *r* = 0.64 | - |  |
|  |  |  | Subtotal of higher intensity activity categories *r* = 0.71 | - |  |
| The Longitudinal Ageing Study Amsterdam Physical Activity Questionnaire (LAPAQ)^46^ | 86; 52% women; 65.4–87.6 years | 2 weeks | Total physical activity *r* = 0.68 (95% CI: 0.55 to 0.80) | - | - |
|  |  |  | >= 6 METs *r* = 0.75 (95% CI: 0.47 to 0.87) | - |  |
|  |  |  | 3-5.99 METs *r* = 0.79 (95% CI: 0.69 to 0.88) | - |  |
|  |  |  | 2-2.99 METs *r* = 0.58 (95% CI: 0.42 to -0.72) | - |  |
|  |  |  | Total: ̄mean difference = 436, LOA = 436 ± 1.96*1260 (min/2 weeks) |  |  |
|  |  |  | Mild: mean difference = 309, LOA = 309 ± 1.96*1004 (min/2 weeks) |  |  |
|  |  |  | Moderate: mean difference = 102, LOA = 102 ± 1.96*436 (min/2 weeks) |  |  |
|  |  |  | Vigorous: mean difference = 23, LOA = 23 ± 1.96*258 (min/2 weeks) |  |  |
| Nordic Physical Activity Questionnaire (NPAQ-short)^47^ | 105; N.R.; N.R. | 2 weeks | MVPA open questions *rho* = 0.82*** | + | - |
|  |  |  | VPA open questions *rho* = 0.80*** | + |  |
|  |  |  | MVPA close questions weighted *kappa* = 0.66 (95% CI: 0.55 to 0.75) | - |  |
|  |  |  | VPA close questions weighted *kappa* = 0.59 (95% CI: 0.47 to 0.69) | - |  |
| Self-report physical activity questionnaire (SPAQ)^48^ | 150; 73.3% women; 60- over 80 years | 1 week | MET hours/week: |  | + |
|  |  |  | Total physical activity *r* = 0.93* (95% CI: 4.99 to 11.22) | + |  |
|  |  |  | LPA *r* = 0.91** (95% CI: -1.35 to 6.63) | + |  |
|  |  |  | MPA *r* = 0.94** (95% CI: -7.59 to -3.34) | + |  |
|  |  |  | Household physical activity *r* = 0.90** (95% CI: -6.07 to -2.76) | + |  |
|  |  |  | Occupational physical activity *r* = 0.69** (95% CI: -4.88 to -2.02) | - |  |
|  |  |  | Leisure time recreation *r* = 0.87** (95% CI: 6.4 to 11.79) | + |  |
|  |  |  | Leisure time exercise *r* = 0.96** (95% CI: -1.08 to 1.19) | + |  |
|  |  |  | Transportation physical activity *r* = 0.91* (95% CI: -2.94 to 4.62) | + |  |
| Transport and Physical Activity Questionnaire (TPAQ)^49^ | 166; 53.3% female, <30-≥65 years | 12.4 ± 6.6 days | For time spent in transport-related and recreational physical activity: |  | - |
|  |  |  | Walking for transport *ICC* = 0.59 (95% CI: 0.48 to 0.68) | - |  |
|  |  |  | Cycling for transport *ICC* = 0.61 (95% CI: 0.50 to 0.70) | - |  |
|  |  |  | Walking for recreation *ICC* = 0.48 (95% CI: 0.35 to 0.59) | - |  |
|  |  |  | Cycling for recreation *ICC* = 0.35 (95% CI: 0.20 to 0.47) | - |  |
|  |  |  | Moderate Leisure time physical activity *ICC* = 0.47 (95% CI: 0.34 to 0.58) | - |  |
|  |  |  | Vigorous Leisure time physical activity *ICC* = 0.63 (95% CI: 0.53 to 0.71) | - |  |
|  |  |  | Total physical activity *ICC* = 0.56 (95% CI: 0.45 to 0.66) | - |  |
|  |  |  | For frequency of participation in recreational physical activity: |  |  |
|  |  |  | Walking for recreation *ICC* = 0.80 (95% CI: 0.73 to 0.85) | + |  |
|  |  |  | Cycling for recreation *ICC* = 0.63 (95% CI: 0.53 to 0.71) | - |  |
|  |  |  | Moderate intensity Leisure time physical activity *ICC* = 0.13 (95% CI: 20.02 to 0.28) | - |  |
|  |  |  | Vigorous intensity Leisure time physical activity *ICC* = 0.52 (95% CI: 0.40 to 0.62) | - |  |
|  |  |  | Meet physical activity Guideline’s *kappa* = 0.60 | - |  |
| General Practice Physical Activity Questionnaire (GPPAQ)^50^ | 126; N.R.; N.R. | 3 months | Agreement = 56% |  | - |
|  |  |  | Weighted *Kappa* = 0.57 | - |  |
|  |  | 12 months | Agreement = 67% |  |  |
|  |  |  | Weighted *Kappa* = 0.63 | - |  |
| **Sedentary Behaviour** | | | | | |
| International Physical Activity Questionnaire - Sedentary Behavior (IPAQ-SB)^51^ | 257; 58% women; N.R. | 3 to 7 days | Short Form: |  | - |
|  |  |  | Weekday *rho* = 0.79 | - |  |
|  |  |  | Weekend *rho* = 0.84 | + |  |
|  |  |  | Total *rho* = 0.85 | + |  |
|  | 255; 56% women; N.R. |  | Long Form: |  | + |
|  |  |  | Weekday *rho* = 0.81 | + |  |
|  |  |  | Weekend *rho* = 0.84 | + |  |
|  |  |  | Total *rho* = 0.82 | + |  |
| Australian Longitudinal Study on Women’s Health - Sedentary Behavior Questions (ALSWH - SB Q)^52^ | 157 women; N.R. | 7–28 days | Weekday: |  | - |
|  |  |  | Transport *r* = 0.43; | - |  |
|  |  |  | Occup *ICC* = 0.79 (95% CI: 0.73 - 0.84) | + |  |
|  |  |  | TV *r* = 0.79 | - |  |
|  |  |  | Computer *ICC* = 0.63 (95% CI: 0.52 - 0.71) | - |  |
|  |  |  | Other leisure *r* = 0.34 | - |  |
|  |  |  | Weekend day: |  |  |
|  |  |  | Transport *r* = 0.31 | - |  |
|  |  |  | Occup *r* = 0.53 | - |  |
|  |  |  | TV *r* = 0.57 | - |  |
|  |  |  | Computer *ICC* = 0.72 (95% CI: 0.64 to 0.79) | + |  |
|  |  |  | Other leisure *r* = 0.31 | - |  |
|  | 96 men; N.R. | 7–28 days | Weekday: |  |  |
|  |  |  | Transport *r* = 0.60 | - |  |
|  |  |  | Occup *ICC* = 0.86 (95% CI: 0.79 to 0.90) | + |  |
|  |  |  | TV *ICC* = 0.65 (95% CI: 0.52 to 0.75) | - |  |
|  |  |  | Computer *ICC* = 0.62 (95% CI: 0.48 to 0.73) | - |  |
|  |  |  | Other leisure *r* = 0.38 | - |  |
|  |  |  | Weekend day: |  |  |
|  |  |  | Transport *r* = 0.40 | - |  |
|  |  |  | Occup *r* = 0.23 | - |  |
|  |  |  | TV *ICC* = 0.62 (95% CI: 0.48 to 0.73) | - |  |
|  |  |  | Computer *ICC* = 0.59 (95% CI: 0.44 to 0.71) | - |  |
|  |  |  | Other leisure *r* = 0.32 | - |  |
|  |  |  | Measurement error: Bland–Altman: Women self-report work-related sitting items on a weekday mean difference = -3.9 mins/day (LoA = -235.4 to 227.5) | ? | ? |
|  |  |  | Measurement error: Bland–Altman: Women self-report work-related sitting items on a weekend day mean difference = -5.6 mins/day (LoA = -125.1 to 113.9) | ? |  |
|  |  |  | Measurement error: Bland–Altman: Men self-report work-related sitting items on a weekday mean difference = -4.3 mins/day (LoA = -189.2 to 180.7) | ? |  |
|  |  |  | Measurement error: Bland–Altman: Men self-report work-related sitting items on a weekend day mean difference = -8.1 mins/day (LoA = -195.0 to 178.8) | ? |  |
| Self-reported sitting and breaks from sitting in the workplace (SBSW) ^53^ | 59; 54% women; 32.1± 9.9 years | 7 days | Sitting *ICC* = 0.78** (95% CI: 0.65 to 0.86) | + | - |
|  |  |  | Breaks from sitting *ICC* = 0.65** (95% CI: 0.48 to 0.78) | - |  |
| Workplace Sitting Breaks Questionnaire (SITBRQ)^54^ | 96; 62.5% women; ~18<60 years | 7-14 days | Frequency of breaks *rho* = 0.71 (95% CI: 0.59 to 0.79) | - | - |
|  |  |  | Frequency of breaks Cohen’s *kappa* = 0.74 (95% CI: 0.64 to 0.84) | + |  |
|  |  |  | Total duration of breaks *rho* = 0.59 (0.45 to 0.71) | - |  |
|  |  |  | Total duration of breaks Cohen’s *kappa* = 0.61 (0.38 to 0.85) | - |  |
|  |  |  | Total duration of breaks % correctly classified = 46.9% (36.9 to 56.9) |  | |
|  |  |  | Total duration of breaks % in same or adjacent category = 79.2% (71.0 to 87.3) |  |  |
|  |  |  | Frequency of breaks % correctly classified = 51.0% (95% CI: 41.0 to 61.0) |  |  |
|  |  |  | Frequency of breaks % in same or adjacent category = 85.4% (95% CI: 78.4 to 92.5) |  |  |
| Sedentary Behavior Questionnaire (SBQ)^55^ | 49; 67% women; 20.4 ± 1.3 years | 2 weeks | Weekday |  | + |
|  |  |  | TV *ICC* = 0.86 (95% CI: 0.76 to 0.92) | + |  |
|  |  |  | Computer games *ICC* = 0.83 (95% CI: 0.71 to .90) | + |  |
|  |  |  | Sit listen to music *ICC* = 0.71 (95% CI: 0.50 to 0.82) | + |  |
|  |  |  | Sit talk on telephone *ICC* = 0.81 (95% CI: 0.68 to 0.89) | + |  |
|  |  |  | Office/paperwork *ICC* = 0.77 (95% CI: 0.63 to 0.86) | + |  |
|  |  |  | Reading *ICC* = 0.64 (95% CI: 0.44 to 0.78) | - |  |
|  |  |  | Playing musical instrument *ICC* = 0.90 (95% CI: 0.82 to 0.94) | + |  |
|  |  |  | Arts and crafts *ICC* = 0.70 (95% CI: 0.53 to 0.82) | + |  |
|  |  |  | Sitting driving in car *ICC* = 0.76 (95% CI: 0.61 to 0.86) | + |  |
|  |  |  | Total scale *ICC* = 0.85 (95% CI: 0.75 to 0.91) | + |  |
|  |  |  | Weekend |  |  |
|  |  |  | TV *ICC* = 0.83 (95% CI: 0.72 to 0.90) | + |  |
|  |  |  | Computer games *ICC* = 0.80 (95% CI: 0.67 to 0.88) | + |  |
|  |  |  | Sit listen to music *ICC* = 0.67 (95% CI: 0.49 to 0.80) | - |  |
|  |  |  | Sit talk on telephone *ICC* = 0.73 (95% CI: 0.57 to 0.84) | + |  |
|  |  |  | Office/paperwork *ICC* = 0.64 (95% CI: 0.44 to 0.61) | - |  |
|  |  |  | Reading *ICC* = 0.48 (95% CI: 0.24 to 0.67) | - |  |
|  |  |  | Playing musical instrument *ICC* = 0.93 (95% CI: 0.87 to 0.96) | + |  |
|  |  |  | Arts and crafts *ICC* = 0.51 (95% CI: 0.27 to 0.69) | - |  |
|  |  |  | Sitting driving in car *ICC* = 0.72 (95% CI: 0.56 to 0.83) | + |  |
|  |  |  | Total scale *ICC* = 0.77 (95% CI: 0.63 to 0.86) | + |  |
| SED-GIH^56^ | 95; 69,5% women; 70.3 ± 5.0 years | Mean = 5.2 days | SED-GIH question *ICC* = 0.86 (CI = 0.79 to 0.90) | + | + |
|  |  |  | Agreement between the two SED-GIH question  answering occasions weighted *Kappa* = 0.77 (CI = 0.68 to 0.86) | + |  |
| Workforce Sitting Questionnaire (WSQ)^57^ | 91; N.R.; N.R. | 1 week | Workday |  | - |
|  |  |  | Transport *ICC* = 0.67 (95% CI: 0.54 to 0.77) | - |  |
|  |  |  | At work *ICC* = 0.63 (95% CI: 0.49 to 0.74) | - |  |
|  |  |  | Watching TV *ICC* = 0.91 (95% CI: 0.87 to 0.94) | + |  |
|  |  |  | Computer at home *ICC* = 0.56 (95% CI: 0.40 to 0.69) | - |  |
|  |  |  | Other leisure activities *ICC* = 0.68 (95% CI: 0.55 to 0.78) | - |  |
|  |  |  | Total, all domains *ICC* = 0.65 (95% CI: 0.51 to 0.75) | - |  |
|  |  |  | Non-Workday: |  |  |
|  |  |  | Transport *ICC* = 0.60 (95% CI: 0.45 to 0.72) | - |  |
|  |  |  | At work *ICC* = 0.50 (95% CI: 0.33 to 0.64) | - |  |
|  |  |  | Watching TV *ICC* = 0.79 (95% CI: 0.69 to 0.85) | + |  |
|  |  |  | Computer at home *ICC* = 0.81 (95% CI: 0.73 to 0.87) | + |  |
|  |  |  | Other leisure activities *ICC* = 0.59 (95% CI: 0.44 to 0.71) | - |  |
|  |  |  | Total, all domains *ICC* = 0.80 (95% CI: 0.72 to 0.87) | + |  |
|  |  |  | Average total, work and non-workdays *ICC* = 0.76 (95% CI: 0.66 to 0.83) | + |  |
| Japanese-Language Self-reported Measures for Assessing Adults Domain-Specific Sedentary Time (JSRM - SB)^58^ | 34; 41.2% women; 40.3 ± 11.4 years | 2 weeks | Workday |  | - |
|  |  |  | Car *ICC* = 0.85** (95% CI: 0.71 to 0.92) | + |  |
|  |  |  | Public transport *ICC* = 0.60** (95% CI: 0.33 to 0.78) | - |  |
|  |  |  | Work *ICC* = 0.89** (95% CI: 0.80 to 0.95) | + |  |
|  |  |  | TV *ICC* = 0.76** (95% CI: 0.58 to 0.88) | + |  |
|  |  |  | Computer use *ICC* = 0.72** (95% CI: 0.51 to 0.85) | + |  |
|  |  |  | Leisure *ICC* = 0.45** (95% CI: 0.15 to 0.68) | - |  |
|  |  |  | Total *ICC* = 0.77** (95% CI: 0.60 to 0.88) | + |  |
|  |  |  | Non-Workday |  |  |
|  |  |  | Car *ICC* = 0.53** (95% CI: 0.24 to 0.74) | - |  |
|  |  |  | Public transport *ICC* = 0.20 (95% CI: -0.15 to 0.50) | - |  |
|  |  |  | Work *ICC* = -0.07 (95% CI: -0.40 to 0.28) | - |  |
|  |  |  | TV *ICC* = 0.79** (95% CI: 0.63 to 0.89) | + |  |
|  |  |  | Computer use *ICC* = 0.72** (95% CI: 0.51 to 0.85) | + |  |
|  |  |  | Leisure *ICC* = 0.46** (95% CI: 0.14 to 0.69) | - |  |
|  |  |  | Total *ICC* = 0.53** (95% CI: 0.24 to 0.73) | - |  |
|  |  |  | Whole week |  |  |
|  |  |  | Car *ICC* = 0.83** (95% CI: 0.69 to 0.91) | + |  |
|  |  |  | Public transport *ICC* = 0.47** (95% CI: 0.17 to 0.70) | - |  |
|  |  |  | Work *ICC* = 0.83** (95% CI: 0.69 to 0.91) | + |  |
|  |  |  | TV *ICC* = 0.82** (95% CI: 0.67 to 0.91) | + |  |
|  |  |  | Computer use *ICC* = 0.74* (95% CI: 0.54 to 0.86) | + |  |
|  |  |  | Leisure *ICC* = 0.53** (95% CI: 0.25 to 0.73) | - |  |
|  |  |  | Total *ICC* = 0.74** (95% CI: 0.55 to 0.86) | + |  |
| Longitudinal Aging Study Amsterdam questionnaire (LASA) ^59^ | 63; N.R. but not different from validity (p>0.27) | 23 ± 8 days | Total ST (6 items) *ICC* = 0.71 (95% CI 0.57 to 0.81). | + | + |
| SB question of the Yale Physical Activity Survey (YPAS - SB)^60^ | 58; S79% women; 66 – 88 years | 10 days | Sitting *ICC*= 0.59** | - | - |
| Community Health Activities Model Program for Seniors SB questions (CHAMPS - SB) ^60^ | 58; 79% women; 66 – 88 yr | 10 days | Sitting *ICC*= 0.64** | - | - |
| Cancer Prevention Study-3 Sedentary Time Survey (CPS-3 sitting time)^61^ | 713; % women; 31-72 years | 1 yr | Total Sitting *rho* = 0.67 (95% CI: 0.63 to 0.71) | - | - |
|  |  |  | TV *rho* = 0.74 (95% CI: 0.70 to 0.77) | - |  |
|  |  |  | Other sitting *rho* = 0.71 (95% CI: 0.67 to 0.74) | - |  |
| **Sleep** | | | | | |
| BRFSS sleep questions (BRFSS Sleep)^62^ | 300; 68% women; 18-96 years | 14 days | Comparing first measure to 14 days, Cronbach α was 0.76 (F = 0.31, df = 114, p = 0.57). | ? | ? |
|  |  | 30 days | Comparing first measure to 30 days, Cronbach α was 0.85 (F = 4.96, df = 135, p = 0.03) | ? |  |
| **Physical Activity + Sedentary Behaviour** | | | | | |
| Kaiser Physical Activity Survey (KPAS)^63^ | 50 women; 20-60 years | 1 month | Caregiving score *ICC* = 0.01 | - | + |
|  |  |  | Housework score *ICC* = 0.79 | + |  |
|  |  |  | Housework/caregiving score *ICC* = 0.81 | + |  |
|  |  |  | Sports/exercise *ICC* = 0.84 | + |  |
|  |  |  | Active living habits *ICC* = 0.82 | + |  |
|  |  |  | Occupation *ICC* = 0.85 | + |  |
|  |  |  | 3-point summary *ICC* = 0.82 | + |  |
|  |  |  | 4-point summary *ICC* = 0.84 | + |  |
| Sedentary, Transportation and Activity Questionnaire (STAQ)^64^ | 32; 53.1 % women; 20-62 years | 1 month | SB: |  | - |
|  |  |  | Total ST *ICC* = 0.52 (95% CI: 0.22 to 0.73) | - |  |
|  |  |  | Work *ICC* = 0.71 (95% CI: 0.49 to 0.84) | + |  |
|  |  |  | Transport *ICC* = 0.28 (95% CI: −0.06 to 0.56) | - |  |
|  |  |  | Leisure Time *ICC* = 0.37 (95% CI: 0.03 to 0.62) | - |  |
|  |  |  | Leisure SB: |  |  |
|  |  |  | Total *ICC* = 0.64 (95% CI: 0.38 to 0.80) | - |  |
|  |  |  | Total screen time *ICC* = 0.70 (95% CI: 0.48 to 0.84) | + |  |
|  |  |  | Tv/DVD *ICC* = 0.79 (95% CI: 0.61 to 0.89) | + |  |
|  |  |  | Computer/tablet/video games *ICC* = 0.64 (0.38 to 0.80) | - |  |
|  |  |  | Reading, writing, listening to music, sewing *ICC* = 0.26 (95% CI: −0.08 to 0.55) | - |  |
|  |  |  | Transport-related activities: |  |  |
|  |  |  | Active transport: |  |  |
|  |  |  | Walking *ICC* =0.61 (95% CI: 0.35 to 0.79) | - |  |
|  |  |  | Walking + cycling *ICC* = 0.47 (95% CI: 0.16 to 0.70) | - |  |
|  |  |  | Passive transport: |  |  |
|  |  |  | All transportation type *ICC* = 0.50 (95% CI: 0.20 to 0.72) | - |  |
|  |  |  | Car *ICC* = 0.67 (95% CI: 0.42 to 0.82) | - |  |
| International Physical Activity Questionnaire (IPAQ)^65^ | Short form past week: 19-151; N.R.; 18-65 years | 8-10 days | TEE *rho* = 0.66 to 0.88 | + | + |
|  |  |  | ST *rho* = 0.71 to 0.95 | + |  |
|  |  |  | Guideline’s compliance % = 93 to 100 |  |  |
|  | Short form usual week: 28-257; N.R.; 18-65 years | 8-10 days | TEE *rho* = 0.65 to 0.84 | + | + |
|  |  |  | ST *rho* = 0.58 to 0.94 | + |  |
|  |  |  | Guideline’s compliance % = 77 to 99 |  |  |
|  | Long form past week: 19-149; N.R.; 18-65 years | 8-10 days | TEE *rho* = 0.70 to 0.91 | + | + |
|  |  |  | ST *rho* = 0.74 to 0.89 | + |  |
|  |  |  | Guideline’s compliance % = 92 to 100 |  |  |
|  | Long form usual week: 28-255; N.R.; 18-65 years | 8-10 days | TEE *rho* = 0.69 to 0.93 | + | + |
|  |  |  | ST *rho* = 0.74 to 0.93 | + |  |
|  |  |  | Guideline’s compliance % = 90 to 100 |  |  |
| Australian Women’s Activity Survey (AWAS)^66^ | 40 women; 33 ± 5 years | 1 week | Sitting Time *ICC* = 0.42 (0.13 to 0.64) | - | - |
|  |  |  | LPA *ICC* = 0.66 (0.43 to 0.81) | - |  |
|  |  |  | MPA = 0.74 (0.56 to 0.85) | + |  |
|  |  |  | Vigorous physical activity = 0.66 (0.43 to 0.80) | - |  |
|  |  |  | Total Activity = 0.73 (0.51 to 0.86) | + |  |
| Workers’ sitting- and walking-time questionnaire Time Method (WSWQ- t-method)^67^ | 64; 40% women; men mean age: 46.3 ± 8.0 years; women mean age: 35.8 ± 7.5 years | 1 week | Workday: |  | - |
|  |  |  | During working time: |  |  |
|  |  |  | Sitting *ICC* = 0.85 (95% CI: 0.76 to 0.91) | + |  |
|  |  |  | Walking/Standing *ICC* = 0.83 (95% CI: 0.73 to 0.89) | + |  |
|  |  |  | During non-working time |  |  |
|  |  |  | Sitting *ICC* = 0.49 (95% CI: 0.28 to 0.66) | - |  |
|  |  |  | Walking/Standing *ICC* = 0.56 (95% CI: 0.37 to 0.71) | - |  |
|  |  |  | Non-workday: |  |  |
|  |  |  | Sitting *ICC* = 0.64 (95% CI: 0.47 to 0.76) | - |  |
|  |  |  | Walking/Standing *ICC* = 0.48 (95% CI: 0.27 to 0.65) | - |  |
| The Physical Activity Scale for the Elderly (PASE)^68^ | 254; N.R.; N.R. | 3-7 week | PASE activity score *r* = 0.84 | + | + |
| Community Health Activities Model Program for Seniors physical activity self-report questionnaire + transport items (CHAMPS+transport)^69^ | 748; N.R.; N.R. | 6 months | Low-LPA *ICC* = 0.70 | + | - |
|  |  |  | High-LPA *ICC* = 0.68 | - |  |
|  |  |  | MVPA *ICC* = 0.66 | - |  |
|  |  |  | Total PA duration *ICC* = 0.69 | - |  |
|  |  |  | MVPA caloric expenditure *ICC* = 0.61 | - |  |
|  |  |  | Total physical activity caloric expenditure *ICC* = 0.64 | - |  |
|  |  |  | Sedentary time *ICC* = 0.56 | - |  |
| Community Healthy Activities Model Program for Seniors (CHAMPS)^70^ | 249; 63.9% women; 65 - 90 years | 6 months | Caloric expenditure per week in at  least moderate intensity physical  activities *ICC* = 0.67 | - | - |
|  |  |  | Frequency per week in at least  moderate intensity physical activities *ICC* = 0.58 (0.60) | - |  |
|  |  |  | Caloric expenditure per week in all  listed physical activities *ICC* = 0.66 | - |  |
|  |  |  | Frequency per week in all listed  physical activities *ICC* = 0.62 | - |  |
| Modified Version of the MONICA Optional Study on Physical Activity Questionnaire(Modified MOSPA-Q)^71^ | 75; N.R.; N.R. | 1 week | Walking *ICC* = 0.89 (95% CI = 0.84 to 0.93) | + | - |
|  |  |  | Lifting/Carrying *ICC* = 0.82 (95% CI = 0.73 to 0.88) | + |  |
|  |  |  | Standing *ICC* = 0.64 (95% CI = 0.48 to 0.75) | - |  |
|  |  |  | Sitting *ICC* = 0.54 (95% CI = 0.36 to 0.68) | - |  |
| Occupational Sitting and Physical Activity Questionnaire (OSPAQ)^71,72^ | 84; N.R.; N.R. | 1 week | Walking *ICC* = 0.73 (95% CI = 0.62 to 0.82) | + | + |
|  |  |  | Heavy Work *ICC* = 0.97 (95% CI = 0.96 to 0.98) | + |  |
|  |  |  | Standing *ICC* = 0.90 (95% CI = 0.85 to 0.93) | + |  |
|  |  |  | Sitting *ICC* = 0.89 (95% CI = 0.83 to 0.92) | + |  |
|  | 75; 82.7% women; 42.87 ± 11.34 years | 1 week | Walking *ICC* = 0.01 (95% CI = −0.21 to 0.23) | - | - |
|  |  |  | Standing *ICC* = 0.37 (95% CI = 0.17 to 0.54) | - |  |
|  |  |  | Sitting *ICC* = 0.44 (95% CI = 0.24 to 0.60) | - |  |
| Rapid Assessment Disuse Index (RADI)^73^ | 157; N.R.; N.R. | 12-16 days | Total physical activity: |  | - |
|  |  |  | Week *ICC* = 0.73 (95% CI = 0.64 to 0.79) | + |  |
|  |  |  | Month *ICC* = 0.67 (95% CI = 0.57 to 0.75) | - |  |
|  |  |  | Year *ICC* = 0.67 (95% CI = 0.57 to 0.74) | - |  |
|  |  |  | Stairs: |  |  |
|  |  |  | Week *ICC* = 0.74 (95% CI = 0.65 to 0.80) | + |  |
|  |  |  | Month *ICC* = 0.64 (95% CI = 0.54 to 0.73) | - |  |
|  |  |  | Year *ICC* = 0.59 (95% CI = 0.48 to 0.68) | - |  |
|  |  |  | Sitting Time: |  |  |
|  |  |  | Week *ICC* = 0.56 (95% CI = 0.44 to 0.67) | - |  |
|  |  |  | Month *ICC* = 0.58 (95% CI = 0.46 to 0.67) | - |  |
|  |  |  | Year *ICC* = 0.60 (95% CI = 0.49 to 0.69) | - |  |
|  |  |  | RADI scores: |  |  |
|  |  |  | Wk *ICC* = 0.76 (95% CI = 0.70 to 0.83) | + |  |
|  |  |  | Month *ICC* = 0.74 (95% CI = 0.65 to 0.80) | + |  |
|  |  |  | Year *ICC* = 0.71 (95% CI = 0.62 to 0.78) | + |  |
|  |  |  | Cumulative RADI score *ICC* = 0.79 (0.73–0.85) | + |  |
| Global Physical Activity Questionnaire (GPAQ)^74^ | 148; N.R.; N.R. | 3 to 7 days | Work Domain (categorical) |  | + |
|  |  |  | Sedentary *kappa* (agreement) = 0.74 (87.8%) | + |  |
|  |  |  | Vigorous intensity *kappa* (agreement) = 0.88 (97.9%) | + |  |
|  |  |  | Moderate intensity *kappa* (agreement) = 0.87 (94.6%) | + |  |
|  |  |  | Transport: walking and cycling *kappa* (agreement) = 0.75 (91.2%) | + |  |
|  |  |  | Leisure Domain (categorical): |  |  |
|  |  |  | Sedentary *kappa* (agreement)= 0.82 (91.6%) | + |  |
|  |  |  | Vigorous intensity *kappa* (agreement)= 0.89 (95.9%) | + |  |
|  |  |  | Moderate intensity *kappa* (agreement)= 0.88 (94.6%) | + |  |
|  |  |  | Work Domain (continuous) |  |  |
|  |  |  | Vigorous intensity *rho* = 0.88** | + |  |
|  |  |  | Moderate intensity *rho* = 0.85** | + |  |
|  |  |  | Work total *rho* =0.83** | + |  |
|  |  |  | Transport total *rho* = 0.90** | + |  |
|  |  |  | Leisure domain |  |  |
|  |  |  | Vigorous intensity *rho* = 0.89** | + |  |
|  |  |  | Moderate intensity *rho* = 0.83** | + |  |
|  |  |  | Total *rho* = 0.88** | + |  |
| **Sedentary Behaviour + Sleep** | | | | | |
| SIT-Q^75^ | 64; N.R.; N.R. | 1 month | Meals *ICC* = 0.60 (95% CI: 0.42 to 0.74) | - | - |
|  |  |  | Transportation *ICC* = 0.59 (95% CI: 0.41 to 0.73) | - |  |
|  |  |  | Occup = 0.86 (95% CI: 0.78 to 0.91) | + |  |
|  |  |  | Child and elder care *ICC* = 0.59 (95% CI: 0.40 to 0.73) | - |  |
|  |  |  | TV *ICC* = 0.84 (0.75 - 0.90) | + |  |
|  |  |  | Computer use *ICC* = 0.31 (0.07 - 0.52) | - |  |
|  |  |  | Leisure Time *ICC* = 0.61 (0.43 - 0.74) | - |  |
|  |  |  | Total sitting time *ICC* = 0.65 (0.49 - 0.78) | - |  |
|  |  |  | Categorical question: How often did you “break  up” the time you spent sitting during their primary “job” |  |  |
|  |  |  | absolute agreement = 55% |  |  |
|  |  |  | weighted *kappa* = 0.49 | - |  |
|  |  |  | Categorical question: breaking up time spent sitting whilst watching television |  |  |
|  |  |  | agreement = 61% |  |  |
|  |  |  | wk = 0.50 | - |  |
|  |  |  | Categorical question: how often participants ate snack foods whilst watching television |  |  |
|  |  |  | agreement = 56 |  |  |
|  |  |  | wk = 0.48 | - |  |
|  |  |  | Measurement error: Bland Altman: total sitting time (h/day): 95% LoA: 3.62 to 4.6; Overall, participants tended to report shorter periods of sedentary behaviour in the second administration of the SIT-Q. | ? | ? |
| **Physical Activity + Sedentary Behaviour + Sleep** | | | | | |
| Physical Activity Questionnaire (PAQ)^76^ | 111 men; N.R. | 6 months | Crude Total physical activity *rho* = 0.65* | - | - |
|  |  |  | Total activity score *rho*=0.65* | - |  |
|  |  |  | work/occupation *rho*=0.68* | - |  |
|  |  |  | home/household *rho*=0.64* | - |  |
|  |  |  | activity leisure-time *rho*=0.68* | - |  |
|  |  |  | inactive leisure (TV/reading) *rho*=0.68* | - |  |
|  |  |  | sleep *rho*=0.76* | - |  |
| Athens Physical Activity Questionnaire (APAQ)^77^ | 60; 67% women; 21 ± 2 years | 7 to 14 days | Total EEXP *ICC* = 0.95 (95% CI: 0.92 to 0.97)* | + | + |
|  |  |  | Occupational EEXP *ICC* = 0.78 (95% CI: 0.65 to 0.86)* | + |  |
|  |  |  | Home EEXP *ICC* = 0.58 (95% CI: 0.38 to 0.73)* | - |  |
|  |  |  | Recreational EEXP *ICC* = 0.85 (95% CI: 0.76 to 0.91)* | + |  |
| Sedentary Time and Activity Reporting Questionnaire (STAR-Q)^78^ | 95; N.R.; N.R. | 3 months | TEE, kcal/day *ICC* = 0.84 (95% CI: 0.77 - 0.89) | + | - |
|  |  |  | AEE, kcal/day *ICC* = 0.73 (95% CI: 0.62 - 0.81) | + |  |
|  |  |  | Sleeping *ICC* = 0.79 (95% CI: 0.70 - 0.85) | + |  |
|  |  |  | Stair-climbing, flights/day *ICC* = 0.45 (95% CI: 0.28 - 0.60) | - |  |
|  |  |  | Active sitting *ICC* = 0.45 (95% CI: 0.28 - 0.60) | - |  |
|  |  |  | Overall Activity: |  |  |
|  |  |  | SB *ICC* = 0.53 (95% CI: 0.37 - 0.66) | - |  |
|  |  |  | Light intensity *ICC* = 0.60 (95% CI: 0.46 - 0.71) | - |  |
|  |  |  | Moderate intensity *ICC* = 0.45 (95% CI: 0.28 - 0.60) | - |  |
|  |  |  | Vigorous intensity *ICC* = 0.65 (95% CI: 0.52 - 0.75) | - |  |
|  |  |  | Exercise, sports, and  leisure activity |  |  |
|  |  |  | General *ICC* = 0.63 (95% CI: 0.49 - 0.74) | - |  |
|  |  |  | Light intensity *ICC* = 0.44 (95% CI: 0.26 - 0.59) | - |  |
|  |  |  | Mod intensity *ICC* = 0.42 (95% CI: 0.24 - 0.57) | - |  |
|  |  |  | Vig intensity *ICC* = 0.59 (95% CI: 0.44 - 0.71) | - |  |
|  |  |  | Occup activity: |  |  |
|  |  |  | General *ICC* = 0.76 (95% CI: 0.66 - 0.83) | + |  |
|  |  |  | Sitting *ICC* = 0.69 (95% CI: 0.57 - 0.78) | - |  |
|  |  |  | SB *ICC* = 0.71 (95% CI: 0.60 - 0.80) | + |  |
|  |  |  | Light intensity *ICC* = 0.65 (95% CI: 0.52 - 0.75) | - |  |
|  |  |  | Mod intensity *ICC* = 0.44 (95% CI: 0.26 - 0.59) | - |  |
|  |  |  | Light leisure activity: |  |  |
|  |  |  | General *ICC* = 0.65 (95% CI: 0.52 - 0.75) | - |  |
|  |  |  | TV *ICC* = 0.72 (95% CI: 0.61 - 0.80) | + |  |
|  |  |  | Computer *ICC* = 0.60 (95% CI: 0.46 - 0.71) | - |  |
|  |  |  | Reading *ICC* = 0.56 (95% CI: 0.41 - 0.68) | - |  |
| Question 8 of the Paffenbarger Physical Activity Questionnaire (Q 8 PPAQ)^79^ | 130; 51.2% women; 44.0 ± 15.8 years for men, 45.3 ± 16.9 for women | 1 week | Light intensity *ICC* = 0.49 (95% CI: 0.38 to 0.59) | - | - |
|  |  |  | Light intensity SEM = 2.0 |  |  |
|  |  |  | Moderate intensity *ICC* = 0.61 (95% CI: 0.52 to 0.68) | - |  |
|  |  |  | Moderate intensity SEM = 1.7 |  |  |
|  |  |  | Vig intensity *ICC* = 0.67 (95% CI: 0.59 to 0.72) | - |  |
|  |  |  | VPA SEM = 1.0 |  |  |
|  |  |  | SB *ICC* = 0.71 (95% CI: 0.61 to 0.74) | + |  |
|  |  |  | SB SEM = 1.9 |  |  |
| EPIC-Norfolk Physical Activity Questionnaire (EPAQ2)^80^ | 399; 53.13 % women; men age: 65.0 ± 8.2 years; women age: 63.8 ± 8.4 years | 3 months | Men: |  | - |
|  |  |  | TV *rho* = 0.75 | - |  |
|  |  |  | TV k = 0.71 | - |  |
|  |  |  | Activity at home *kappa* = 0.77 | + |  |
|  |  |  | Activity at home *rho* = 0.61 | - |  |
|  |  |  | Activity at work *rho* =0.57 | - |  |
|  |  |  | Activity at work *kappa* = 0.79 | + |  |
|  |  |  | Recreational activity *rho* = 0.69 | - |  |
|  |  |  | Recreational activity *kappa* = 0.54 | - |  |
|  |  |  | VPA *rho* = 0.75 | - |  |
|  |  |  | VPA *kappa* = 0.58 | - |  |
|  |  |  | Physical activity index *rho* = 0.74 | - |  |
|  |  |  | Physical activity index *kappa* =0.66 | - |  |
|  |  |  | Women: |  |  |
|  |  |  | TV *rho* = 0.78 | - |  |
|  |  |  | TV *kappa* = 0.74 | + |  |
|  |  |  | Activity at home *rho* = 0.74 | - |  |
|  |  |  | Activity at home *kappa* = 0.62 | - |  |
|  |  |  | Activity at work *rho* =0.37 | - |  |
|  |  |  | Activity at work *kappa* = 0.82 | + |  |
|  |  |  | Recreational activity *rho* = 0.64 | - |  |
|  |  |  | Recreational activity *kappa* = 0.55 | - |  |
|  |  |  | VPA *rho* = 0.41 | - |  |
|  |  |  | VPA *kappa* = 0.67 | - |  |
|  |  |  | Physical activity index *rho* = 0.72 | - |  |
|  |  |  | Physical activity index *kappa* =0.70 | + |  |
| Workers’ sitting- and walking-time questionnaire Percentage Method (WSWQ - p-method)^67^ | 64; 40% women; men age: 46.3 ± 8.0 years; women age: 35.8 ± 7.5 years | 1 week | Workday: |  | + |
|  |  |  | During working time: |  |  |
|  |  |  | Sitting *ICC* = 0.83 (95% CI: 0.73 to 0.89) | + |  |
|  |  |  | Walking/Standing *ICC* = 0.85 (95% CI: 0.76 to 0.90) | + |  |
|  |  |  | During non-working time |  |  |
|  |  |  | Sitting *ICC* = 0.71 (95% CI: 0.56 to 0.81) | + |  |
|  |  |  | Walking/Standing *ICC* = 0.77 (95% CI: 0.65 to 0.85) | + |  |
|  |  |  | Non-workday: |  |  |
|  |  |  | Sitting *ICC* = 0.78 (95% CI: 0.66 to 0.86) | + |  |
|  |  |  | Walking/Standing *ICC* = 0.79 (95% CI: 0.68–0.87) | + |  |
| New Questionnaire on Physical Activity (NQPA)^81^ | 112; 44.64% women; N.R. | 5 months / men | Rest *r* = 0.67 (95% CI: 0.50 to 0.79) | - | - |
|  |  |  | Occupational *r* = 0.90 (95% CI: 0.84 to 0.94) | + |  |
|  |  |  | Leisure Time *r* = 0.85 (95% CI: 0.76 to 0.91) | + |  |
|  |  | 5 months / women | Rest *r* = 0.65 (95% CI: 0.46 to 0.79) | - |  |
|  |  |  | Occupational *r* = 0.79 (95% CI: 0.65 to 0.87) | - |  |
|  |  |  | Leisure Time *r* = 0.68 (95% CI: 0.50 to 0.81) | - |  |
|  | 109; 47,70% women; N.R. | 11 months / men | Rest *r* = 0.71 (95% CI: 0.56 to 0.82) | - |  |
|  |  |  | Occupational *r* = 0.89 (95% CI: 0.83 to 0.94) | + |  |
|  |  |  | Leisure Time *r* = 0.72 (95% CI: 0.56 to 0.82) | - |  |
|  |  | 11 months / women | Rest *r* = 0.66 (95% CI: 0.47 to 0.79) | - |  |
|  |  |  | Occupational *r* = 0.80 (95% CI: 0.68 to 0.88) | + |  |
|  |  |  | Leisure Time *r* = 0.69 (95% CI: 0.52 to 0.81) | - |  |
|  |  |  | Measurement error: Bland-Altman: “The limits of 2 standard deviations below and above the mean difference were −3368 and 3243 kJ." | ? | ? |
| Web-Based Physical Activity Questionnaire (Active-Q)^82^ | 148 men; 33-86 years | 12 days | SB *ICC* = 0.80 (95% CI: 0.74 to 0.86) | + | - |
|  |  |  | LPA *ICC* = 0.66 (95% CI: 0.57 to 0.75) | + |  |
|  |  |  | SB+LPA *ICC* = 0.67 (95% CI: 0.58 to 0.76) | - |  |
|  |  |  | MPA *ICC* = 0.69 (95% CI: 0.60 to 0.77) | - |  |
|  |  |  | VPA *ICC* = 0.51 (95% CI: 0.39 to 0.63) | - |  |
|  |  |  | MVPA *ICC* = 0.67 (95% CI: 0.58 to 0.76) | - |  |
| Flemish Physical Activity Computerized Questionnaire (FPACQ) ^83^ | 66 employed/unemployed: 31 men: 39.23 ± 11.65 years; 35 women: 41.46 ± 12.62 years | 2 weeks | Employed/Unemployed Men |  | + |
|  | 36 retired: 20 men: 63.65 ± 6.05 years; 16 women: 63.31 ± 3.94 years |  | Time/week spent on sports participation *ICC* = 0.87 (95% CI: 0.75 to 0.94) | + |  |
|  |  |  | Energy expenditure/week on sports participation *ICC* = 0.94 (95% CI: 0.88 to 0.97) | + |  |
|  |  |  | Average energy expenditure on sports participation *ICC* = 0.97 (95% CI: 0.94 to 0.98) | + |  |
|  |  |  | Time/week spent eating *ICC* = 0.74 (95% CI: 0.53 to 0.86) | + |  |
|  |  |  | Time/week spent sleeping *ICC* = 0.84 (95% CI: 0.70 to 0.92) | + |  |
|  |  |  | Time/week spent watching television or videos or playing computer games *ICC* = 0.93 (95% CI: 0.86 to 0.97) | + |  |
|  |  |  | Time/week spent on leisure-time active transportation *ICC* = 0.75 (95% CI: 0.55 to 0.87) | + |  |
|  |  |  | Time/week spent on active leisure-time activities *ICC* = 0.79 (95% CI: 0.62 to 0.90) | + |  |
|  |  |  | Energy expenditure/week on active leisure time-activities *ICC* = 0.84 (95% CI: 0.70 to 0.92) | + |  |
|  |  |  | Average energy expenditure on active leisure-time activities *ICC* = 0.90 (95% CI: 0.81 to 0.95) | + |  |
|  |  |  | Time/week spent on occupation and transportation to and from occupation *ICC* = 0.97 (95% CI: 0.94 to 0.99) | + |  |
|  |  |  | Energy expenditure/week on occupation and transportation  to and from occupation *ICC* = 0.94 (95% CI: 0.88 to 0.97) | + |  |
|  |  |  | Average energy expenditure on occupation and transportation to and from occupation *ICC* = 0.95 (95% CI: 0.91 to 0.98) | + |  |
|  |  |  | Overall energy expenditure during a usual week *ICC* = 0.95 (95% CI: 0.89 to 0.97) | + |  |
|  |  |  | Physical activity level (MET) *ICC* = 0.92 (95% CI: 0.84 to 0.96) | + |  |
|  |  |  | Employed/Unemployed Women |  |  |
|  |  |  | Time/week spent on sports participation *ICC* = 0.91 (95% CI: 0.82 to 0.95) | + |  |
|  |  |  | Energy expenditure/week on sports participation *ICC* = 0.91 (95% CI: 0.83 to 0.95) | + |  |
|  |  |  | Average energy expenditure on sports participation *ICC* = 0.74 (95% CI: 0.55 to 0.86) | + |  |
|  |  |  | Time/week spent eating *ICC* = 0.67 (95% CI: 0.43 to 0.82) | - |  |
|  |  |  | Time/week spent sleeping *ICC* = 0.83 (95% CI: 0.70 to 0.91) | + |  |
|  |  |  | Time/week spent watching television or videos or playing computer games *ICC* = 0.92 (95% CI:.84 to 0.96) | + |  |
|  |  |  | Time/week spent on leisure-time active transportation *ICC* = 0.71 (95% CI: 0.50 to 0.84) | + |  |
|  |  |  | Time/week spent on active leisure-time activities *ICC* = 0.85 (95% CI: 0.73 to 0.92) | + |  |
|  |  |  | Energy expenditure/week on active leisure time-activities *ICC* = 0.86 (95% CI: 0.75 to 0.93) | + |  |
|  |  |  | Average energy expenditure on active leisure-time activities *ICC* = 0.94 (95% CI: 0.88 to 0.97) | + |  |
|  |  |  | Time/week spent on occupation and transportation to and from occupation *ICC* = 0.99 (95% CI: 0.98 to 0.99) | + |  |
|  |  |  | Energy expenditure/week on occupation and transportation  to and from occupation *ICC* = 0.97 (95% CI: 0.94 to 0.98) | + |  |
|  |  |  | Average energy expenditure on occupation and transportation to and from occupation *ICC* = 0.98 (95% CI: 0.96 to 0.99) | + |  |
|  |  |  | Overall energy expenditure during a usual week *ICC* = 0.92 (95% CI: 0.85 to 0.96) | + |  |
|  |  |  | Physical activity level (MET) *ICC* = 0.78 (95% CI: 0.61 to 0.88) | + |  |
|  |  |  | Retired Men |  |  |
|  |  |  | Time/week spent on sports participation *ICC* = 0.68 (95% CI: 0.37 to 0.86) | - |  |
|  |  |  | Energy expenditure/week on sports participation *ICC* = 0.68 (95% CI: 0.36 to 0.86) | - |  |
|  |  |  | Average energy expenditure on sports participation *ICC* = 0.60 (95% CI: 0.23 to 0.82) | - |  |
|  |  |  | Time/week spent eating *ICC* = 0.24 (95% CI: -0.20 to 0.61) | - |  |
|  |  |  | Time/week spent sleeping *ICC* = 0.94 (95% CI: 0.86 to 0.98) | + |  |
|  |  |  | Time/week spent watching television or videos or playing computer games *ICC* = 0.76 (95% CI: 0.49 to 0.89) | + |  |
|  |  |  | Time/week spent on leisure-time active transportation *ICC* = 0.81 (95% CI: 0.58 to 0.92) | + |  |
|  |  |  | Time/week spent on active leisure-time activities *ICC* = 0.83 (95% CI: 0.62 to 0.93) | + |  |
|  |  |  | Energy expenditure/week on active leisure time-activities *ICC* = 0.83 (95% CI: 0.63 to 0.93) | + |  |
|  |  |  | Average energy expenditure on active leisure-time activities *ICC* = 0.71 (95% CI: 0.41 to 0.87) | + |  |
|  |  |  | Overall energy expenditure during a usual week *ICC* = 0.90 (95% CI: 0.76 to 0.96) | + |  |
|  |  |  | Physical activity level (MET) *ICC* = 0.89 (95% CI: 0.76 to 0.96) | + |  |
|  |  |  | Retired Women |  |  |
|  |  |  | Time/week spent on sports participation *ICC* = 0.92 (95% CI: 0.79 to 0.97) | + |  |
|  |  |  | Energy expenditure/week on sports participation *ICC* = 0.91 (95% CI: 0.78 to 0.97) | + |  |
|  |  |  | Average energy expenditure on sports participation *ICC* = 0.94 (95% CI: 0.84 to 0.98) | + |  |
|  |  |  | Time/week spent eating *ICC* = 0.14 (95% CI: -0.35 to 0.58) | - |  |
|  |  |  | Time/week spent sleeping *ICC* = 0.90 (95% CI: 0.75 to 0.97) | + |  |
|  |  |  | Time/week spent watching television or videos or playing computer games *ICC* = 0.89 (95% CI: 0.72 to 0.96) | + |  |
|  |  |  | Time/week spent on leisure-time active transportation *ICC* = 0.57 (95% CI: 0.14 to 0.83) | - |  |
|  |  |  | Time/week spent on active leisure-time activities *ICC* = 0.64 (95% CI: 0.24 to 0.85) | - |  |
|  |  |  | Energy expenditure/week on active leisure time-activities *ICC* = 0.71 (95% CI: 0.35 to 0.89) | + |  |
|  |  |  | Average energy expenditure on active leisure-time activities *ICC* = 0.77 (95% CI: 0.48 to 0.91) | + |  |
|  |  |  | Overall energy expenditure during a usual week *ICC* = 0.96 (95% CI: 0.90 to 0.99) | + |  |
|  |  |  | Physical activity level (MET) *ICC* = 0.77 (95% CI: 0.47 to 0.91) | + |  |

Table 1. abbreviations: n= Sample Number; SD= Standard Deviation; N.R.= Not Reported; VPA= Vigorous Physical Activity; MPA= Moderate Physical Activity; MVPA= Moderate-Vigorous Physical Activity; MET= Metabolic Equivalent Task; ICC= Intraclass Correlations; LoA= Limits of Agreement; SEM= Standard Error of Measurement; * p≤0.05; ** p≤0.001; ***p≤0.0001; ? =Doubtful; + = Adequate; - = Inadequate

**References**

30. Kurtze N, Rangul V, Hustvedt B, Flanders WD. Reliability and validity of self-reported physical activity in the Nord-Trøndelag Health Study -- HUNT 1. Scandinavian Journal of Public Health. 2008;36(1):52-61. doi:10.1177/1403494807085373

31. Friedenreich CM, Courneya KS, Neilson HK, et al. Reliability and validity of the Past Year Total Physical Activity Questionnaire. American Journal of Epidemiology. 2006;163(10):959-970. doi:aje/kwj112

32. Meriwether RA, McMahon PM, Islam N, Steinmann WC. Physical Activity Assessment: Validation of a Clinical Assessment Tool. American Journal of Preventive Medicine. 2006;31(6):484-491. doi:10.1016/j.amepre.2006.08.021

33. Jacobs DR, Ainsworth BE, Hartman TJ, Leon AS. A simultaneous evaluation of 10 commonly used physical activity questionnaires. / Evaluation simultanee de 10 questionnaires couramment utilises sur les activites physiques. Medicine & Science in Sports & Exercise. 1993;25(1):81-91.

34. Milton K, Bull FC, Bauman A. Reliability and validity testing of a single-item physical activity measure. British Journal of Sports Medicine. 2011;45(3):203-208.

35. Chasean-Taber L, Erickson JB, Nasca PC, Chasan-Taber S, Freedson PS. Validity and reproducibility of a physical activity questionnaire in women. / Validite et reproductibilite d ' un questionnaire sur l ' activite physique chez des femmes. Medicine & Science in Sports & Exercise. 2002;34(6):987-992.

36. Brown WJ, Burton NW, Marshall AL, Miller YD. Reliability and validity of a modified self-administered version of the Active Australia physical activity survey in a sample of mid-age women. Australian & New Zealand Journal of Public Health. 2008;32(6):535-541.

37. Fjeldsoe BS, Winkler EAH, Marshall AL, Eakin EG, Reeves MM. Active adults recall their physical activity differently to less active adults: test-retest reliability and validity of a physical activity survey. Health Promotion Journal of Australia. 2013;24(1):26-31. doi:10.1071/HE12912

38. Timperio A, Salmon J, Crawford D. Validity and reliability of a physical activity recall instrument among overweight and non-overweight men and women. Journal of Science & Medicine in Sport. 2003;6(4):477-491.

39. van der Ploeg HP, Tudor-Locke C, Marshall AL, et al. Reliability and validity of the international physical activity questionnaire for assessing walking. Res Q Exerc Sport. Mar 2010;81(1):97-101. doi:10.1080/02701367.2010.10599632

40. Wendel-Vos GW, Schuit AJ, Saris WH, Kromhout D. Reproducibility and relative validity of the short questionnaire to assess health-enhancing physical activity. Journal of clinical epidemiology. 2003;56(12):1163-1169.

41. Cust AE, Smith BJ, Chau J, et al. Validity and repeatability of the EPIC Physical Activity Questionnaire: A validation study using accelerometers as an objective measure. The international journal of behavioral nutrition and physical activity. 2008;5

42. Nikolaidis PT, Säcklova M. Validity against health-related fitness and reliability of physical activity questionnaire in young female and male adults. Journal of Physical Education & Sport. 2011;11(3):342-348.

43. de Souto Barreto P. Construct and convergent validity and repeatability of the Questionnaire d’Activité Physique pour les Personnes Âgées (QAPPA), a physical activity questionnaire for the elderly. Public Health. 2013;127(9):844-853. doi:10.1016/j.puhe.2012.10.018

44. Delbaere K. Evaluation of the incidental and planned activity questionnaire for older people. British Journal of Sports Medicine. 2010;44(14):1029-1034.

45. Yasunaga A, Park H, Watanabe E, et al. Development and evaluation of the physical activity questionnaire for elderly Japanese: The Nakanojo study. Journal of Aging and Physical Activity. 2007;15(4):398-411. doi:10.1123/japa.15.4.398

46. Siebeling L, Wiebers S, Beem L, Puhan MA, Ter Riet G. Validity and reproducibility of a physical activity questionnaire for older adults: questionnaire versus accelerometer for assessing physical activity in older adults. Clinical epidemiology. 2012;4:171.

47. Danquah IH, Petersen CB, Skov SS, Tolstrup JS. Validation of the NPAQ-short - a brief questionnaire to monitor physical activity and compliance with the WHO recommendations. BMC Public Health. 2018;18(1):N.PAG-N.PAG. doi:10.1186/s12889-018-5538-y

48. Visuthipanich V, Sirapo-ngam Y, Malathum P, Kijboonchoo K, Vorapongsathorn T, Winters-Stone K. Physical activity questionnaire development and testing among elderly community-dwelling Thais. Thai Journal of Nursing Research. 2009;13(4):249-267.

49. Adams EJ, Goad M, Sahlqvist S, Bull FC, Cooper AR, Ogilvie D. Reliability and validity of the Transport and Physical Activity Questionnaire (TPAQ) for assessing physical activity behaviour. PLoS ONE. 2014;9(9)

50. Ahmad S, Harris T, Limb E, et al. Evaluation of reliability and validity of the General Practice Physical Activity Questionnaire (GPPAQ) in 60–74 year old primary care patients. BMC family practice. 2015;16(1):113.

51. Rosenberg DE, Bull FC, Marshall AL, Sallis JF, Bauman AE. Assessment of sedentary behavior with the International Physical Activity Questionnaire. Journal of Physical Activity & Health. 2008;5(Suppl1):S30-S44.

52. Marshall AL, Miller YD, Burton NW, Brown WJ. Measuring total and domain-specific sitting: a study of reliability and validity. Med Sci Sports Exerc. Jun 2010;42(6):1094-102. doi:10.1249/MSS.0b013e3181c5ec18

53. Sudholz B, Ridgers ND, Mussap A, Bennie J, Timperio A, Salmon J. Reliability and validity of self-reported sitting and breaks from sitting in the workplace. J Sci Med Sport. Jul 2018;21(7):697-701. doi:10.1016/j.jsams.2017.10.030

54. Pedisic Z, Bennie JA, Timperio AF, et al. Workplace Sitting Breaks Questionnaire (SITBRQ): an assessment of concurrent validity and test-retest reliability. BMC Public Health. Dec 5 2014;14:1249. doi:10.1186/1471-2458-14-1249

55. Rosenberg DE, Norman GJ, Wagner N, Patrick K, Calfas KJ, Sallis JF. Reliability and validity of the Sedentary Behavior Questionnaire (SBQ) for adults. Journal of Physical Activity & Health. 2010;7(6):697-705. doi:10.1123/jpah.7.6.697

56. Larsson K, Kallings LV, Ekblom Ö, Blom V, Andersson E, Ekblom MM. Criterion validity and test-retest reliability of SED-GIH, a single item question for assessment of daily sitting time. BMC public health. 2019;19(1):17.

57. Chau JY, Van Der Ploeg HP, Dunn S, Kurko J, Bauman AE. A tool for measuring workers' sitting time by domain: the Workforce Sitting Questionnaire. British journal of sports medicine. 2011;45(15):1216-1222.

58. Ishii K, Shibata A, Kurita S, et al. Validity and reliability of Japanese-language self-reported measures for assessing adults domain-specific sedentary time. Journal of epidemiology. 2017:JE20170002.

59. Visser M, Koster A. Development of a questionnaire to assess sedentary time in older persons–a comparative study using accelerometry. BMC geriatrics. 2013;13(1):80.

60. Gennuso KP, Matthews CE, Colbert LH. Reliability and validity of 2 self-report measures to assess sedentary behavior in older adults. Journal of Physical Activity & Health. 2015;12(5):727-732. doi:10.1123/jpah.2013-0546

61. Rees-Punia E, Matthews CE, Evans EM, et al. Demographic-specific validity of the cancer prevention study-3 sedentary time survey. Medicine and science in sports and exercise. 2019;51(1):41.

62. Jungquist CR, Mund J, Aquilina AT, et al. Validation of the Behavioral Risk Factor Surveillance System Sleep Questions. J Clin Sleep Med. Mar 2016;12(3):301-10. doi:10.5664/jcsm.5570

63. Ainsworth BE, Sternfeld B, Richardson MT, Jackson K. Evaluation of the Kaiser Physical Activity Survey in women. Medicine & Science in Sports & Exercise. 2000;32(7):1327-1334.

64. Mensah K, Maire A, Oppert J-M, et al. Assessment of sedentary behaviors and transport-related activities by questionnaire: a validation study. BMC Public Health. 2016;16(1):1-9. doi:10.1186/s12889-016-3412-3

65. Craig CL, Marshall AL, Sjöström M, et al. International physical activity questionnaire: 12-country reliability and validity. Med Sci Sports Exerc. Aug 2003;35(8):1381-95. doi:10.1249/01.mss.0000078924.61453.fb

66. Fjeldsoe BS, Marshall AL, Miller YD. Measurement properties of the Australian Women's Activity Survey. Medicine & Science in Sports & Exercise. 2009;41(5):1020-1033. doi:10.1249/MSS.0b013e31819461c2

67. Matsuo T, Sasai H, So R, Ohkawara K. Percentage-method improves properties of workers’ sitting-and walking-time questionnaire. Journal of epidemiology. 2016;26(8):405-412.

68. Washburn RA, Smith KW, Jette AM, Janney CA. The Physical Activity Scale for the Elderly (PASE): Development and evaluation. Journal of Clinical Epidemiology. 1993;46(2):153-162. doi:10.1016/0895-4356(93)90053-4

69. Hekler EB, Buman MP, Haskell WL, et al. Reliability and validity of CHAMPS self-reported sedentary-to-vigorous intensity physical activity in older adults. Journal of Physical Activity and Health. 2012;9(2):225-236.

70. Stewart AL, Mills KM, King AC, Haskell WL, Gillis D, Ritter PL. CHAMPS physical activity questionnaire for older adults: outcomes for interventions. / CHAMPS: Questionnaire sur les activites physiques des personnes agees: resultats pour de futures interventions. Medicine & Science in Sports & Exercise. 2001;33(7):1126-1141.

71. Chau JY, Van Der Ploeg HP, Dunn S, Kurko J, Bauman AE. Validity of the Occupational Sitting and Physical Activity Questionnaire. Medicine & Science in Sports & Exercise. 2012;44(1):118-125.

72. Pedersen SJ, Kitic CM, Bird M-L, Mainsbridge CP, Cooley PD. Is self-reporting workplace activity worthwhile? Validity and reliability of occupational sitting and physical activity questionnaire in desk-based workers. BMC Public Health. 2016;16(1):836-836. doi:10.1186/s12889-016-3537-4

73. Shuval K, Harold WK, III, Bernstein I, et al. Sedentary behaviour and physical inactivity assessment in primary care: the Rapid Assessment Disuse Index (RADI) study. British Journal of Sports Medicine. 2014;48(3):250-255.

74. Bull FC, Maslin TS, Armstrong T. Global Physical Activity Questionnaire (GPAQ): Nine country reliability and validity study. Journal of Physical Activity & Health. 2009;6(6):790-804.

75. Lynch BM, Friedenreich CM, Khandwala F, Liu A, Nicholas J, Csizmadi I. Development and testing of a past year measure of sedentary behavior: the SIT-Q. BMC Public Health. Sep 1 2014;14:899. doi:10.1186/1471-2458-14-899

76. Norman A, Bellocco R, Bergström A, Wolk A. Validity and reproducibility of self-reported total physical activity--differences by relative weight. Int J Obes Relat Metab Disord. May 2001;25(5):682-8. doi:10.1038/sj.ijo.0801597

77. Kavouras SA, Maraki MI, Kollia M, Gioxari A, Jansen LT, Sidossis LS. Development, reliability and validity of a physical activity questionnaire for estimating energy expenditure in Greek adults. Science & Sports. 2016;31(3):e47-e53.

78. Csizmadi I, Neilson HK, Kopciuk KA, et al. The Sedentary Time and Activity Reporting Questionnaire (STAR-Q): reliability and validity against doubly labeled water and 7-day activity diaries. American journal of epidemiology. 2014;180(4):424-435.

79. Simpson K, Parker B, Capizzi J, et al. Validity and Reliability of Question 8 of the Paffenbarger Physical Activity Questionnaire Among Healthy Adults. Journal of Physical Activity & Health. 2015;12(1):116-123.

80. Wareham NJ, Jakes RW, Rennie KL, Mitchell J, Hennings S, Day NE. Validity and repeatability of the EPIC-Norfolk physical activity questionnaire. International journal of epidemiology. 2002;31(1):168-174.

81. Pols MA, Peeters PH, Ocke MC, et al. Relative validity and repeatability of a new questionnaire on physical activity. Preventive Medicine. 1997;26(1):37-43.

82. Bonn SE, Bergman P, Lagerros YT, Sjölander A, Bälter K. A validation study of the web-based physical activity questionnaire active-Q against the GENEA accelerometer. JMIR research protocols. 2015;4(3):e86.

83. Matton L, Wijndaele K, Duvigneaud N, et al. Reliability and Validity of the Flemish Physical Activity Computerized Questionnaire in Adults. Research Quarterly for Exercise & Sport. 2007;78(4):293-306.
