# Supplementary material for: Questionnaires measuring movement behaviours in adults and older adults: Content description and measurement properties. A systematic review
Source: PLoS One. 2022 Mar 11;17(3):e0265100. doi: 10.1371/journal.pone.0265100 (PMC8916622; doi:10.1371/journal.pone.0265100)
Supplement: S4 Table — (DOCX) [file pone.0265100.s007.docx]

**Supporting table 4 – Responsiveness**

| **Questionnaire** |  | Sample | Responsiveness | | | Quality of validity results | Overall Quality |
| --- | --- | --- | --- | --- | --- | --- | --- |
|  |  | n; % Women; Age mean ± SD or Age range (years) | Type |  | Results |  |  |
| Community Healthy Activities Model Program for Seniors (CHAMPS)^70^ |  | 164; N.R. | Comparison between subgroups |  | Caloric expenditure per week in, at least, moderate intensity physical activities effect size = 0.38; F test = F_1,159_ = 8.84, *p* = 0.003. Within group change: Intervention 487 calories (t=3.65, *p* < 0.001), Control 5 calories (NS) | + | + |
|  |  |  |  |  | Frequency per week in, at least, moderate intensity physical activities effect size = 0.54; F test = F_1,159_ = 6.55, *p* = 0.01; Within group change: Intervention 3.1 calories (t=5.55, *p* < 0.001), Control 0.99 calories (NS) | + |  |
|  |  |  |  |  | Caloric expenditure per week in all listed physical activities effect size = 0.42; F test = F_1,159_ = 9.06, *p* = 0.003; Within group change: Intervention 687 calories  (t=3.67, *p* < 0.001), Control 210 calories (NS) | + |  |
|  |  |  |  |  | Frequency per week in all listed physical activities effect size = 0.64; F test = F_1,159_ = 16.39, *p* = 0.0001; Within group change: Intervention 5.18 calories  (t=6.80, *p* < 0.001), Control 0.58 calories (NS) | + |  |

Abbreviations: n= Sample Number; SD= Standard Deviation; NS= Not significant; N.R.= Not reported

**References**

70. Stewart AL, Mills KM, King AC, Haskell WL, Gillis D, Ritter PL. CHAMPS physical activity questionnaire for older adults: outcomes for interventions. / CHAMPS: Questionnaire sur les activites physiques des personnes agees: resultats pour de futures interventions. Medicine & Science in Sports & Exercise. 2001;33(7):1126-1141.
