# Supplementary material for: Questionnaires measuring movement behaviours in adults and older adults: Content description and measurement properties. A systematic review
Source: PLoS One. 2022 Mar 11;17(3):e0265100. doi: 10.1371/journal.pone.0265100 (PMC8916622; doi:10.1371/journal.pone.0265100)
Supplement: S5 Table — (DOCX) [file pone.0265100.s008.docx]

**Supporting table 5 – Risk of bias**

| **Questionnaire** | Statistical Methods | | Overall Rating* |
| --- | --- | --- | --- |
|  | 1. Continuous scores: Correlations, or the area under the receiver operating curve calculated? | 2.Dichotomous scores: Sensitivity and specificity determined? |  |
| **Physical Activity** | | | |
| Nord-Trøndelag Health Study PA Questionnaire (HUNT 1)^30^ | 1 | NA | 1 |
| Past Year Total Physical Activity Questionnaire (PYTPAQ)^31^ | 1 | NA | 1 |
| Physical Activity Assessment Tool (PAAT)^32^ | 1 | 1 | 1 |
| Minnesota Leisure Time Physical Activity Questionnaire (Minnesota LTPA Q)^33^ | 1 | NA | 1 |
| Single Item Physical Activity Measure (SI PA M)^34^ | NA | NA | NA |
| Godin Questionnaire (Godin Q)^33^ | 1 | NA | 1 |
| CARDIA Physical Activity History (CARDIA)^33^ | 1 | NA | 1 |
| College Alumnus Questionnaire (College Alumnus Q)^33^ | 1 | NA | 1 |
| Minnesota Heart Health Program Questionnaire (MHHP Q)^33^ | 1 | NA | 1 |
| Modified Historical Leisure Activity Questionnaire (MHLAQ)^35^ | NA | NA | NA |
| Modified version Active Australia Survey 1(MV – AAS1)^36^ | 1 | 4 | 4 |
| Modified version Active Australia Survey (MV – AAS2)^37^ | 1 | 4 | 4 |
| Adapted from Active Australia Survey (Adapt AAS)^38^ | 1 | 4 | 4 |
| International Physical Activity Questionnaire – Walking Section (IPAQ-WS)^39^ | 1 | NA | 1 |
| Short Questionnaire to Assess Health-enhancing physical activity (SQUASH)^40^ | 1 | NA | 1 |
| European Prospective Investigation into Cancer and Nutrition Physical Activity Questionnaire (EPIC PAQ)^41^ | 1 | 4 | 4 |
| 13-Item Physical Activity Questionnaire (13I-PAQ)^42^ | NA | NA | NA |
| Questionnaire d’Activité Physique pour les Personnes Âgées (QAPPA)^43^ | NA | NA | NA |
| Incidental and Planned Exercise Questionnaire (IPEQ)^44^ | NA | NA | NA |
| Physical Activity Questionnaire for Elderly Japanese (PAQ-EJ)^45^ | 1 | 4 | 4 |
| The Longitudinal Ageing Study Amsterdam Physical Activity Questionnaire (LAPAQ)^46^ | 1 | NA | 1 |
| Nordic Physical Activity Questionnaire (NPAQ-short)^47^ | 1 | 1 | 1 |
| Self-report physical activity questionnaire (SPAQ)^48^ | 1 | NA | 1 |
| Transport and Physical Activity Questionnaire (TPAQ)^49^ | 1 | NA | 1 |
| General Practice Physical Activity Questionnaire (GPPAQ)^50^ | 4 | 1 | 4 |
| **Sedentary Behaviour** | | | |
| International Physical Activity Questionnaire - Sedentary Behaviour (IPAQ-SB)^51^ | 1 | NA | 1 |
| Australian Longitudinal Study on Women’s Health - Sedentary Behaviour Questions (ALSWH - SB Q)^52^ | NA | NA | NA |
| Self-reported sitting and breaks from sitting in the workplace (SBSW)^53^ | 1 | NA | 1 |
| Workplace Sitting Breaks Questionnaire (SITBRQ)^54^ | 1 | NA | 1 |
| Sedentary Behavior Questionnaire (SBQ)^55^ | 1 | NA | 1 |
| SED-GIH^56^ | 1 | NA | 1 |
| Workforce Sitting Questionnaire (WSQ)^57^ | 1 | NA | 1 |
| Japanese-Language Self-reported Measures for Assessing Adults Domain-Specific Sedentary Time (JSRM - SB)^58^ | 1 | NA | 1 |
| Longitudinal Aging Study Amsterdam questionnaire (LASA)^59^ | 1 | NA | 1 |
| SB question of the Yale Physical Activity Survey (YPAS - SB)^60^ | 1 | NA | 1 |
| Community Health Activities Model Program for Seniors SB questions (CHAMPS - SB)^60^ | 1 | NA | 1 |
| Cancer Prevention Study-3 Sedentary Time Survey (CPS-3 ST)^61^ | 1 | NA | 1 |
| **Sleep** | | | |
| BRFSS sleep questions (BRFSS Sleep)^62^ | 4 | NA | 4 |
| **Physical Activity + Sedentary Behaviour** | | | |
| Kaiser Physical Activity Survey (KPAS)^63^ | 1 | NA | 1 |
| Sedentary, Transportation and Activity Questionnaire (STAQ)^64^ | 1 | NA | 1 |
| International Physical Activity Questionnaire (IPAQ)^65^ | 1 | 1 | 1 |
| Australian Women’s Activity Survey (AWAS)^66^ | 1 | NA | 1 |
| Workers’ sitting- and walking-time questionnaire Time Method (WSWQ- t-method)^67^ | 1 | NA | 1 |
| The Physical Activity Scale for the Elderly (PASE)^68^ | NA | NA | NA |
| Community Health Activities Model Program for Seniors physical activity self-report questionnaire + transport items (CHAMPS+transport)^69^ | 1 | NA | 1 |
| Community Healthy Activities Model Program for Seniors (CHAMPS)^70^ | NA | NA | NA |
| Modified Version of the MONICA Optional Study on Physical Activity Questionnaire (Modified MOSPA-Q)^71^ | 1 | NA | 1 |
| Occupational Sitting and Physical Activity Questionnaire (OSPAQ)^71,72^ | 1 | NA | 1 |
| Rapid Assessment Disuse Index (RADI)^73^ | 1 | NA | 1 |
| Global Physical Activity Questionnaire (GPAQ)^74^ | 1 | 1 | 1 |
| **Sedentary Behaviour + Sleep** | | | |
| SIT-Q^75^ | NA | NA | NA |
| **Physical Activity + Sedentary Behaviour + Sleep** | | | |
| Physical Activity Questionnaire (PAQ)^76^ | NA | NA | NA |
| Athens Physical Activity Questionnaire (APAQ)^77^ | 1 | NA | 1 |
| Sedentary Time and Activity Reporting Questionnaire (STAR-Q)^78^ | NA | NA | NA |
| Question 8 of the Paffenbarger Physical Activity Questionnaire (Q 8 PPAQ)^79^ | 1 | NA | 1 |
| EPIC-Norfolk Physical Activity Questionnaire (EPAQ2)^80^ | 1 | NA | 1 |
| Workers’ sitting- and walking-time questionnaire Percentage Method (WSWQ - p-method)^67^ | 1 | NA | 1 |
| New Questionnaire on Physical Activity (NQPA)^81^ | NA | NA | NA |
| Web-Based Physical Activity Questionnaire (Active-Q)^81^ | 1 | NA | 1 |
| Flemish Physical Activity Computerized Questionnaire (FPACQ)^83^ | 1 | NA | 1 |

Ratings: 1= Very low Risk of Bias; 2= Low Risk of Bias; 3= Medium Risk of Bias; 4= High Risk of Bias; Abbreviations: NA= Not Applicable.

* According to COSMIN guidelines, overall quality rating is based on ‘worst counts method’.

**Reliability and Measurement error**

| **Questionnaire** | Design Requirements (for reliability and measurement error) | | | Reliability Statistical Methods | | | | Measurement Error Statistical Methods | | Overall Rating Reliability/Measurement Error* |
| --- | --- | --- | --- | --- | --- | --- | --- | --- | --- | --- |
|  | 1.Participants stable during measurement period? | 2.Time interval appropriate? | 3.Similar test conditions in both measurements? | 4.Continuous scores: ICC calculated? | 5.Dichotomous/nominal/ ordinal scores: Kappa calculated? | 6.Ordinal scores: weighted kappa calculated? | 7. Ordinal scores: Weighting scheme described? | 4.Continuous scores: SEM, SDC or LoA calculated? | 5.Dichotomous/nominal/ordinal scores: Percentage (positive and negative) agreement calculated? |  |
| **Physical Activity** | | | | | | | | | | |
| Nord-Trøndelag Health Study PA Questionnaire (HUNT 1)^30^ | 1 | 1 | 2 | 3 | 1 | 1 | 2 | N.R. | N.R. | 3/N.R. |
| Past Year Total Physical Activity Questionnaire (PYTPAQ)^31^ | 1 | 1 | 1 | 1 | NA | NA | NA | N.R. | NA | 1/N.R. |
| Physical Activity Assessment Tool (PAAT)^32^ | 1 | 1 | 1 | 3 | 1 | NA | NA | N.R. | N.R. | 3/N.R. |
| Minnesota Leisure Time Physical Activity Questionnaire (Minnesota LTPA Q)^33^ | 1 | 1 | 1 | 3 | NA | NA | NA | N.R. | NA | 3/N.R. |
| Single Item Physical Activity Measure (SI PA M)^34^ | 1 | 1 | 1 | 3 | 1 | 1 | 2 | N.R. | N.R. | 3/N.R. |
| Godin Questionnaire (Godin Q)^33^ | 1 | 1 | 1 | 3 | NA | NA | NA | N.R. | NA | 4/N.R. |
| CARDIA Physical Activity History (CARDIA)^33^ | 1 | 1 | 1 | 3 | NA | NA | NA | N.R. | NA | 3/N.R. |
| College Alumnus Questionnaire (College Alumnus Q)^33^ | 1 | 1 | 1 | 3 | NA | NA | NA | N.R. | NA | 3/N.R. |
| Minnesota Heart Health Program Questionnaire (MHHP Q)^33^ | 1 | 1 | 1 | 3 | NA | NA | NA | N.R. | NA | 3/N.R. |
| Modified Historical Leisure Activity Questionnaire (MHLAQ)^35^ | 2 | 1 | 2 | 1 | NA | NA | NA | N.R. | NA | 2/N.R. |
| Modified version Active Australia Survey 1(MV – AAS1)^36^ | 1 | 1 | 1 | 3 | 4 | NA | NA | 2 | 2 | 4/2 |
| Modified version Active Australia Survey (MV – AAS2)^37^ | 1 | 4 | 2 | 4 | 1 | NA | NA | 1 | N.R. | 4/4 |
| Adapted from Active Australia Survey (Adapt AAS)^38^ | 1 | 1 | 2 | 1 | 1 | NA | NA | N.R. | 1 | 2/2 |
| International Physical Activity Questionnaire – Walking Section (IPAQ-WS)^39^ | 1 | 1 | 2 | 4 | NA | NA | NA | N.R. | NA | 4/N.R. |
| Short Questionnaire to Assess Health-enhancing physical activity (SQUASH)^40^ | 1 | 1 | 1 | 3 | 1 | 1 | 1 | N.R. | NA | 3/N.R. |
| European Prospective Investigation into Cancer and Nutrition Physical Activity Questionnaire (EPIC PAQ)^41^ | 1 | 1 | 1 | 3 | 1 | 1 | 1 | 1 | N.R. | 3/1 |
| 13-Item Physical Activity Questionnaire (13I-PAQ)^42^ | 1 | 1 | 2 | 1 | NA | NA | NA | N.R. | NA | 2/N.R. |
| Questionnaire d’Activité Physique pour les Personnes Âgées (QAPPA)^43^ | 1 | 4 | 2 | 1 | 1 | NA | NA | 1 | 2 | 4/4 |
| Incidental and Planned Exercise Questionnaire (IPEQ)^44^ | 1 | 1 | 2 | 1 | NA | NA | NA | N.R. | NA | 2/N.R. |
| Physical Activity Questionnaire for Elderly Japanese (PAQ-EJ)^45^ | 1 | 1 | 3 | 3 | NA | NA | NA | N.R. | NA | 3/N.R. |
| The Longitudinal Ageing Study Amsterdam Physical Activity Questionnaire (LAPAQ)^46^ | 1 | 1 | 1 | 3 | NA | NA | NA | 1 | NA | 3/1 |
| Nordic Physical Activity Questionnaire (NPAQ-short)^47^ | 1 | 1 | 1 | 3 | 1 | NA | NA | N.R. | N.R. | 3/N.R. |
| Self-report physical activity questionnaire (SPAQ)^48^ | 1 | 1 | 1 | 3 | NA | NA | NA | N.R. | NA | 3/N.R. |
| Transport and Physical Activity Questionnaire (TPAQ)^49^ | 1 | 1 | 2 | 1 | 1 | NA | NA | 2 | N.R. | 2/2 |
| General Practice Physical Activity Questionnaire (GPPAQ)^50^ | 1 | 4 | 1 | NA | 1 | 1 | 1 | N.R. | 2 | 4/4 |
| **Sedentary Behaviour** | | | | | | | | | | |
| International Physical Activity Questionnaire - Sedentary Behaviour (IPAQ-SB)^51^ | 1 | 1 | 1 | 3 | 1 | NA | NA | N.R. | N.R. | 3/N.R. |
| Australian Longitudinal Study on Women’s Health - Sedentary Behaviour Questions (ALSWH - SB Q)^52^ | 1 | 1 | 1 | 1 | NA | NA | NA | 2 | NA | 1/2 |
| Self-reported sitting and breaks from sitting in the workplace (SBSW)^53^ | 1 | 1 | 2 | 1 | NA | NA | NA | N.R. | NA | 2/N.R. |
| Workplace Sitting Breaks Questionnaire (SITBRQ)^54^ | **1** | 1 | 2 | 3 | 1 | 1 | 1 | N.R. | 2 | 3/2 |
| Sedentary Behavior Questionnaire (SBQ)^55^ | 1 | 1 | 2 | 1 | NA | NA | NA | N.R. | NA | 2/N.R. |
| SED-GIH^56^ | 4 | 1 | 4 | 1 | 1 | 1 | 1 | N.R. | N.R. | 4/N.R. |
| Workforce Sitting Questionnaire (WSQ)^57^ | 1 | 1 | 1 | 1 | NA | NA | NA | N.R. | NA | 1/N.R. |
| Japanese-Language Self-reported Measures for Assessing Adults Domain-Specific Sedentary Time (JSRM - SB)^58^ | 1 | 1 | 1 | 1 | NA | NA | NA | N.R. | NA | 1/N.R. |
| Longitudinal Aging Study Amsterdam questionnaire (LASA)^59^ | 1 | 1 | 1 | 1 | NA | NA | NA | N.R. | NA | 1/N.R. |
| SB question of the Yale Physical Activity Survey (YPAS - SB)^60^ | 1 | 1 | 1 | 1 | 1 | NA | NA | N.R. | NA | 1/N.R. |
| Community Health Activities Model Program for Seniors SB questions (CHAMPS - SB)^60^ | 1 | 1 | 1 | 1 | NA | NA | NA | N.R. | NA | 1/N.R. |
| Cancer Prevention Study-3 Sedentary Time Survey (CPS-3 ST)^61^ | 1 | 1 | 1 | 3 | NA | NA | NA | N.R. | NA | 3/N.R. |
| **Sleep** | | | | | | | | | | |
| BRFSS sleep questions (BRFSS Sleep)^62^ | 1 | 1 | 3 | 4 | NA | NA | NA | N.R. | NA | 4/N.R. |
| **Physical Activity + Sedentary Behaviour** | | | | | | | | | | |
| Kaiser Physical Activity Survey (KPAS)^63^ | 1 | 1 | 1 | 1 | NA | NA | NA | N.R. | NA | 1/N.R. |
| Sedentary, Transportation and Activity Questionnaire (STAQ)^64^ | 1 | 1 | 1 | 1 | NA | NA | NA | 2 | NA | 1/2 |
| International Physical Activity Questionnaire (IPAQ)^65^ | 1 | 1 | 2 | 3 | 4 | NA | NA | N.R. | 2 | 4/2 |
| Australian Women’s Activity Survey (AWAS)^66^ | 1 | 1 | 1 | 1 | NA | NA | NA | 2 | NA | 1/2 |
| Workers’ sitting- and walking-time questionnaire Time Method (WSWQ- t-method)^67^ | 1 | 1 | 1 | 1 | 1 | NA | NA | N.R. | N.R. | 1/N.R. |
| The Physical Activity Scale for the Elderly (PASE)^68^ | 1 | 4 | 2 | 3 | NA | NA | NA | N.R. | NA | 4/N.R. |
| Community Health Activities Model Program for Seniors physical activity self-report questionnaire + transport items (CHAMPS+transport)^69^ | 1 | 4 | 1 | 1 | NA | NA | NA | N.R. | NA | 4/N.R. |
| Community Healthy Activities Model Program for Seniors (CHAMPS)^70^ | 1 | 4 | 1 | 1 | NA | NA | NA | N.R. | NA | 4/N.R. |
| Modified Version of the MONICA Optional Study on Physical Activity Questionnaire (Modified MOSPA-Q)^71^ | 1 | 1 | 4 | 1 | NA | NA | NA | N.R. | NA | 4/N.R. |
| Occupational Sitting and Physical Activity Questionnaire (OSPAQ)^71,72^ | 1 | 1 | 4 | 1 | NA | NA | NA | N.R. | NA | 4/N.R. |
| Rapid Assessment Disuse Index (RADI)^73^ | 1 | 1 | 2 | 1 | NA | NA | NA | N.R. | NA | 2/N.R. |
| Global Physical Activity Questionnaire (GPAQ)^74^ | 1 | 3 | 1 | 1 | NA | NA | NA | N.R. | NA | 3/N.R. |
| **Sedentary Behaviour + Sleep** | | | | | | | | | | |
| SIT-Q^75^ | 1 | 1 | 1 | 1 | 1 | 1 | 1 | 1 | 1 | 1/1 |
| **Physical Activity + Sedentary Behaviour + Sleep** | | | | | | | | | | |
| Physical Activity Questionnaire (PAQ)^76^ | 2 | 1 | 2 | 3 | NA | NA | NA | N.R. | NA | 3/N.R. |
| Athens Physical Activity Questionnaire (APAQ)^77^ | 1 | 1 | 2 | 1 | NA | NA | NA | 2 | NA | 2/2 |
| Sedentary Time and Activity Reporting Questionnaire (STAR-Q)^78^ | 1 | 1 | 1 | 1 | NA | NA | NA | N.R. | NA | 1/N.R. |
| Question 8 of the Paffenbarger Physical Activity Questionnaire (Q 8 PPAQ)^79^ | 1 | 1 | 1 | 1 | NA | NA | NA | 1 | NA | 1/1 |
| EPIC-Norfolk Physical Activity Questionnaire (EPAQ2)^80^ | 1 | 1 | 1 | 3 | 1 | 1 | NA | N.R. | 4 | 3/4 |
| Workers’ sitting- and walking-time questionnaire Percentage Method (WSWQ - p-method)^67^ | 1 | 1 | 1 | 1 | 1 | NA | NA | N.R. | N.R. | 1/N.R. |
| New Questionnaire on Physical Activity (NQPA)^81^ | 1 | 1 | 2 | 3 | NA | NA | NA | 2 | NA | 3/2 |
| Web-Based Physical Activity Questionnaire (Active-Q)^81^ | 1 | 1 | 1 | 1 | NA | NA | NA | N.R. | NA | 1/N.R. |
| Flemish Physical Activity Computerized Questionnaire (FPACQ)^83^ | 1 | 1 | 1 | 1 | NA | NA | NA | 2 | NA | 1/2 |

Ratings: 1= Very low Risk of Bias; 2= Low Risk of Bias; 3= Medium Risk of Bias; 4= High Risk of Bias. Abbreviations: NA = Not Applicable; N.R.= Not Reported

* According to COSMIN guidelines, overall quality rating is based on “worst counts method”.

**Convergent Validity**

| **Questionnaire (Convergent measure)** | Design Requirements | | Statistical Methods | Overall Rating* |  |
| --- | --- | --- | --- | --- | --- |
|  | Clarity on the comparator instrument(s) measure(s)? | Measurement properties of the comparator instrument(s) are sufficient? | Were design and statistical methods adequate for the hypotheses to be tested? |  |  |
|  |  |  |  |  |  |
|  |  |  |  |  |  |
|  |  |  |  |  |  |
|  |  |  |  |  |  |
| **Physical Activity** | | | | |  |
| Nord-Trøndelag Health Study PA Questionnaire (HUNT 1)^30^ (IPAQ) | 1 | 1 | 1 | 1 |  |
| Nord-Trøndelag Health Study PA Questionnaire (HUNT 1)^30^ (VO2max) | 1 | 1 | 1 | 1 |  |
| Past Year Total Physical Activity Questionnaire (PYTPAQ)^31^ (7-day PA log) | 1 | 1 | 1 | 1 |  |
| Physical Activity Assessment Tool (PAAT)^32^ (IPAQ) | 1 | 1 | 1 | 1 |  |
| Minnesota Leisure Time Physical Activity Questionnaire (Minnesota LTPA Q)^33^ (4 wk history) | 1 | 1 | 1 | 1 |  |
| Minnesota Leisure Time Physical Activity Questionnaire (Minnesota LTPA Q)^33^ (VO2max) | 1 | 1 | 1 | 1 |  |
| Single Item Physical Activity Measure (SI PA M)^34^ (GPAQ) | 1 | 1 | 2 | 2 |  |
| Single Item Physical Activity Measure (SI PA M)^34^ (APS) | 1 | 1 | 2 | 2 |  |
| Godin Questionnaire (Godin Q)^33^ (4 wk history) | 1 | 1 | 1 | 1 |  |
| Godin Questionnaire (Godin Q)^33^ (VO2max) | 1 | 1 | 1 | 1 |  |
| CARDIA Physical Activity History (CARDIA)^33^ (4 wk history) | 1 | 1 | 1 | 1 |  |
| CARDIA Physical Activity History (CARDIA)^33^ (VO2max) | 1 | 1 | 1 | 1 |  |
| Minnesota Heart Health Program Questionnaire (MHHP Q)^33^ (4 wk history) | 1 | 1 | 1 | 1 |  |
| Minnesota Heart Health Program Questionnaire (MHHP Q)^33^ (VO2max) | 1 | 1 | 1 | 1 |  |
| Modified Historical Leisure Activity Questionnaire (MHLAQ)^35^ (PA LOG) | 1 | 1 | 1 | 1 |  |
| Modified version Active Australia Survey 1(MV – AAS1)^36^ | NA | NA | NA | NA |  |
| (MV – AAS2)^8^ | NA | NA | NA | NA |  |
| Adapt AAS^9^ | NA | NA | NA | NA |  |
| IPAQ-WS^10^ | NA | NA | NA | NA |  |
| SQUASH^11^ | NA | NA | NA | NA |  |
| European Prospective Investigation into Cancer and Nutrition Physical Activity Questionnaire (EPIC PAQ)^41^ (FriedeN.R.eich LTPAQ) | 1 | 1 | 1 | 1 |  |
| 13-Item Physical Activity Questionnaire (13I-PAQ)^42^ (Body fat) | 1 | 1 | 2 | 2 |  |
| 13-Item Physical Activity Questionnaire (13I-PAQ)^42^ (VO2max) | 1 | 1 | 2 | 2 |  |
| 13-Item Physical Activity Questionnaire (13I-PAQ)^42^ (Flexibility) | 1 | 1 | 2 | 2 |  |
| Questionnaire d’Activité Physique pour les Personnes Âgées (QAPPA)^43^ (Satisfaction with body functioning) | 1 | 4 | 2 | 4 |  |
| Questionnaire d’Activité Physique pour les Personnes Âgées (QAPPA)^43^ (Decline in physical function) | 1 | 4 | 2 | 4 |  |
| Questionnaire d’Activité Physique pour les Personnes Âgées (QAPPA)^43^ (Age) | 1 | 4 | 2 | 4 |  |
| Incidental and Planned Exercise Questionnaire (IPEQ)^44^ (IPEQ-3 months v IPEQ-Week) | 1 | 4 | 2 | 4 |  |
| Incidental and Planned Exercise Questionnaire (IPEQ)^44^ (WHODAS) | 1 | 4 | 2 | 4 |  |
| Incidental and Planned Exercise Questionnaire (IPEQ)^44^ (Time up-and-go) | 1 | 4 | 2 | 4 |  |
| Incidental and Planned Exercise Questionnaire (IPEQ)^44^ (Age) | 1 | 4 | 2 | 4 |  |
| Physical Activity Questionnaire for Elderly Japanese (PAQ-EJ)^45^ | NA | NA | NA | NA |  |
| The Longitudinal Ageing Study Amsterdam Physical Activity Questionnaire (LAPAQ)^46^ | NA | NA | NA | NA |  |
| Nordic Physical Activity Questionnaire (NPAQ-short)^47^ | NA | NA | NA | NA |  |
| Self-report physical activity questionnaire (SPAQ)^48^ | NA | NA | NA | NA |  |
| Transport and Physical Activity Questionnaire (TPAQ)^49^ | NA | NA | NA | NA |  |
| General Practice Physical Activity Questionnaire (GPPAQ)^50^ | NA | NA | NA | NA |  |
| **Sedentary Behaviour** | | | | |  |
| International Physical Activity Questionnaire - Sedentary Behavior (IPAQ-SB)^51^ | NA | NA | NA | NA |  |
| Australian Longitudinal Study on Women’s Health - Sedentary Behavior Questions (ALSWH - SB Q)^52^ (7-d behavior log) | 1 | 1 | 1 | 1 |  |
| Self-reported sitting and breaks from sitting in the workplace (SBSW) ^53^ | NA | NA | NA | NA |  |
| Workplace Sitting Breaks Questionnaire (SITBRQ)^54^ | NA | NA | NA | NA |  |
| Sedentary Behavior Questionnaire (SBQ)^55^ | 1 | 1 | 1 | 1 |  |
| SED-GIH^56^ | NA | NA | NA | NA |  |
| Workforce Sitting Questionnaire (WSQ)^57^ | NA | NA | NA | NA |  |
| Japanese-Language Self-reported Measures for Assessing Adults Domain-Specific Sedentary Time (JSRM - SB)^58^ | NA | NA | NA | NA |  |
| Longitudinal Aging Study Amsterdam questionnaire (LASA) ^59^ | NA | NA | NA | NA |  |
| SB question of the Yale Physical Activity Survey (YPAS - SB)^60^ | NA | NA | NA | NA |  |
| Community Health Activities Model Program for Seniors SB questions (CHAMPS-SB) ^60^ | NA | NA | NA | NA |  |
| Cancer Prevention Study-3 Sedentary Time Survey (CPS-3 sitting time)^61^ | NA | NA | NA | NA |  |
| **Sleep** | | | | |  |
| BRFSS sleep questions (BRFSS Sleep)^62^ (Theorized question) | 1 | 4 | 4 | 4 |  |
| **Physical Activity + Sedentary Behaviour** | | | | |  |
| Kaiser Physical Activity Survey (KPAS)^63^ (PA record) | 1 | 1 | 1 | 1 |  |
| Sedentary, Transportation and Activity Questionnaire (STAQ)^64^ (Contextualized logbook) | 1 | 1 | 1 | 1 |  |
| International Physical Activity Questionnaire (IPAQ)^65^ (IPAQ Short forms) | 1 | 1 | 1 | 1 |  |
| Australian Women’s Activity Survey (AWAS)^66^ | NA | NA | NA | NA |  |
| Workers’ sitting- and walking-time questionnaire Time Method (WSWQ- t-method)^67^ | NA | NA | NA | NA |  |
| The Physical Activity Scale for the Elderly (PASE)^68^ (Perceived health) | 1 | 1 | 1 | 1 |  |
| The Physical Activity Scale for the Elderly (PASE)^68^ (Sick Impact Profile) | 1 | 1 | 1 | 1 |  |
| The Physical Activity Scale for the Elderly (PASE)^68^ (Grip Strength) | 1 | 1 | 1 | 1 |  |
| The Physical Activity Scale for the Elderly (PASE)^68^ (Balance) | 1 | 1 | 1 | 1 |  |
| Community Health Activities Model Program for Seniors physical activity self-report questionnaire + transport items (CHAMPS+transport)^69^ | NA | NA | NA | NA |  |
| Community Healthy Activities Model Program for Seniors (CHAMPS)^70^ (6-Min Walk) | 1 | 1 | 1 | 1 |  |
| Community Healthy Activities Model Program for Seniors (CHAMPS)^70^ (Self-Reported Physical Functioning) | 1 | 1 | 1 | 1 |  |
| Modified Version of the MONICA Optional Study on Physical Activity Questionnaire (Modified MOSPA-Q)^71^ | NA | NA | NA | NA |  |
| Occupational Sitting and Physical Activity Questionnaire (OSPAQ)^71,72^ | NA | NA | NA | NA |  |
| Occupational Sitting and Physical Activity Questionnaire (OSPAQ)^71,72^ | 1 | 1 | 1 | 1 |  |
| Rapid Assessment Disuse Index (RADI)^73^ | NA | NA | NA | NA |  |
| Global Physical Activity Questionnaire (GPAQ)^74^ (IPAQ) | 1 | 1 | 1 | 1 |  |
| **Sedentary Behaviour + Sleep** | | | | |  |
| SIT-Q^75^ | 1 | 1 | 1 | 1 |  |
| **Physical Activity + Sedentary Behaviour + Sleep** | | | | |  |
| Physical Activity Questionnaire (PAQ)^76^ (Self-administered structured 7-day PA diary) | 1 | 1 | 1 | 1 |  |
| Athens Physical Activity Questionnaire (APAQ)^77^ | NA | NA | NA | NA |  |
| Sedentary Time and Activity Reporting Questionnaire (STAR-Q)^78^ | 1 | 1 | 1 | 1 |  |
| Question 8 of the Paffenbarger Physical Activity Questionnaire (Q 8 PPAQ)^79^ | NA | NA | NA | NA |  |
| EPIC-Norfolk Physical Activity Questionnaire (EPAQ2)^80^ (VO2max) | 1 | 1 | 1 | 1 |  |
| Workers’ sitting- and walking-time questionnaire Percentage Method (WSWQ - p-method)^67^ | NA | NA | NA | NA |  |
| New Questionnaire on Physical Activity (NQPA)^81^ (PA Diary) | 1 | 1 | 1 | 1 |  |
| Web-Based Physical Activity Questionnaire (Active-Q)^82^ | NA | NA | NA | NA |  |
| Flemish Physical Activity Computerized Questionnaire (FPACQ) ^83^ | NA | NA | NA | NA |  |

Ratings: 1= Very low Risk of Bias; 2= Low Risk of Bias; 3= Medium Risk of Bias; 4= High Risk of Bias. Abbreviations: NA = Not Applicable.

* According to Cosmin, overall quality rating is based on “worst counts method”.

**Responsiveness**

| **Questionnaire** | Design Requirements | Statistical Methods | Other | Overall Rating |  |
| --- | --- | --- | --- | --- | --- |
|  | Adequate description of important characteristics of the subgroups provided? | Were design and statistical methods adequate for the hypotheses to be tested? | Any other important flaws? |  |  |
|  |  |  |  |  |  |
|  |  |  |  |  |  |
|  |  |  |  |  |  |
|  |  |  |  |  |  |
| Community Healthy Activities Model Program for Seniors (CHAMPS)^70^ | 3 | 1 |  | 3 |  |

Ratings: 1= Very low Risk of Bias; 2= Low Risk of Bias; 3= Medium Risk of Bias; 4= High Risk of Bias

* According to Cosmin, overall quality rating is based on “worst counts method”.

**References**

30. Kurtze N, Rangul V, Hustvedt B, Flanders WD. Reliability and validity of self-reported physical activity in the Nord-Trøndelag Health Study -- HUNT 1. Scandinavian Journal of Public Health. 2008;36(1):52-61. doi:10.1177/1403494807085373

31. FriedeN.R.eich CM, Courneya KS, Neilson HK, et al. Reliability and validity of the Past Year Total Physical Activity Questionnaire. American Journal of Epidemiology. 2006;163(10):959-970. doi:aje/kwj112

32. Meriwether RA, McMahon PM, Islam N, Steinmann WC. Physical Activity Assessment: Validation of a Clinical Assessment Tool. American Journal of Preventive Medicine. 2006;31(6):484-491. doi:10.1016/j.amepre.2006.08.021

33. Jacobs DR, Ainsworth BE, Hartman TJ, Leon AS. A simultaneous evaluation of 10 commonly used physical activity questionnaires. / Evaluation simultanee de 10 questionnaires couramment utilises sur les activites physiques. Medicine & Science in Sports & Exercise. 1993;25(1):81-91.

34. Milton K, Bull FC, Bauman A. Reliability and validity testing of a single-item physical activity measure. British Journal of Sports Medicine. 2011;45(3):203-208.

35. Chasean-Taber L, Erickson JB, Nasca PC, Chasan-Taber S, Freedson PS. Validity and reproducibility of a physical activity questionnaire in women. / Validite et reproductibilite d ' un questionnaire sur l ' activite physique chez des femmes. Medicine & Science in Sports & Exercise. 2002;34(6):987-992.

36. Brown WJ, Burton NW, Marshall AL, Miller YD. Reliability and validity of a modified self-administered version of the Active Australia physical activity survey in a sample of mid-age women. Australian & New Zealand Journal of Public Health. 2008;32(6):535-541.

37. Fjeldsoe BS, Winkler EAH, Marshall AL, Eakin EG, Reeves MM. Active adults recall their physical activity differently to less active adults: test-retest reliability and validity of a physical activity survey. Health Promotion Journal of Australia. 2013;24(1):26-31. doi:10.1071/HE12912

38. Timperio A, Salmon J, Crawford D. Validity and reliability of a physical activity recall instrument among overweight and non-overweight men and women. Journal of Science & Medicine in Sport. 2003;6(4):477-491.

39. van der Ploeg HP, Tudor-Locke C, Marshall AL, et al. Reliability and validity of the international physical activity questionnaire for assessing walking. Res Q Exerc Sport. Mar 2010;81(1):97-101. doi:10.1080/02701367.2010.10599632

40. Wendel-Vos GW, Schuit AJ, Saris WH, Kromhout D. Reproducibility and relative validity of the short questionnaire to assess health-enhancing physical activity. Journal of clinical epidemiology. 2003;56(12):1163-1169.

41. Cust AE, Smith BJ, Chau J, et al. Validity and repeatability of the EPIC Physical Activity Questionnaire: A validation study using accelerometers as an objective measure. The international journal of behavioral nutrition and physical activity. 2008;5

42. Nikolaidis PT, Säcklova M. Validity against health-related fitness and reliability of physical activity questionnaire in young female and male adults. Journal of Physical Education & Sport. 2011;11(3):342-348.

43. de Souto Barreto P. Construct and convergent validity and repeatability of the Questionnaire d’Activité Physique pour les Personnes Âgées (QAPPA), a physical activity questionnaire for the elderly. Public Health. 2013;127(9):844-853. doi:10.1016/j.puhe.2012.10.018

44. Delbaere K. Evaluation of the incidental and planned activity questionnaire for older people. British Journal of Sports Medicine. 2010;44(14):1029-1034.

45. Yasunaga A, Park H, Watanabe E, et al. Development and evaluation of the physical activity questionnaire for elderly Japanese: The Nakanojo study. Journal of Aging and Physical Activity. 2007;15(4):398-411. doi:10.1123/japa.15.4.398

46. Siebeling L, Wiebers S, Beem L, Puhan MA, Ter Riet G. Validity and reproducibility of a physical activity questionnaire for older adults: questionnaire versus accelerometer for assessing physical activity in older adults. Clinical epidemiology. 2012;4:171.

47. Danquah IH, Petersen CB, Skov SS, Tolstrup JS. Validation of the NPAQ-short - a brief questionnaire to monitor physical activity and compliance with the WHO recommendations. BMC Public Health. 2018;18(1):N.PAG-N.PAG. doi:10.1186/s12889-018-5538-y

48. Visuthipanich V, Sirapo-ngam Y, Malathum P, Kijboonchoo K, Vorapongsathorn T, Winters-Stone K. Physical activity questionnaire development and testing among elderly community-dwelling Thais. Thai Journal of Nursing Research. 2009;13(4):249-267.

49. Adams EJ, Goad M, Sahlqvist S, Bull FC, Cooper AR, Ogilvie D. Reliability and validity of the Transport and Physical Activity Questionnaire (TPAQ) for assessing physical activity behaviour. PLoS ONE. 2014;9(9)

50. Ahmad S, Harris T, Limb E, et al. Evaluation of reliability and validity of the General Practice Physical Activity Questionnaire (GPPAQ) in 60–74 year old primary care patients. BMC family practice. 2015;16(1):113.

51. Rosenberg DE, Bull FC, Marshall AL, Sallis JF, Bauman AE. Assessment of sedentary behavior with the International Physical Activity Questionnaire. Journal of Physical Activity & Health. 2008;5(Suppl1):S30-S44.

52. Marshall AL, Miller YD, Burton NW, Brown WJ. Measuring total and domain-specific sitting: a study of reliability and validity. Med Sci Sports Exerc. Jun 2010;42(6):1094-102. doi:10.1249/MSS.0b013e3181c5ec18

53. Sudholz B, Ridgers ND, Mussap A, Bennie J, Timperio A, Salmon J. Reliability and validity of self-reported sitting and breaks from sitting in the workplace. J Sci Med Sport. Jul 2018;21(7):697-701. doi:10.1016/j.jsams.2017.10.030

54. Pedisic Z, Bennie JA, Timperio AF, et al. Workplace Sitting Breaks Questionnaire (SITBRQ): an assessment of concurrent validity and test-retest reliability. BMC Public Health. Dec 5 2014;14:1249. doi:10.1186/1471-2458-14-1249

55. Rosenberg DE, Norman GJ, Wagner N, Patrick K, Calfas KJ, Sallis JF. Reliability and validity of the Sedentary Behavior Questionnaire (SBQ) for adults. Journal of Physical Activity & Health. 2010;7(6):697-705. doi:10.1123/jpah.7.6.697

56. Larsson K, Kallings LV, Ekblom Ö, Blom V, Andersson E, Ekblom MM. Criterion validity and test-retest reliability of SED-GIH, a single item question for assessment of daily sitting time. BMC public health. 2019;19(1):17.

57. Chau JY, Van Der Ploeg HP, Dunn S, Kurko J, Bauman AE. A tool for measuring workers' sitting time by domain: the Workforce Sitting Questionnaire. British journal of sports medicine. 2011;45(15):1216-1222.

58. Ishii K, Shibata A, Kurita S, et al. Validity and reliability of Japanese-language self-reported measures for assessing adults domain-specific sedentary time. Journal of epidemiology. 2017:JE20170002.

59. Visser M, Koster A. Development of a questionnaire to assess sedentary time in older persons–a comparative study using accelerometry. BMC geriatrics. 2013;13(1):80.

60. Gennuso KP, Matthews CE, Colbert LH. Reliability and validity of 2 self-report measures to assess sedentary behavior in older adults. Journal of Physical Activity & Health. 2015;12(5):727-732. doi:10.1123/jpah.2013-0546

61. Rees-Punia E, Matthews CE, Evans EM, et al. Demographic-specific validity of the cancer prevention study-3 sedentary time survey. Medicine and science in sports and exercise. 2019;51(1):41.

62. Jungquist CR, Mund J, Aquilina AT, et al. Validation of the Behavioral Risk Factor Surveillance System Sleep Questions. J Clin Sleep Med. Mar 2016;12(3):301-10. doi:10.5664/jcsm.5570

63. Ainsworth BE, Sternfeld B, Richardson MT, Jackson K. Evaluation of the Kaiser Physical Activity Survey in women. Medicine & Science in Sports & Exercise. 2000;32(7):1327-1334.

64. Mensah K, Maire A, Oppert J-M, et al. Assessment of sedentary behaviors and transport-related activities by questionnaire: a validation study. BMC Public Health. 2016;16(1):1-9. doi:10.1186/s12889-016-3412-3

65. Craig CL, Marshall AL, Sjöström M, et al. International physical activity questionnaire: 12-country reliability and validity. Med Sci Sports Exerc. Aug 2003;35(8):1381-95. doi:10.1249/01.mss.0000078924.61453.fb

66. Fjeldsoe BS, Marshall AL, Miller YD. Measurement properties of the Australian Women's Activity Survey. Medicine & Science in Sports & Exercise. 2009;41(5):1020-1033. doi:10.1249/MSS.0b013e31819461c2

67. Matsuo T, Sasai H, So R, Ohkawara K. Percentage-method improves properties of workers’ sitting-and walking-time questionnaire. Journal of epidemiology. 2016;26(8):405-412.

68. Washburn RA, Smith KW, Jette AM, Janney CA. The Physical Activity Scale for the Elderly (PASE): Development and evaluation. Journal of Clinical Epidemiology. 1993;46(2):153-162. doi:10.1016/0895-4356(93)90053-4

69. Hekler EB, Buman MP, Haskell WL, et al. Reliability and validity of CHAMPS self-reported sedentary-to-vigorous intensity physical activity in older adults. Journal of Physical Activity and Health. 2012;9(2):225-236.

70. Stewart AL, Mills KM, King AC, Haskell WL, Gillis D, Ritter PL. CHAMPS physical activity questionnaire for older adults: outcomes for interventions. / CHAMPS: Questionnaire sur les activites physiques des personnes agees: resultats pour de futures interventions. Medicine & Science in Sports & Exercise. 2001;33(7):1126-1141.

71. Chau JY, Van Der Ploeg HP, Dunn S, Kurko J, Bauman AE. Validity of the Occupational Sitting and Physical Activity Questionnaire. Medicine & Science in Sports & Exercise. 2012;44(1):118-125.

72. Pedersen SJ, Kitic CM, Bird M-L, Mainsbridge CP, Cooley PD. Is self-reporting workplace activity worthwhile? Validity and reliability of occupational sitting and physical activity questionnaire in desk-based workers. BMC Public Health. 2016;16(1):836-836. doi:10.1186/s12889-016-3537-4

73. Shuval K, Harold WK, III, Bernstein I, et al. Sedentary behaviour and physical inactivity assessment in primary care: the Rapid Assessment Disuse Index (RADI) study. British Journal of Sports Medicine. 2014;48(3):250-255.

74. Bull FC, Maslin TS, Armstrong T. Global Physical Activity Questionnaire (GPAQ): Nine country reliability and validity study. Journal of Physical Activity & Health. 2009;6(6):790-804.

75. Lynch BM, FriedeN.R.eich CM, Khandwala F, Liu A, Nicholas J, Csizmadi I. Development and testing of a past year measure of sedentary behavior: the SIT-Q. BMC Public Health. Sep 1 2014;14:899. doi:10.1186/1471-2458-14-899

76. Norman A, Bellocco R, Bergström A, Wolk A. Validity and reproducibility of self-reported total physical activity--differences by relative weight. Int J Obes Relat Metab Disord. May 2001;25(5):682-8. doi:10.1038/sj.ijo.0801597

77. Kavouras SA, Maraki MI, Kollia M, Gioxari A, Jansen LT, Sidossis LS. Development, reliability and validity of a physical activity questionnaire for estimating energy expenditure in Greek adults. Science & Sports. 2016;31(3):e47-e53.

78. Csizmadi I, Neilson HK, Kopciuk KA, et al. The Sedentary Time and Activity Reporting Questionnaire (STAR-Q): reliability and validity against doubly labeled water and 7-day activity diaries. American journal of epidemiology. 2014;180(4):424-435.

79. Simpson K, Parker B, Capizzi J, et al. Validity and Reliability of Question 8 of the Paffenbarger Physical Activity Questionnaire Among Healthy Adults. Journal of Physical Activity & Health. 2015;12(1):116-123.

80. Wareham NJ, Jakes RW, Rennie KL, Mitchell J, Hennings S, Day NE. Validity and repeatability of the EPIC-Norfolk physical activity questionnaire. International journal of epidemiology. 2002;31(1):168-174.

81. Pols MA, Peeters PH, Ocke MC, et al. Relative validity and repeatability of a new questionnaire on physical activity. Preventive Medicine. 1997;26(1):37-43.

82. Bonn SE, Bergman P, Lagerros YT, Sjölander A, Bälter K. A validation study of the web-based physical activity questionnaire active-Q against the GENEA accelerometer. JMIR research protocols. 2015;4(3):e86.

83. Matton L, Wijndaele K, Duvigneaud N, et al. Reliability and Validity of the Flemish Physical Activity Computerized Questionnaire in Adults. Research Quarterly for Exercise & Sport. 2007;78(4):293-306.
